# Organs-specific metabolomics and anticholinesterase activity suggests a trade-off between metabolites for therapeutic advantages of *Trillium govanianum* Wall. ex D. Don

**DOI:** 10.1038/s41598-024-61160-w

**Published:** 2024-05-09

**Authors:** Dinesh Kumar, Vandana Kumari, Dinesh Kumar

**Affiliations:** 1 Chemical Technology Division, CSIR-Institute of Himalayan Bioresource Technology, Palampur, HP 176 061 India; 2 Academy of Scientific and Innovative Research, Ghaziabad, Uttar Pradesh 201002 India

**Keywords:** *Trillium govanianum*, Organs-specific metabolomics, Steroids quantification, UHPLC-QTOF-IMS, Anti-cholinesterase, Metabolomics, Natural products, Analytical chemistry

## Abstract

*Trillium govanianum* is traditionally used to treat innumerable alignments like sexual disorders, cancer, inflammation etc. Mainly rhizomes of *T. govanianum* have been explored for phytochemical profiling but comprehensive metabolomics of other parts has not been yet deeply investigated. Thus, current study was aimed for organs-specific (roots, rhizomes, rhizomatous buds, stems, leaves, and fruits) phytochemical profiling of *T. govanianum *via metabolomics approach. Targeted (steroidal saponins and free sugars) and non-targeted metabolomics were performed by UPLC-PDA/ELSD & UHPLC-Q-TOF-IMS. Among steroidal compounds, 20-hydroxyecdysone, pennogenin-3-O-*β*-chacotrioside, dioscin were found predominantly in all samples while diosgenin was identified only in rhizomes. Further, four free sugars viz. 2-deoxyribose (116.24 ± 1.26 mg/g: leaves), fructose (454.76 ± 12.14 mg/g: rhizomes), glucose (243.21 ± 7.53 mg/g: fruits), and galactose (69.06 ± 2.14 mg/g: fruits) were found significant in respective parts of *T. govanianum*. Elemental analysis of targeted samples was determined by atomic absorption spectrophotometer. Heavy metals (Cd, Hg, Pd, As) were absent while micro- (Mn, Na, Zn, Cu) and macro- (Ca, Fe, Mg, K) elements were found in all samples. Furthermore, UHPLC-Q-TOF-IMS had identified 103 metabolites based on their mass fragmentation patterns and 839 were tentatively predicted using METLIN database. The multivariate statistical analysis showed organs specific clustering and variance of metabolites. Apart from this, extracts were evaluated for in vitro anticholinesterase activity, and found potentials inhibitors with IC_50_ values 2.02 ± 0.15 to 27.65 ± 0.89 mg/mL and 3.58 ± 0.12 to 16.81 ± 2.48 mg/mL of acetylcholinesterase (AChE) and butyrylcholinesterase (BChE) enzyme, respectively. Thus, comprehensive metabolomics and anti-cholinesterase activity of different parts of *T. govanianum* would lay the foundation for improving medicinal importance and health benefits of *T. govanianum.*

## Introduction

*Trillium govanianum* Wall. ex D. Don, a herb belonging to family Melanthiaceae has been used traditionally for the treatment of cancer, sepsis, neurasthenia, dysentery, backache, wounds, inflammation, skin boils, reproductive disorder, menstrual and sexual disorders^1,2^. It is commonly known as “Nag Chhatri” and distributed in the Himalaya from Nanga Parbat (Gilgit-Baltistan) to Namcha Barwa (Tibet) at an altitude of 2400–3500 m amsl^3^. Pharmacologically, this species was reported to have anticancer, antifungal, antioxidant, antidiabetic, and anti-inflammatory activities^4–6^. These medicinal properties might be attributed due to the presence of bioactive compounds like saponins, glycosides, terpenoids, phenolics, and flavonoids^7,8^. The phytochemical investigation of rhizomatous parts revealed to contain a high-value saponins of steroidal type like govanoside A, 20-hydroxyecdysone, pennogenin, and 5,20-dihydroxyecdysone^7,9,10^. Earlier reports also suggested the presence of diosgenin in *T. govanianum* rhizomes which is an important corticosteroid hormone that is utilized to form sex hormones and various steroidal drugs^11,12^. Although, in addition to rhizomes, aerial parts of *T. govanianum* also hold valuable importance. Earlier report on aerial parts (stems, leaves, fruits) had shown the presence of polyphenols as well as their free radicals scavenging and antidiabetic potential^5^. But, the comprehensive profiling of metabolites in the aerial parts are entirely unexplored. Thus, employment of analytical techniques is necessary to understand the metabolic flux in the aerial parts of *T. govanianum*.

Recently, metabolomics approach has been become as one of the most widely used and meaningful analytical technique for the comprehensive profiling of metabolites in plants^13^. Metabolomics study was accomplished by two strategies, i.e. untargeted and targeted compounds identification and quantification, which have been adopted by researchers^14^. However, untargeted approach gathers a huge coverage on metabolites from dataset account. The plant metabolomics is a most rational approach and performed by advanced analytical tool in combination with multivariate statistical analysis (MSA) to monitor quality, variability, and similarities among the different samples^15,16^. To date, various tools have been applied for metabolomics studies but mass spectrometry (MS)-coupled techniques provide a very high sensitivity and detection ability to less abundant metabolites. UHPLC-DAD-Q-TOF-MS^2^ is another choice of important tool for phytochemicals profiling in plant extracts^17–19^. Previously, metabolomics study was performed for ethanol and water extract of *T. govanianum* rhizomes only that described the identification of 26 metabolites^3^. Hence, in the current investigation, an UHPLC-QTOF-IMS was used for comprehensive metabolites profiling in aerial (stem, leaves, and fruits) and underground organs (roots, rhizomes, and rhizomatous buds) of *T. govanianum*. Both untargeted and targeted metabolomics studies were applied to understand specialized metabolites and their metabolic fluidity in different organs extracts. The widely known statistical inference were further used to analyse the variability in terms of similarities and differences among different samples. Multivariate statistical analysis including heatmaps, ven-diagram, stacked charts, principal component/coordinate analysis (PCA and PCoA) and hierarchical clustering analysis (HCA) were employed to screen out constituents that could help as part-specific markers of *T. govanianum*. Apart from this, acetylcholinesterase/butyrylcholinesterase inhibitory potential of different organs of *T. govanianum* was also investigated. To the best of our knowledge, this will be the first metabolomics assessment study that provide the metabolome information of whole plant*.* This report will shed light on the chemo-information, especially for ignored aerial parts (stem, leaves, and fruits). This is an important to understand its therapeutics and nutraceutical importance of underutilized parts.

## Results

In current metabolomics studies, fresh materials of different organs (stems, leaves, fruits, rhizomatous bud, roots, rhizomes) of *T. govanianum* were extracted with ethanol and extracts were subjected for metabolomics study (Fig. S1). For targeted analysis of steroidal compounds (compounds **1–5)**, an UPLC-PDA-based method was developed and validated as per ICH guidelines. The calibration curves of all five compounds (**1–5**) were found linear in concentration range of 3.906–500 µg/mL and coefficient of regression (r^2^) was in range of 0.994–0.998 (Fig. S2). Limit of detection (LOD) and limit of quantification (LOQ) of compounds **1–5** were found in the range 3.35–7.26 and 10.15–22.00, µg/mL respectively. The relative standard deviation (RSD) values were observed 0.44–1.23% and 0.75–2.08% for intraday (*n* = 3) and interday (*n* = 3) precisions, respectively (Table S1). Further, the recoveries study of each analyte was performed in ethanolic extract of rhizomes and found in the assortment of ˃80% (Table S1). The rt and area of the peak was found constant on repetitive conditions. These findings suggested that method is sensitive, stable, and accurate for the concurrent qualitative and quantitative analysis of compounds **1–5** in different parts of *T. govanianum*.

### Quantification of steroidal compounds (compounds 1–5) in different parts

**Figure 1:**
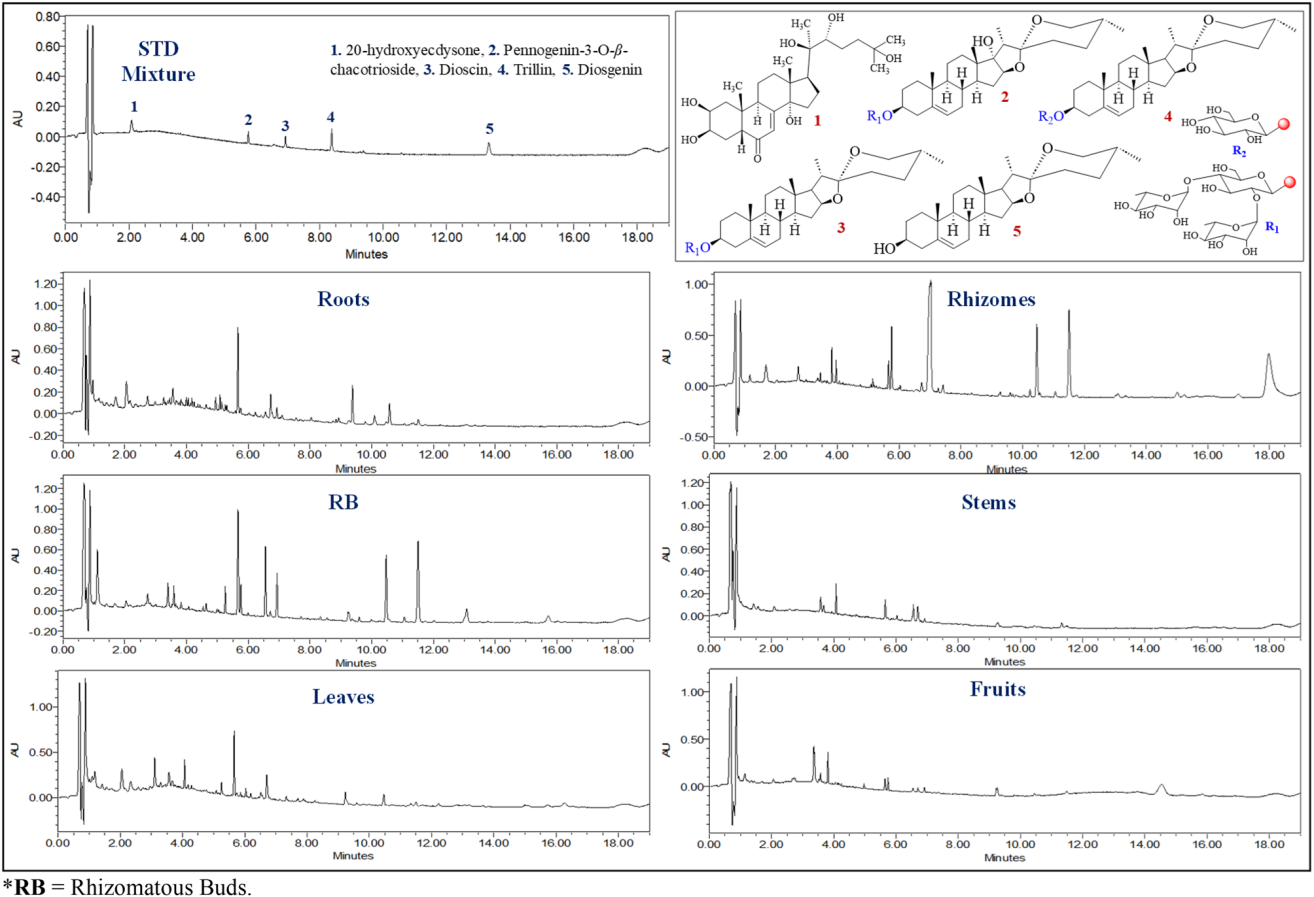
UPLC-PDA chromatogram of standard mixture (Compounds **1–5**) and different organs of *T. govanianum*. *RB = Rhizomatous Buds.

*T. govanianum* is known to contain steroidal saponins in rhizomes which plays an important role in biological activities. The validated UPLC-PDA method was employed for the qualitative and quantitative estimation of 4 steroidal (pennogenin-3-O-*β*-chacotrioside, dioscin, trillin, diosgenin) and one ecdysteroid (20-hydroxyecdysone) compounds in the different parts extract. Representative chromatograms of standard mixture and specific organ samples were shown in Fig. 1**.** The results revealed that 20-hydroxyecdysone was quantified in all organs, whereas pennogenin-3-O-*β*-chacotrioside, dioscin, and diosgenin were abundantly quantified in underground parts. 20-hydroxyecdysone was found highest in roots (26.89 ± 0.80 mg/g) followed by leaves (26.05 ± 0.50 mg/g), whereas extracts of buds, stems, fruit, and rhizomes were found to contain in the range of 3.50–7.11 mg/g. 20-hydroxyecdysone is a phytoecdysteroids exhibited anti-diabetic, anti-inflammatory, hepatoprotective activities^20,21^. Previously, water and ethanol extract of *T. govanianum* was reported to contain 20-hydroxyecdysone^3^. Further, pennogenin-3-*O*-*β*-chacotrioside and dioscin were found prominent in rhizomes (46.05 ± 0.23 mg/g and 338.47 ± 0.90 mg/g) as well as rhizomatous buds (18.08 ± 0.53 mg/g and 32.49 ± 0.65 mg/g), respectively. They were found absent in leaves and stems of *T*. *govanianum*. Diosgenin (a cortico-steroid hormone) was only quantified in rhizomes in very low quantity (1.08 ± 0.06 mg/g) (Table 1). Among all the samples dioscin was present in the highest amount (338.47 ± 0.90 mg/g). Moreover, trillin was found absent in all the samples.

**Table 1:** Quantification of steroidal compounds, free sugars, micro and macro elements in different organs of *T. govanianum*.

	Steroids (mg/g)					
	Roots	Rhizomes	RB	Stem	Leaves	Fruit
Metabolites						
20-hydroxyecdysone	26.89 ± 0.80	3.50 ± 0.13	7.11 ± 0.04	4.21 ± 0.15	26.05 ± 0.50	3.86 ± 0.46
Pennogenin-3-O-*β*-chacotrioside	2.46 ± 0.18	46.05 ± 0.23	18.08 ± 0.53	NQ	ND	7.73 ± 0.55
Dioscin	5.98 ± 0.05	338.47 ± 0.90	32.49 ± 0.65	NQ	ND	2.43 ± 0.13
Trillin	ND	ND	ND	ND	ND	ND
Diosgenin	NQ	1.08 ± 0.06	ND	ND	ND	ND
**Sugars (mg/g)**						
2-deoxy-ribose	76.89 ± 0.99	40.15 ± 0.38	30.89 ± 0.13	88.42 ± 0.92	116.24 ± 1.26	30.9 ± 0.15
Fructose	16.86 ± 0.03	454.76 ± 12.14	45.93 ± 0.46	42.27 ± 2.20	21.03 ± 0.25	338.74 ± 5.94
Glucose	23.63 ± 0.12	19.79 ± 0.07	32.60 ± 0.57	85.47 ± 6.27	ND	243.21 ± 7.53
Galactose	ND	19.03 ± 0.39	ND	ND	ND	69.06 ± 2.14
**Elemental analysis (mg/g)**						
Zn	0.272 ± 0.002	1.629 ± 0.010	1.255 ± 0.023	4.715 ± 0.015	0.107 ± 0.003	0.269 ± 0.002
Cu	0.080 ± 0.027	0.017 ± 0.007	0.039 ± 0.004	0.015 ± 0.004	0.014 ± 0.004	0.035 ± 0.006
Ca	0.256 ± 0.001	0.373 ± 0.002	0.982 ± 0.003	1.715 ± 0.002	2.473 ± 0.020	0.325 ± 0.008
Mn	0.161 ± 0.005	0.005 ± 0.002	0.012 ± 0.005	0.028 ± 0.002	0.035 ± 0.003	0.062 ± 0.003
Mg	6.437 ± 0.019	5.432 ± 0.034	10.191 ± 0.065	6.429 ± 0.044	6.443 ± 0.036	5.983 ± 0.021
Ni	0.011 ± 0.002	0.014 ± 0.003	0.018 ± 0.009	0.007 ± 0.001	0.009 ± 0.003	0.005 ± 0.002
Cr	ND	ND	ND	ND	ND	ND
Cd	ND	ND	ND	ND	ND	ND
K	10.669 ± 0.092	3.000 ± 0.542	11.554 ± 0.247	12.052 ± 0.057	10.211 ± 0.117	3.910 ± 0.749
Pb	ND	ND	ND	ND	ND	ND
Fe	9.929 ± 0.072	0.033 ± 0.001	0.726 ± 0.013	ND	3.480 ± 0.009	3.935 ± 0.047
Na	8.553 ± 0.172	0.113 ± 0.014	ND	5.568 ± 0.147	8.744 ± 0.091	6.076 ± 0.098

*Data shown as mean ± SD, RB = Rhizomatous buds, *NQ = Not Quantified, **ND = Not Detected.

### Free sugars quantification in different parts

Carbohydrates play a crucial role in biochemical and physiological processes, and overall plant functioning, including imparting adaptation abilities. Various sugars have been reported to protect from cold and drought stresses, phosphorus deficiency, and pathogen attack^22^. UPLC-ELSD-based analysis showed clear chromatographic separation of nine sugars (2-deoxy-rhamnose, arabinose, fructose, galactose, glucose, mannose, myoinositol, rhamnose, and trehalose: Fig. S3)**.** Four sugars were quantified among the nine targeted sugars in most of the *T. govanianum* organs*.* 2-deoxyribose and fructose were quantified in all the extracts and highest in leaves (116.24 ± 1.26 mg/g) and rhizomes (454.76 ± 12.14 mg/g) extracts, respectively. Similarly, glucose (243.21 ± 7.53 mg/g) and galactose (69.06 ± 2.14 mg/g) were major in fruits as compared to other parts. Further, rhamnose, arabinose, mannose, myoinositol, and trehalose were not detected in any parts of *T. govanianum* (Table 1).

### Elemental analysis in different parts

Trace elements of plant-based medicines are key components that helps in the treatment of metabolic disorders. Approximately, forty elements have been considered essential for the survival of animals and plants^23^. These elements act as coenzymes in metabolic processes^22^. Eight essential elements viz. Fe (Iron), Mn (Manganese), Ca (Calcium), Mg (Magnesium), Na (Sodium), K (Potassium), Zn (Zinc), and Cu (Copper) were found in the extracts of *T*. *govanianum*. Mg, K, and Fe were found highest in all samples as compared to other elements. Further, Fe was highest in roots (9.929 ± 0.072 mg/g) followed by fruits (3.935 ± 0.047 mg/g), leaves (3.480 ± 0.009 mg/g), rhizomatous buds (0.726 ± 0.013 mg/g), and rhizomes (0.033 ± 0.001 mg/g). Similarly, Mg (10.191 ± 0.065 mg/g) and K (12.052 ± 0.057 mg/g) were reported highest in rhizomatous buds and stems samples, respectively. Zn was found highest in stems (4.715 ± 0.015 mg/g) and rhizomes (1.629 ± 0.010) samples while Na was found in all samples except rhizomatous buds (Table 1). Further, Cu, Ca, Mn, and Ni was found in trace quantities, whereas Cr (Chromium) and toxic heavy elements viz. Pb (Lead), Cd (Cadmium) were found absent in the samples of *T*. *govanianum.*

### Non-targeted metabolites profiling using UHPLC-QTOF-IMS

**Figure 2:**
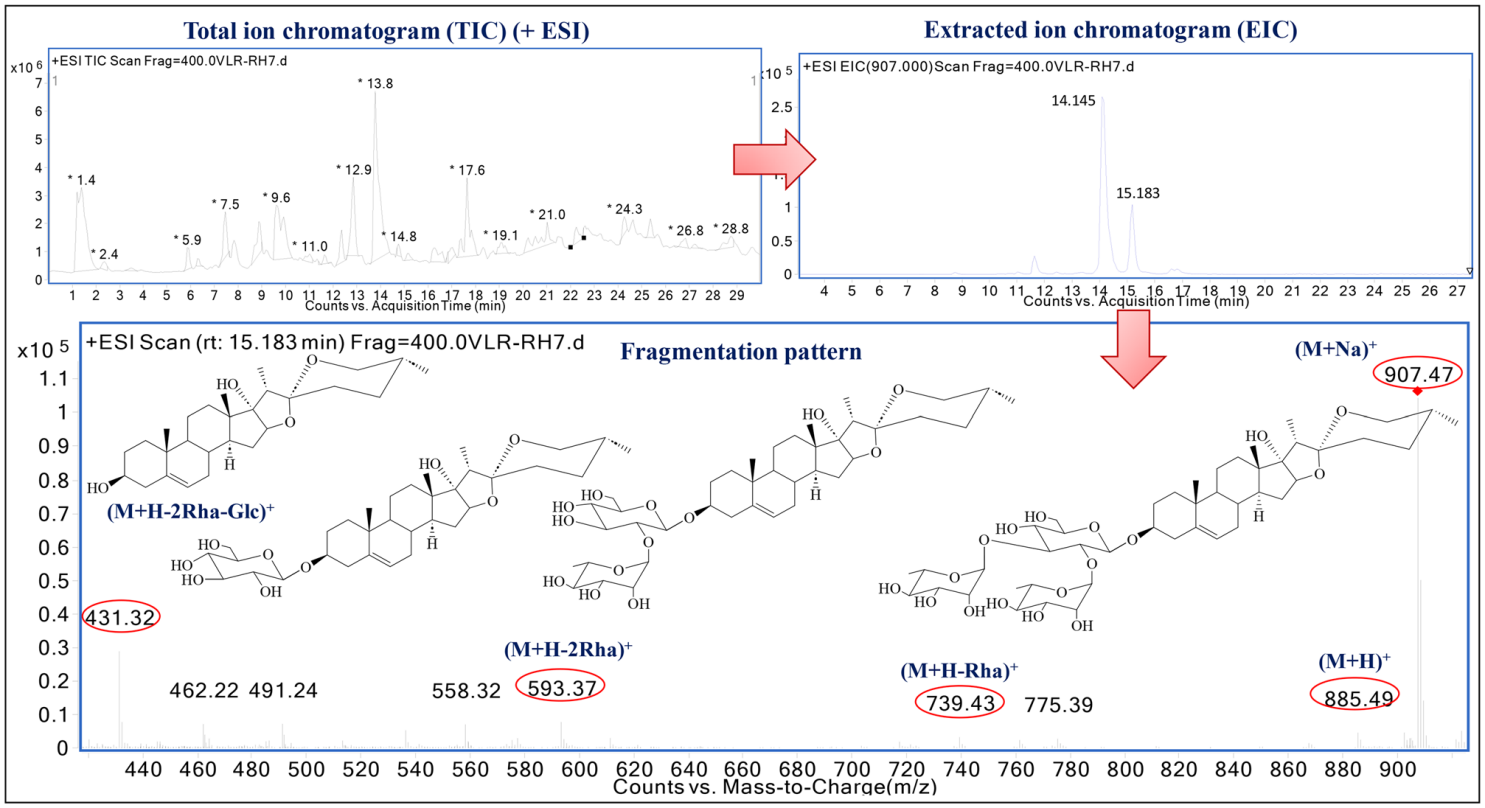
Representative total ion chromatogram (TIC), extracted ion chromatogram (EIC) with steps-wise schematic presentation for compounds identification. *Retention time of peak **73:** 15.183 min and [M+H]^+^: 885.48 m*/z* and represents pennogenin-3-O-*α*-*L*-rhamnopyranosyl-(1 → 2)-*α*-*L*-rhamnopyranosy1-(1 → 3)-*β*-*D*-glucopyranoside.

**Figure 3:**
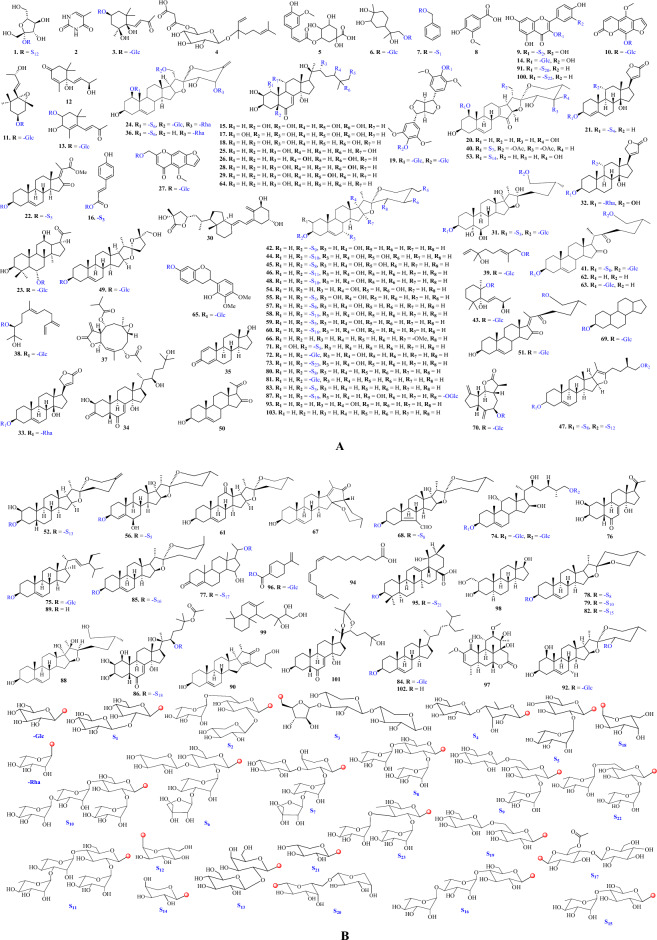
(**A**) Structures of identified compounds. (**B**) Structures of identified compounds with glycosidic linkage.

Metabolites of different parts of *T. govanianum* were profiled using UHPLC-QTOF-IMS. Total ion chromatograms (TIC) of different parts extract were analysed in positive ion mode and peaks were identified as individual metabolites (Fig. S4). The tentative identification of metabolites was assured with retention time, UV–VIS spectra, and mass spectra (precise mass, fragmentation pattern, and isotopic distribution). The exuded positive ion ESI mass spectra were found due to (M+H) ^+^ cations, out of which most of the spectra were due to the losses of sugar moieties. A schematic diagram was presented in Fig. 2 that showed steps of confident identification of pennogenin-3-O-*α*-*L*-rhamnopyranosyl-(1 → 2)-*α*-*L*-rhamnopyranosy1-(1 → 3)-*β*-*D*-glucopyranoside (peak **73**)^24^. The extracted ion chromatograms (ESI-EIC) of its protonated adduct was detected with *m/z* 885.48 (M+H)^+^ at retention time (RT) of 15.183 min. The predicted molecular formula for this adducts was C_45_H_72_O_17_. The sodiated (M+Na)^+^ adduct was detected with *m/z* 907.46, with three intense and very prominent peaks by loss one rhamnose, two rhamnose, two rhamnose + one glucose were detected at *m/z* 739.42 (M+H-Rha)^+^, 593.36 (M+H-2Rha)^+^, 431.31 (M+H-2Rha-Glc)^+^ respectively (Table 2 and Table S2)^24^. A total 103 molecules were identified based on their MS/MS spectral pattern in the samples of *T. govanianum*, which comprises of 6 carbohydrates, 11 terpenoids, 4 polyphenols, 5 flavonoids, 73 steroids and saponins, and 4 other organic compounds. The MS chromatogram, mass fragments, molecular formula briefly discussed in Table 2, S2 and Fig. S5. Further structures of identified compounds were depicted in Fig. 3A, B.

**Table 2:** UHPLC-Q-TOF-IMS-based metabolites profiling in *T. govanianum* organs.

S.N	Rt (min)	Molecular formula	Exact Mass	Observed mass	Observed Mass fragments	Proposed compounds	RH	R	BD	S	L	F	Classification	References
1	1.539	C_12_H_22_O_11_	342.30	365.10 (M+Na)^+^	181.09 (M+H-Glc) ^+^	Sucrose	** + **	** + **	** + **	**−**	** + **	** + **	Carbohydrate & derivatives	^25^
2	1.660	C_5_H_6_N_2_O_2_	126.11	127.05 (M+H)^+^	144.10 (M+H_2_O) ^+^	Thymine	** + **	** + **	** + **	** + **	** + **	**−**	Nucleobase	^3^
3	1.903	C_19_H_32_O_9_	404.5	427.19 (M+Na) +	242.10 (M-Glc) ^+^	Pisumionoside	** + **	** + **	**-**	**-**	** + **	**-**	Terpenoids & derivatives	^29^
4	2.147	C_19_H_30_O_9_	402.4	425.17 (M+Na) ^+^	366.17 (M-2H_2_O) ^+^, 180. 06 (M + 2H_2_O-C_10_H_18_O-C_3_H_4_O_4_)+	*D*-linalool 3-(6''-malonylglucoside)	**-**	**-**	**-**	**-**	** + **	**-**	Carbohydrate & derivatives	PubChem CID-131752917
5	3.074	C_17_H_20_O_9_	368.3	369.11 (M+H)^+^	175.03 (M+H-C_10_H_10_O_4_)^+^	3-*O*-caffeoyl-4-*O*-methylquinic acid	**-**	** + **	**-**	**-**	** + **	**-**	Phenolics	^40^
6	3.563	C_16_H_30_O_9_	366.4	389.17 (M+Na) ^+^	223.13 (M+H+H_2_O-Glc) ^+^, 205.12 (M+H-Glc) ^+^	(1R*,2R*,4R*,8S*)-*p*-Menthane-1,2,8,9-tetrol 9-glucoside	**-**	**-**	**-**	**-**	** + **	**-**	Terpenoids & derivatives	Pubchem CID-85402711
7	4.303	C_19_H_28_O_11_	432.4	455.15 (M+Na) +	271.11 (M+H-Glc) ^+^, 129.99 (M + K-2Glc-H_2_O) ^+^	Zizybeoside I	** + **	** + **	**-**	**-**	** + **	**-**	Carbohydrate & derivatives	^26^
8	4.995	C_8_H_8_O_4_	168.14	169.07 (M+H)^+^	192.07 (M+H + Na) ^+^	Vanillic acid	** + **	** + **	** + **	** + **	** + **	** + **	Phenolics	^41^
9	5.031	C_32_H_38_O_20_	742.6	743.20 (M+H)^+^	765.18 (M+Na) ^+^, 611.16 (M+H-Xyl) ^+^, 267.12 (M+H-Xyl-Rha-Glc-2H_2_O)^+^	Quercetin -3-(2G-xylosylrutinoside)	**-**	**-**	**-**	**-**	** + **	**-**	Flavonoids	^44^
10	5.278	C_18_H_18_O_10_	394.3	395.09 (M+H)^+^	215.12 (M+H-Glc-H_2_O) ^+^	9-hydroxy-4-methoxypsoralen-9-glucoside	**-**	**-**	**-**	**-**	** + **	**-**	Phenolics glycoside	^42^
11	5.524	C_19_H_32_O_8_	388.5	389.21 (M+H)^+^	411.19 (M+Na) ^+^, 227.1649 (M+H-Glc) ^+^	Corchoionoside A	** + **	**-**	** + **	** + **	** + **	** + **	Carbohydrate & derivatives	^27^
12	5.771	C_13_H_20_O_3_	224.3	225.14 (M+H)^+^	243.11 (M+H+H_2_O) ^+^, 207.13 (M+H-H_2_O)^+^, 189.12 (M+H-2H_2_O)^+^	(6*S*,9*R*)-vomifoliol	**-**	** + **	**-**	**-**	** + **	**-**	Terpenoids & derivatives	^30^
13	5.833	C_19_H_30_O_8_	386.4	387.20 (M+H)^+^	409.18 (M+Na)^+^, 225.14 (M+H-Glc)^+^, 207.13 (M+H-Glc-H_2_O)^+^	Sonchuionoside C	** + **	**-**	** + **	**-**	** + **	** + **	Terpenoids & derivatives	^31^
14	5.958	C_21_H_20_O_12_	464.09	464.21 (M)^+^	303.04 (M+H-Glc) ^+^	Quercetin-​3-​O-​*β*-​D-​glucopyranoside	+	+	** + **	** + **	**-**	** + **	Flavonoid	^45^
15	6.284	C_27_H_44_O_9_	512.31	513.30 (M+H)^+^	535.28 (M+Na) ^+^, 495.29 (M+H-H_2_O)^+^, 477.28 (M+H-2H_2_O)^+^, 459.27 (M+H-3H_2_O)^+^	(2*β*,3*β*,5*β*,22*R*)-2,3,5,14,20,22,25,26-octahydroxycholest-7-en-6-one	+	+	** + **	** + **	** + **	** + **	Phytoecdysteroid	^3^
16	6.381	C_24_H_32_O_15_	560.5	583.16 (M+Na)^+^	447.18 (M+H+H_2_O-Xyl) ^+^, 183.09 (M+H+H_2_O-Arf-2Xyl) ^+^	5''-(4-hydroxy-(*E*)-cinnamoyl) alpha-*L*-arabinofuranosyl-(1 → 3)-beta-*D*-xylopyranosyl-(1 → 4)-*D*-xylopyranoside	-	+	**-**	**-**	** + **	** + **	Carbohydrate & derivatives	Pubchem CID-14632958
17	6.406	C_27_H_44_O_9_	512.31	513.30 (M+H)^+^	535.28 (M+Na) ^+^, 495.29 (M+H-H_2_O)^+^, 477.28 (M+H-2H_2_O)^+^,	26-hydroxyintegristerone A	+	+	** + **	** + **	** + **	** + **	Phytoecdysteroid	^72^
18	6.469	C_27_H_44_O_8_	496.31	497.31 (M+H)^+^	519.29 (M+Na) ^+^, 461.29 (M+H-2H_2_O)^+^	5,20-dihydroxyecdysone/ Polypodine B	+	+	+	+	+	+	Phytoecdysteroid	^3^
19	6.745	C_34_H_46_O_18_	742.7	765.25 (M+Na)^+^	617.27 (M+H+2H_2_O-Glc) ^+^, 455.22 (M+H+2H_2_O-2Glc) ^+^	Acanthoside D	-	+	-	-	+	-	Phenolics glycoside (lignan)	^43^
20	6.777	C_26_H_36_O_7_	460.2	461.29 (M+H)^+^	443.27 (M+H-H_2_O)^+^, 425.17 (M+H-2H_2_O)^+^	21-deoxytrillenogenin	+	+	+	+	+	+	Steroid	^82^
21	6.899	C_35_H_52_O_13_	680.8	681.34 (M+H)^+^	519.29 (M+H-Glc)^+^, 355.17 (M+H-Glc-Rha-H2O)^+^	Canarigenin 3-[glucosyl-(1 → 4)-6-deoxy-alloside]	+	+	** + **	** + **	** + **	**-**	cardenolide	^80^
22	6.962	C_35_H_52_O_13_	680.3	681.34 (M+H)^+^	535.28 (M+H-Rha)^+^, 331.13 (M+H+H_2_O-Rha-Glc-CH_3_COOH)^+^	Trillikamtoside M	+	+	+	+	+	+	Furostanol Saponins	^64^
23	7.242	C_30_H_50_O_9_	554.7	555.24 (M+H)^+^	519.29 (M+H-2H_2_O)^+^, 411.19 (M+H+H_2_O-Glc)^+^	Notoginsenoside R10	-	+	-	+	+	-	Steroidal Saponin	^83^
24	7.270	C_61_H_96_O_33_	1379.57 (M+Na) +	1379.57 (M+Na) ^+^	1233.51 (M+Na-Rha)^+^, 1101.47 (M+Na-Api-Rha)^+^, 969.43 (M+Na-Api-Xyl-Rha)^+^, 807.37 (M+Na-Api-Xyl-Rha -Glc)^+^, 621.32 (M+H-Api-Xyl-2Rha-Glc-H_2_O)^+^	Govanoside D	+	+	+	-	-	+	Spirostanol steroidal Saponin	^48^
25	7.329	C_27_H_44_O_7_	480.6	481.31 (M+H)^+^	503.29 (M+Na)^+^, 463.30 (M+H-H_2_O)^+^, 445.29 (M+H-2H_2_O)^+^, 427.28 (M+H-3H_2_O)^+^, 409.18 (M+H-4H_2_O)^+^	Ajugasterone C	+	+	+	+	-	+	Phytoecdysteroid	^73^
26	7.392	C_27_H_44_O_7_	480.30	481.31 (M+H)^+^	503.29 (M+Na)^+^, 463.30 (M+H-H_2_O)^+^, 445.29 (M+H-2H_2_O)^+^, 427.28 (M+H-3H_2_O)^+^	Pinnatasterone	+	+	+	+	+	+	Phytoecdysteroid	^62^
27	7.426	C_19_H_20_O_10_	408.4	409.18 (M+H)^+^	427.28 (M+H+H_2_O)^+^, 265.14 (M+H+H_2_O-Glc)^+^	Khellol glucoside	-	+	-	-	+	-	heterocyclic organic compound	^91^
28	7.454	C_27_H_44_O_7_	480.32	481.31 (M+H)^+^	503.29 (M+Na) ^+^, 463.30 (M+H-H_2_O)^+^, 445.29 (M+H-2H_2_O)^+^, 427.28 (M+H-3H_2_O)^+^, 409.27 (M+H-4H_2_O)^+^	20-hydroxyecdysone	+	+	+	+	+	+	Phytoecdysteroid	^3^
29	7.575	C_27_H_44_O_7_	480.32	481.31 (M+H)^+^	503.29 (M+Na) ^+^, 463.30 (M+H-H_2_O)^+^, 445.29 (M+H-2H_2_O)^+^, 427.28 (M+H-3H_2_O)^+^	Inokosterone	+	+	+	+	+	+	Phytoecdysteroid	^74^
30	7.610	C_27_H_40_O_5_	444.6	445.29 (M+H)^+^	463.30 (M+H+H_2_O) ^+^, 468.25 (M+H + Na) ^+^, 481.31 (M+H+2H_2_O) ^+^, 427.28 (M+H-H_2_O) ^+^	1,25-dihydroxyvitamin D3-26,23-lactone	+	+	+	+	+	+	vitamin D derivative	^92^
31	7.638	C_45_H_76_O_21_	952.48	953.43 (M+H)^+^	807.37 (M+H-Rha) ^+^, 627.37 (M+H-Rha-Glc-H_2_O) ^+^, 519.29 (M+H+2H_2_O-Rha-2Glc) ^+^	Trillfurostanoside H	+	-	-	-	-	-	Furostanol Saponins	^65^
32	7.759	C_29_H_44_O_9_	536.7	537.30 (M+H)^+^	519.29 (M+H-H_2_O) ^+^, 391.24 (M+H-Rha) ^+^, 373.23 (M+H-Rha-H_2_O) ^+^	Rhodexin A	+	+	+	-	-	+	cardenolide	^79^
33	7.822	C_29_H_42_O8	518.6	519.29 (M+H)^+^	391.24 (M+H+H_2_O -Rha)^+^, 373.23 (M+H-Rha)^+^	Corchoriside B	+	+	-	+	-	+	Cardenolide	^81^
34	7.856	C_27_H_42_O_6_	462.6	463.30 (M+H)^+^	481.31 (M+H+H_2_O) ^+^, 445.29 (M+H-H_2_O) ^+^, 427.28 (M+H-2H_2_O)^+^	3-dehydroecdysone	-	+	-	-	+	-	Phytoecdysteroid	^75^
35	7.885	C_19_H_26_O_2_	286.4	287.20 (M+H)^+^	269.19 (M+H-H_2_O) ^+^	Dehydrotestosterone/ Boldenone	+	+	+	-	+	+	Steroid	^84^
36	7.943	C_55_H_86_O_28_	1194.5	1217.52 (M+Na)^+^	1085.47 (M+Na-Api)^+^,939.42 (M+Na-Api-Rha) ^+^, 807.37 (M+Na-Api-Rha-Xyl) ^+^, 620.25 (M-Api-2Rha-Xyl-H_2_O) ^+^	Govanoside B	+	+	+	-	-	+	Spirostanol steroidal Saponin	^49^
37	8.006	C_21_H_28_O_7_	392.4	393.18 (M+H)^+^	357.18 (M+H-2H_2_O) ^+^, 287.20 (M+H-H_2_O-C_4_H_8_O_2_) ^+^	Viguiestenin	+	+	+	+	+	+	Terpenoids & derivatives	^32^
38	8.437	C_21_H_36_O_7_	400.24	401.19 (M+H)^+^	239.08 (M+H-Glc) ^+^	7,​11-​dimethyl-​3-​methylene-​1,​6-​dodecadien-​10,​11-​dihydroxyl-​10-​*O*-​*β*-*​D*-​glucopyranoside	+	+	+	+	+	+	Terpenoids & derivatives	^33^
39	8.595	C_16_H_28_O_7_	332.39	333.16 (M+H)^+^	355.17 (M+Na) ^+^, 153.09 (M+H-Glc-H_2_O) ^+^	Betulalbuside A	-	-	-	-	+	-	Carbohydrate & derivatives	^28^
40	8.618	C_51_H_74_O_26_	1102.4	1103.52 (M+H)^+^	693.28 (M+H-xyl-api-Rha) ^+^, 543.24 (M+H-xyl-api-Rha-ara-H_2_O) ^+^	Trilliumoside L	+	-	-	-	-	-	Spirostanol steroidal Saponin	^50^
41	8.676	C_51_H_82_O_22_	1046.5	1047.53 (M+H)^+^	901.47 (M+H-Rha)^+^, 755.42 (M+H-2Rha)^+^, 593.36 (M+H-2Rha-Glc)^+^, 413.30 (M+H-2Rha-2Glc-H_2_O)^+^	Anguivioside XV	+	-	+	-	-	+	Furostanol Saponins	^65^
42	8.739	C_45_H_72_O_18_	900.4	901.47 (M+H)^+^	755.42 (M+H-Rha)^+^, 593.36 (M+H-Rha-Glc)^+^, 431.31 (M+H-Rha-2Glc)^+^, 413.30 (M+H-Rha-2Glc-H_2_O)^+^	Pennogenin-3-*O*-*β*-*D*-glucopyranosyl-(1 → 6)-[*O*-*α*-*L*-rhamnopyranosyl- (1 → 2)]-*O*-*β*-*D*-glucopyranoside (Trikamsteroside B)	+	-	+	-	-	+	Spirostanol steroidal Saponin	^51^
43	8.779	C_19_H_34_O_8_	390.5	391.17 (M+H)^+^	413.21 (M+Na) ^+^, 229.14 (M+H-Glc) ^+^	Rehmaionoside B	-	-	-	-	+	-	Terpenoids & derivatives	^34^
44	8.802	C_51_H_82_O_22_	1046.5	1047.53 (M+H)^+^	901.47 (M+H-Rha)^+^, 755.42 (M+H-2Rha)^+^, 609.24 (M+H-3Rha)^+^	Polyphylloside III	+	-	+	-	-	+	Spirostanol steroidal Saponin	^52^
45	8.865	C_45_H_72_O_18_	900.47	901.47 (M+H)^+^	755.42 (M+H-Rha)^+^, 609.24 (M+H-2Rha)^+^	(25*S*)​-​27-​hydroxypennogenin-​3*β*-​*O*-​*α*-​*L*-​rhamnopyranosyl-​(1 → 4)​-​*O*-​*α*-​*L*-​rhamnopyranosyl-​(1 → 2)​]​-​*O*-​*β*-​*D*-​glucopyranoside	+	-	+	-	-	+	Spirostanol steroidal Saponin	^53^
46	9.542	C_51_H_82_O_21_	1030.5	1031.54 (M+H)^+^	885.48 (M+H-Rha)^+^, 413.31 (M+H-3Rha-Glc-H_2_O)^+^	Pennogenin-3-*O*-rhamnosyl-*β*-chacotrioside	+	+	-	+	-	+	Spirostanol steroidal Saponin	^54^
47	9.646	C_51_H_82_O_21_	1030.5	1031.54 (M+H)^+^	577.37 (M+H-2Rha-Glc)^+^, 415.32 (M+H-2Rha-2Glc)^+^	Pseudoprotodioscin	+	+	+	-	-	+	Furostanol Saponins	^66^
48	10.094	C_51_H_82_O_21_	1030.53	1031.54 (M+H)^+^	885.48 (M+H-Rha)^+^, 739. 42 (M+H-2Rha)^+^, 431.31 (M+H-3Rha-Glc)^+^	Pennogenin tetraglycoside	+	-	-	-	-	-	Spirostanol steroidal Saponin	^49^
49	10.157	C_33_H_52_O_9_	592.8	593.36 (M+H)^+^	431.31 (M+H-Glc)^+^, 413.30 (M+H-Glc-H_2_O)^+^	Nuatigenin-3-*β*-D-glucopyranoside	+	-	-	-	-	-	Spirostanol steroidal Saponin	^55^
50	10.521	C_21_H_30_O_3_	330.5	331.21 (M+H)^+^	313.16 (M+H-H_2_O)^+^	3*-*hydroxypregnan-5-ene-16,20-dione	+	+	-	-	-	-	Steroid	^85^
51	10.947	C_33_H_50_O_9_	590.34	591.35 (M+H)^+^	429.30 (M+H-Glc)^+^, 411.28 (M+H-Glc-H_2_O)^+^	Trilliumoside	+	-	-	+	+	-	Furostanol Saponins	^63^
52	11.135	C_39_H_62_O_14_	754.9	755.42 (M+H)^+^	777.40 (M+H + Na)^+^, 593.37 (M+H-Glc)^+^, 413.30 (M+H-2Glc-H_2_O)^+^	Schidigerasaponin C2	+	-	-	+	-	-	Spirostanol steroidal Saponin	^56^
53	11.256	C_31_H_44_O_11_	592.2	593.36 (M+H)^+^	461.37 (M+H-Ara)^+^	21-deoxytrillenogenin-1-*O*-*α*-*L*- arabinopyranoside	+	-	-	-	-	-	Spirostanol steroidal Saponin	^57^
54	11.319	C_27_H_42_O_5_	446.30	447.30 (M+H)^+^	429.30 (M+H-H_2_O)^+^, 411.29 (M+H-2H_2_O)^+^	24-hydroxypennogenin	+	+	+	+	-	-	Steroid	^3^
55	11.382	C_39_H_62_O_14_	754.4	755.42 (M+H)^+^	591.35 (M+H-Rha-H_2_O)^+^, 447.30 (M+H-Rha-Glc)^+^	Trillikamtoside F	+	-	-	+	-	-	Spirostanol steroidal Saponin	^58^
56	11.440	C_39_H_62_O_14_	754.4	755.42 (M+H)^+^	627.31 (M+H+H_2_O-Rha)^+^, 447.29 (M+H-Rha-Glc)^+^, 429.30 (M+H-Rha-Glc-H_2_O)^+^, 411.29 (M+H-Rha-Glc-2H_2_O)^+^	Trillikamtoside A	+	-	-	+	-	-	Spirostanol steroidal Saponin	^58^
57	11.566	C_45_H_72_O_17_	884.47	885.48 (M+H)^+^	907.46 (M+Na)^+^, 739. 42 (M+H-Rha)^+^, 593.36 (M+H-2Rha)^+^, 431.31 (M+H-2Rha-Glc)^+^, 413.30 (M+H-2Rha-Glc-H_2_O)^+^	Pennogenin triglycoside	+	-	-	+	-	-	Spirostanol steroidal Saponin	^49^
58	11.628	C_39_H_62_O_13_	738.41	739.42 (M+H)^+^	593.36 (M+H-Rha)^+^, 431.31 (M+H-Rha-Glc)^+^ 413.30 ((M+H-Rha-Glc-H_2_O)^+^	Pennogenin-3-O-*α*-*L*-rhamnopyranosyl-(1 → 4)-*β*-*D*-glucopyranoside	+	-	-	+	-	-	Spirostanol steroidal Saponin	^59^
59	11.687	C_39_H_62_O_13_	738.41	739.42 (M+H)^+^	593.36 (M+H-Rha)^+^, 431.31 (M+H-Rha-Glc)^+^, 413.30 (M+H-Rha-Glc-H_2_O)^+^	Pennogenin-3-O-*α*-*L*-rhamnopyranosyl-(1 → 2)-*β*-*D*-glucopyranoside (Polyphyllin VI)	+	-	-	+	-	-	Spirostanol steroidal Saponin	^50^
60	11.746	C_45_H_72_O_17_	884.4	885.48 (M+H)^+^	907.46 (M+Na)^+^, 739. 42 (M+H-Rha)^+^, 593.36 (M+H-2Rha)^+^, 431.31 (M+H-2Rha-Glc)^+^, 413.30 (M+H-2Rha-Glc-H_2_O)^+^	Pennogenin-3-O-*α*-*L*-rhamnopyranosyl-(1 → 4)-*α*-*L*-rhamnopyranosy1-(1 → 4)-*β*-*D*-glucopyranoside	+	-	-	+	-	-	Spirostanol steroidal Saponin	^59^
61	13.097	C_27_H_40_O_4_	428.6	429.30 (M+H)^+^	411.29 (M+H-H_2_O)^+^, 393.28 (M+H-2H_2_O)^+^	Gentrogenin	+	-	-	+	+	+	Steroid	^86^
62	13.343	C_27_H_42_O_4_	430.3	431.31 (M+H)^+^	413.30 (M+H-H_2_O) ^+^, 395.29 (M+H-2H_2_O)^+^	Kryptogenin	+	+	+	+	+	+	Steroid	^87^
63	14.145	C_33_H_52_O_9_	592.3	593.36 (M+H)^+^	431.31 (M+H-Glc)^+^, 413.30 (M+H-Glc-H_2_O)^+^, 395.29 (M+H-Glc-2H_2_O)^+^	Kryptogenin-3-*O*-*β*-​*D*-​glucopyranoside	+	+	-	-	-	+	Furostanol Saponins	^67^
64	14.472	C_27_H_44_O_6_	464.09	464.35 (M)^+^	446.34 (M+H-H_2_O)^+^, 428.33 (M+H-2H_2_O)^+^, 410.32 (M+H-3H_2_O)^+^	Ponasterone A	+	-	+	+	-	-	Phytoecdysteroid	^74^
65	14.531	C_23_H_28_O_10_	464.4	464.35 (M)^+^	302.30 (M-Glc)^+^, 284.29 (M-Glc-H_2_O)^+^	Isomucronulatol-7-*O*-*β*-*D*-glucoside	+	-	+	+	-	-	Flavonoid	^46^
66	14.631	C_28_H_44_O_4_	444.32	445.29 (M+H)^+^	413.30 (M+H-MeOH)^+^, 395.29 (M+H-MeOH -H_2_O)^+^	Bethogenin	+	-	+	-	-	-	Steroid	^88^
67	14.690	C_27_H_38_O_3_	410.3	411.29 (M+H)^+^	393.27 (M+H-H_2_O)^+^	16,​23-​cyclocholesta-​5,​17(20)​-​dien-​22-​one	+	+	-	+	-	+	Steroid	^88^
68	14.753	C_45_H_70_O_18_	898.45	899.46 (M+H)^+^	607.34 (M+H-2Rha)^+^, 445.29 (M+H-2Rha-Glc)^+^	Trillikamtoside E	+	-	-	-	-	-	Spirostanol steroidal Saponin	^58^
69	14.815	C_23_H_38_O_6_	410.5	411.28 (M+H)^+^	393.27 (M+H-H_2_O)^+^, 249.15 (M+H-Glc)^+^	Sterol-3-beta-*D*-glucoside	+	-	-	-	-	+	Saponin	^89^
70	14.874	C_21_H_30_O_8_	410.5	411.28 (M+H)^+^	393.27 (M+H-H_2_O)^+^, 249.15 (M+H-Glc)^+^	Scorzoside	+	-	-	-	-	+	Terpenoids & derivatives	^35^
71	15.062	C_39_H_62_O_13_	738.9	739.42 (M+H)^+^	761.40 (M+Na)^+^, 593.36 (M+H-Rha)^+^, 431.31 (M+H-Rha-Glc)^+^, 413.30 (M+H-Rha-Glc-H_2_O)^+^	Fistuloside A	+	-	-	-	-	-	Spirostanol steroidal Saponin	^60^
72	15.120	C_33_H_52_O_9_	592.36	593.37 (M+H)^+^	611.32 (M+H+H_2_O)^+^, 431.31 (M+H-Glc)^+^, 413.30 (M+H-Glc-H_2_O)^+^	Pennogenin-3-*O*-*β*-*D*-glucopyranoside	+	-	-	-	-	-	Spirostanol steroidal Saponin	^51^
73	15.183	C_45_H_72_O_17_	884.4	885.48 (M+H)^+^	907.46 (M+Na)^+^, 739. 42 (M+H-Rha)^+^, 593.36 (M+H-2Rha)^+^, 431.31 (M+H-2Rha-Glc)^+^	Pennogenin 3-*O*-*α*-*L*-rhamnopyranosyl-(1 → 2)-*α*-*L*-rhamnopyranosy1-(1 → 3)-*β* -*D*-glucopyranoside	+	-	-	-	-	-	Spirostanol steroidal Saponin	^24^
74	15.287	C_39_H_66_O_15_	774.9	775.38 (M+H)^+^	757.37 (M+H-H_2_O)^+^, 451.2653 (M+H-2Glc)^+^	Heloside A	+	-	-	-	-	-	Furostanol Saponins	^68^
75	15.496	C_35_H_58_O_6_	574.4	575.35 (M+H)^+^	413.30 (M+H-Glc)^+^, 395.29 (M+H-Glc-H_2_O)^+^	Stigmasterol-​3-​O-*​β*-​*D*-​glucopyranoside	+	-	+	-	+	-	Furostanol Saponins	^69^
76	15.659	C_21_H_30_O_5_	362.5	362.32 (M)^+^	344.31 (M-H_2_O)^+^, 327.29 (M+H-2H_2_O)^+^, 308.29 (M-3H_2_O)^+^	Poststerone	+	+	+	-	-	+	Phytoecdysteroid	^76^
77	16.106	C_35_H_54_O_14_	698.8	699.35 (M+H)^+^	483.24 (M+H-Glc-3H_2_O)^+^, 333.16 (M+H-2Glc-CH_3_COOH+H_2_O)^+^	(15a,20*R*)-dihydroxypregn-4-en-3-one 20-[glucosyl-(1 → 4)-6-acetyl-glucoside]	+	-	+	-	-	+	Furostanol Saponins	Pub Chem CID- 85,220,526
78	16.599	C_45_H_72_O_16_	868.4	869.49 (M+H)^+^	891.47 (M+Na)^+^, 723.43 (M+H-Rha)^+^, 577.37 (M+H-2Rha)^+^, 415.32 (M+H-2Rha-Glc)^+^	Borassoside E	+	-	+	+	-	+	Spirostanol steroidal Saponin	^3^
79	16.662	C_51_H_82_O_20_	1014.3	1015.54 (M+H)^+^	869.49 (M+H-Rha)^+^, 723.43 (M+H-2Rha)^+^, 577.37 (M+H-3Rha)^+^, 415.32 (M+H-3Rha-Glc)^+^	Diosgenin-​3-​*O*-*α*-​*L*-​rhamnopyranosyl-​(1 → 4)​-​*α*-​*L*-​rhamnopyranosyl-​(1 → 4)​-​[*α*-*L*-​rhamnopyranosyl (1 → 2)​]​-*​β*-​*D*-​glucopyranoside (Borassoside F)	+	-	-	-	-	-	Spirostanol steroidal Saponin	^61^
80	16.721	C_45_H_72_O_16_	868.49	869.49 (M+H)^+^	891.47 (M+Na)^+^, 723.43 (M+H-Rha)^+^, 577.37 (M+H-2Rha)^+^, 415.32 (M+H-2Rha-Glc)^+^	Dioscin/ Polyphyllin III	+	+	+	-	-	+	Spirostanol steroidal Saponin	^62,63^
81	16.779	C_33_H_52_O_8_	576.8	577.37 (M+H)^+^	559.36 (M+H-H_2_O)^+^, 415.32 (M+H-Glc)^+^	Trillin	+	+	+	-	-	+	Spirostanol steroidal Saponin	^3^
82	16.838	C_39_H_62_O_12_	722.42	723.43 (M+H)^+^	577.37 (M+H-Rha)^+^, 415.32 (M+H-Rha-Glc)^+^	Borassoside D	+	-	+	-	-	-	Spirostanol steroidal Saponin	^61^
83	16.901	C_39_H_62_O_12_	722.4	723.43 (M+H)^+^	577.37 (M+H-Rha)^+^, 415.32 (M+H-Rha-Glc)^+^, 397.31 (M+H-Rha-Glc-H_2_O)^+^	Polyphyllin V	+	-	+	-	-	-	Spirostanol steroidal Saponin	^3^
84	17.148	C_35_H_60_O_6_	576.4	577.37 (M+H)^+^	415.32 (M+H-Glc)^+^, 397.31 (M+H-Glc-H_2_O)^+^	Daucosterol	+	-	-	-	-	-	Furostanol Saponins	^70^
85	17.207	C_45_H_72_O_16_	868.49	869.48 (M+H)^+^	891.47 (M+Na)^+^, 577.37 (M+H-2Rha)^+^, 415.32 (M+H-2Rha-Glc)^+^	Borassoside A	+	-	-	-	-	-	Spirostanol steroidal Saponin	^61^
86	17.390	C_35_H_56_O_14_	700.39	701.37 (M+H)^+^	723.43 (M+Na)^+^, 520.34 (M-Glc-H_2_O)^+^	Sileneoside H	+	+	+	-	-	+	Phytoecdysteroid	^77^
87	18.255	C_45_H_72_O_20_	932.45	933.48 (M+H)^+^	627.33 (M+H+H_2_O-2Glc)^+^, 446.32 (M-3Glc)^+^, 428.31 (M-3Glc-H_2_O)^+^	Chonglouside SL-18	+	-	-	-	-	-	Spirostanol steroidal Saponin	^51^
88	19.356	C_27_H_44_O_5_	448.31	449.28 (M+H)^+^	471.30 (M+Na)^+^, 430.32 (M-H_2_O)^+^, 413.26 (M+H-2H_2_O)^+^	Nologenin	+	-	+	+	+	+	Furostanol Saponins	^67^
89	19.602	C_29_H_48_O	412.7	413.26 (M+H)^+^	431.31 (M+H+H_2_O)^+^, 395.27 (M+H-H_2_O)^+^	Stigmasterol	+	-	+	-	-	-	Steroid	^90^
90	19.665	C_27_H_40_O_3_	412.6	413.26 (M+H)^+^	395.27 (M+H-H_2_O)^+^, 377.23 (M+H-2H_2_O)^+^	Fesogenin	+	-	+	-	-	-	Steroid	^85^
91	19.787	C_26_H_28_O_15_	580.14	581.36 (M+H)^+^	449.28 (M+H-Ara)^+^, 304.30 (M+H_2_O-Ara-Glactose)^+^	kaempferol-3-*O*-*α-*arabinopyranosyl-(1 → 6)-*β*-galactopyranoside	+	-	-	+	+	+	Flavonoid	^45^
92	19.971	C_33_H_52_O_10_	608.35	609.36 (M+H)^+^	429.31 (M+H-Glc-H_2_O)^+^	Trillikamtoside H	+	-	-	-	-	-	Spirostanol steroidal Saponin	^58^
93	20.095	C_27_H_42_O_4_	430.32	431.31 (M+H)^+^	413.26 (M+H-H_2_O)^+^, 395.27 (M+H-2H_2_O)^+^	Pennogenin	+	+	-	-	-	-	Steroid	^3^
94	20.154	C_18_H_30_O_2_	278.43	279.23 (M+H)^+^	297.24 (M+H+H_2_O)^+^, 301.14 (M+Na)^+^, 243.13 (M+H-2H_2_O)^+^	*α*-linolenic Acid	+	+	+	+	-	+	Fatty acid	^93^
95	20.647	C_35_H_56_O_8_	604.39	605.42 (M+H)^+^	623.37 (M+H+H_2_O)^+^, 473.34 (M+H-Xyl)^+^	Prosapogenin	+	-	+	-	-	-	Triterpenoid Saponin	^33^
96	21.018	C_16_H_24_O_7_	328.36	329.16 (M+H)^+^	351.25 (M+Na)^+^, 122.09 (M+H-Glc-COOH)^+^	Perilloside B	+	+	+	-	-	+	Terpenoids & derivatives	^36^
97	21.140	C_22_H_32_O_8_	424.5	425.21 (M+H)^+^	448.29 (M+H + Na)^+^, 407.27 (M+H-H_2_O)^+^	Nigakilactone H	+	-	-	+	+	+	Terpenoids & derivatives	^37,38^
98	22.645	C_20_H_34_O_3_	322.5	323.25 (M+H)^+^	345.24 (M+Na)^+^, 304.29 (M-H_2_O)^+^	2-*α*-(hydroxymethyl)-5-*α*-androstane-3*β*,17*β*-diol	+	+	+	+	+	-	Steroid	Pubchem CID-244947
99	23.653	C_20_H_36_O_3_	324.5	325.27 (M+H)^+^	347.25 (M+Na)^+^, 307.26 (M+H-H_2_O)^+^	(13*R*,14*R*)-7-labdene-13,14,15-triol	+	-	-	+	+	+	Terpenoids & derivatives	^39^
100	24.698	C_33_H_40_O_19_	740.2	740.54 (M)^+^	430.33 (M-2Rha-H_2_O) , 304.30 (M+H_2_O-2Rha-Glc)^+^	kaempferol 3-*O*-*α-*rhamnosyl-(l → 2)-*O*-[*α*-rhamnosyl-(1 → 6)]-*β*-glucoside / Clitorin	+	-	-	-	-	-	Flavonoids	^45^
101	25.331	C_30_H_48_O_6_	504.7	505.39 (M+H)^+^	487.39 (M+H-H_2_O)^+^, 450.56 (M-3H_2_O)^+^	2-deoxy-20-hydroxyecdysone-20,22-acetonide	+	-	-	-	-	-	Phytoecdysteroid	^74^
102	27.336	C_29_H_50_O	414.7	415.32 (M+H)^+^	397.32 (M+H-H_2_O)^+^	*β*-sitosterol	+	+	-	-	-	-	Steroid	^90^
103	27.462	C_27_H_42_O_3_	414.32	415.32 (M+H)^+^	397.32 (M+H-H_2_O)^+^	Diosgenin	+	+	-	-	-	-	Steroid	^3^

### Carbohydrates and their derivatives

A total of six carbohydrates and their derivatives were identified in *T. govanianum* extracts. Peaks **1, 4, 7, 11, 16, 39** were noticed as a glycoside and derivatives, which were due to the elimination of glucose residue (162 amu), arabinose (132 amu), and neutral losses of H_2_O (18 amu)^25–28^. Peak **1** showed [M+Na]^+^ ion at *m*/*z* 365.10, and its fragment [M+H-Glu]^+^ at *m*/*z* 181.09. This gave the information of peak **1** as sucrose which was identified in all the parts except stem sample of *T. govanianum*^25^. Further, peaks **7** and **11** were identified as zizybeoside I [*m/z* 455.15 (M+Na)^+^] and corchoionoside A [*m/z* 389.21 (M+H)^+^], respectively, which were *o*-glycosyl compound (Table 2 and Table S2)^26,27^. Similarly, all other glycoside (peaks **4**, **16**, **39**) with their fragmentation pattern and MS spectra were shown in Table 2 and Table S2^28^.

### Terpenoids and derivatives

Total eleven terpenoids and their derivatives including monoterpene, diterpenes, sesquiterpene, triterpene and their glycosides (peaks **3**, **6**, **12**, **13**, **37**, **38**, **43**, **70**, **96**, **97**, **99**) were identified and most of these showed (M+H)^+^ and (M+Na)^+^ as molecular ion peaks and their fragments were mainly due to the loses of glucose moiety. Peaks **3** and **6** showed their molecular ion at *m/z* 427.19 and 389.17, respectively, and were due to (M+Na)^+^ ion. Further, their fragmentation pattern showed *m/z* 242.1 (M-Glc)^+^ and *m/z* 223.13 (M+H+H_2_O-Glc)^+^, *m/z* 205.12 (M+H-Glc)^+^, respectively for peak **3** and **6**, which gave the identification as pisumionoside (peak **3**) and (1R*,2R*,4R*,8S*)-*p*-menthane-1,2,8,9-tetrol 9-glucoside (peak **6**), respectively^29^. Similarly, fragmentation pattern leads to the identification of other molecules as peak **12**: (6*S*,9*R*)-vomifoliol (*m/z* 225.14)^30^, peak **13**: sonchuionoside C (*m/z* 387.20)^31^, peak **37**: viguiestenin (*m/z* 393.18)^32^, peak **38**: 7,11-dimethyl-3-methylene-1,6-dodecadien-10,11-dihydroxyl-10-*O*-*β*-*D*-glucopyranoside (*m/z* 401.19)^33^, peak **43**: rehmaionoside B (*m/z* 391.17)^34^, peak **70**: scorzoside (*m/z* 411.28)^35^, peak **96**: perilloside B (*m/z* 329.16)^36^, peak **97**: nigakilactone H (*m/z* 425.21)^37,38^, and peak **99**: (13*R*,14*R*)-7-labdene-13,14,15-triol (*m/z* 325.27), respectively (Table 2 and Table S2)^39^.

### Phenolics and flavonoids

*T. govanianum* extracts revealed the identification of four phenolics and five flavonoids compounds. Four polyphenols were identified as 3-*O*-caffeoyl-4-*O*-methylquinic acid (peak **5**), vanillic acid (peak **8**), 9-hydroxy-4-methoxypsoralen- 9-glucoside (peak **10**), and acanthoside D (peak **19**)^40–43^. In MS analysis, peaks **5**, **8**, **10** showed *m/z* at 369.11, 169.07, 395.09 due to (M+H)^+^ molecular ions. Peak **19** showed *m/z* 765.25 (M+Na)^+^ with its fragments 617.27 (M+H+2H_2_O-Glc) ^+^, 455.22 (M+H+2H_2_O-2Glc) ^+^, due to the loss of glucose moiety and was identified as lignan glycoside (acanthoside D)^43^. Similarly, five flavonoids namely quercetin- 3-(2G-xylosylrutinoside) **(**peak **9)**, quercetin-​3-​*O*-​*β*-​*D*-​glucopyranoside** (**peak **14)**, isomucronulatol-7-*O*-*β*-*D*-glucoside** (**peak **65)**, kaempferol-3-*O*-*α-*arabinopyranosyl-(1 → 6)-*β*-galactopyranoside** (**peak **91)**, kaempferol-3-*O*-*α-*rhamnosyl-(l → 2)-*O*-[*α*-rhamnosyl-(1 → 6)]-*β*-glucoside/clitorin** (**peak **100)** were identified in the extracts of *T. govanianum* and showed molecular ion peaks at *m/z* 743.20, 464.21, 464.35, 581.36, and 740.54, respectively^44–46^. Further, MS spectra’s showed fragmentation pattern with the elimination of glucose/galactose residue (162 amu), arabinose/xylose (132 amu), rhamnose (146 amu), and neutral losses of H_2_O (18 amu) (Table 2**, **Fig. 3A, B and Table S2). Peak **91** showed molecular ion peak (M+H) ^+^ with *m/z* 581.36 at retention time 19.787 min, while their corresponding aglycones were observed at *m*/*z* 449.28 and 304.30, due to the fragments (M+H-Ara) ^+^ and (M+H_2_O-Ara-Glactose) ^+^, respectively. These fragmentation pattern leads to the identification of kaempferol-3-O-*α-*arabinopyranosyl-(1 → 6)-*β*-galactopyranoside (peak **91**)^45^. Similarly, other flavonoids were identified and depicted in Table 2 and Table S2.

### Steroids and saponins

Saponins are secondary plant metabolites and distributed in several species, comprised of steroids, triterpenoids with single or multiple sugar residues^47^. *Trillium* species are rich source of steroids and steroidal saponins. Total 73 steroidal compounds have been identified in extracts of different parts of *T. govanianum*, that comprised of spirostanol & furostanol saponins, cardenolide glycosides, triterpenoid saponin, steroids (phytoecdysteroids) and their glycosides.

### Spirostanol saponins

Spirostanol are the steroidal compounds in which two ring are fused together through a common carbon at C-22 and make a spirane arrangement, while furostanol has open ring system at C-22 position. Thirty compounds (peaks **24**, **36**, **40**, **42**, **44–46**, **48**, **49**, **52**, **53**, **55–60**, **68**, **71–73**, **78–83**, **85**, **87**, **92**) were identified as spirostanol saponins and their mass spectrum showed neutral losses and gains of H_2_O (18 amu) as well as the elimination of glycone residue [glucose/galactose (162 amu), arabinose/xylose/apiose (132 amu), rhamnose (146 amu)]^3,24,48–63^. Peak **24** had [M+Na] ^+^ ion with *m*/*z* 1379.57 and its fragments in MS spectra were *m*/*z* 1233.5156 (M+Na-Rha)^+^, 1101.4736 (M+Na-Api-Rha)^+^, 969.4309 (M+Na-Api-Xyl-Rha)^+^, 807.3783 (M+Na-Api-Xyl-Rha-Glc)^+^, 621.3275 (M+H-Api-Xyl-2Rha-Glc-H_2_O)^+^. This information identified the peak **24** as govanoside D^48^. Similarly, 30 spirostanol saponins was identified based upon the fragmentation pattern, retention time, and literature reports and also depicted in Table 2 and Table S2, Fig. S5.

### Furostanol saponins

Eleven furostanol saponins were identified in samples of *T. govanianum*. Peaks **22**, **31**, **41**, **47**, **51**, **63**, **74**, **75**, **77**, **84**, and **88** were found with their molecular ion (M+H) ^+^ at *m/z* 681.34, 953.43, 1047.53, 1031.54, 591.35, 593.36, 775.38, 575.35, 699.35, 577.37, and 449.28, respectively. MS fragments of these peaks were mostly due to elimination of glucose (162 amu), rhamnose (146 amu), and loss/gain of H_2_O (18 amu) molecule. MS fragmentation tentatively identified the molecules as trillikamtoside M (peak **22**)^64^, trillfurostanoside H (peak **31**)^65^, anguivioside XV (peak **41**)^65^, pseudoprotodioscin (peak **47**)^66^, trilliumoside (peak **51**)^63^, kryptogenin-3-O-*β*-D-glucopyranoside (peak **63**)^67^, heloside A (peak **74**)^68^, stigmasterol-3-O-*β*-D-glucopyranoside (peak **75**)^69^, (15a,20*R*)-dihydroxypregn-4-en-3-one 20-[glucosyl-(1 → 4)-6-acetyl-glucoside] (peak **77**) (Pub Chem CID- 85220526), daucosterol (peak **84**)^70^, and nologenin (peak **88**)^67^. Further, the presence and absence of all these molecules were also marked in the samples (Table 2).

### Phytoecdysteroids

Phytoecdysteroids are polyhydroxylated steroids produced by the plants^71^. UPLC-MS/MS analysis showed the presence of eleven phytoecdysteroids and one phytoecdysteroid glycoside in the samples of *T. govanianum*. Peaks **15**, **17**, **18**, **25**, **26**, **28**, **29**, **34**, **64**, **76**, **86**, **101** were identified as phytoecdysteroids^3,62,72–77^. MS fragmentation pattern of these peaks showed the elimination of multiple H_2_O molecules (18 amu). While, peak **86** showed (M+H) ^+^ ion with *m/z* 701.37 and fragment with *m/z* 520.34 (M-Glc-H_2_O) ^+^. Thus, the molecule is identified as sileneoside H (peak **86**)^77^. All other phyto steroids were identified in same manner (Table 2 and Table S2).

### Cardenolides

The cardenolides belong to a group of steroidal compounds and referred as cardiac glycosides because of their cardiotonic properties. The chemical structures of cardenolides contains five-membered butenolide ring attached to C-17 of the basic steroid part of the aglycone^78^. Three cardenolide glycoside (peaks **21**, **32**, **33**) have been identified in the samples of *T. govanianum*. Peak **32** showed the (M+H) ^+^ ion at *m/z* 537.30, and fragments at *m/z* 519.29, 391.24, and 373.23, which were due to (M+H-H_2_O) ^+^, (M+H-Rha) ^+^, and (M+H-Rha-H_2_O) ^+^ ions, respectively. These fragmentation leads for the identification of the peak as rhodexin A (peak **32**)^79^. Further, MS spectra of peak **21** and peak **33** showed the (M+H) ^+^ ion at *m/z* 681.34 and 519.29, which were identified as canarigenin 3-[glucosyl-(1 → 4)-6-deoxy-alloside] and corchoriside B, respectively (Table 2)^80,81^.

**Steroids.** Seventeen compounds (peaks **20**, **23**, **35**, **50**, **54**, **61**, **62**, **66**, **67**, **69**, **89**, **90**, **93**, **95**, **98**, **102**, **103**) were identified as steroidal compounds^3,33,82–90^. Peaks **23**, **69** were identified as steroidal glycosides^83,89^, while peak **95** was identified as triterpenoid saponin^33^. The fragmentation pattern of these molecules has been depicted in Table 2.

### Miscellaneous

Apart from those listed above, another four compounds identified were nucleobase (peak **2**), heterocyclic organic compound (peak **27**), vitamin D derivative (peak **30**) and fatty acids (peak **94**) (Table 2 and Table S2). Peaks **2**, **27**, **30**, and **94** showed the molecular ion peak (M+H) ^+^ at *m/z* 127.05, 409.18, 445.29, 279.23, and MS fragmentation pattern gave the identification as thymine (peak **2**)^3^, khellol glucoside (peak **27**)^91^, 1,25-dihydroxyvitamin D3-26,23-lactone (peak **30**)^92^, *α*-linolenic acid (peak **94**) (Fig. 3A, B)^93^.

### Metabolite profiling by METLIN data base

**Figure 4:**
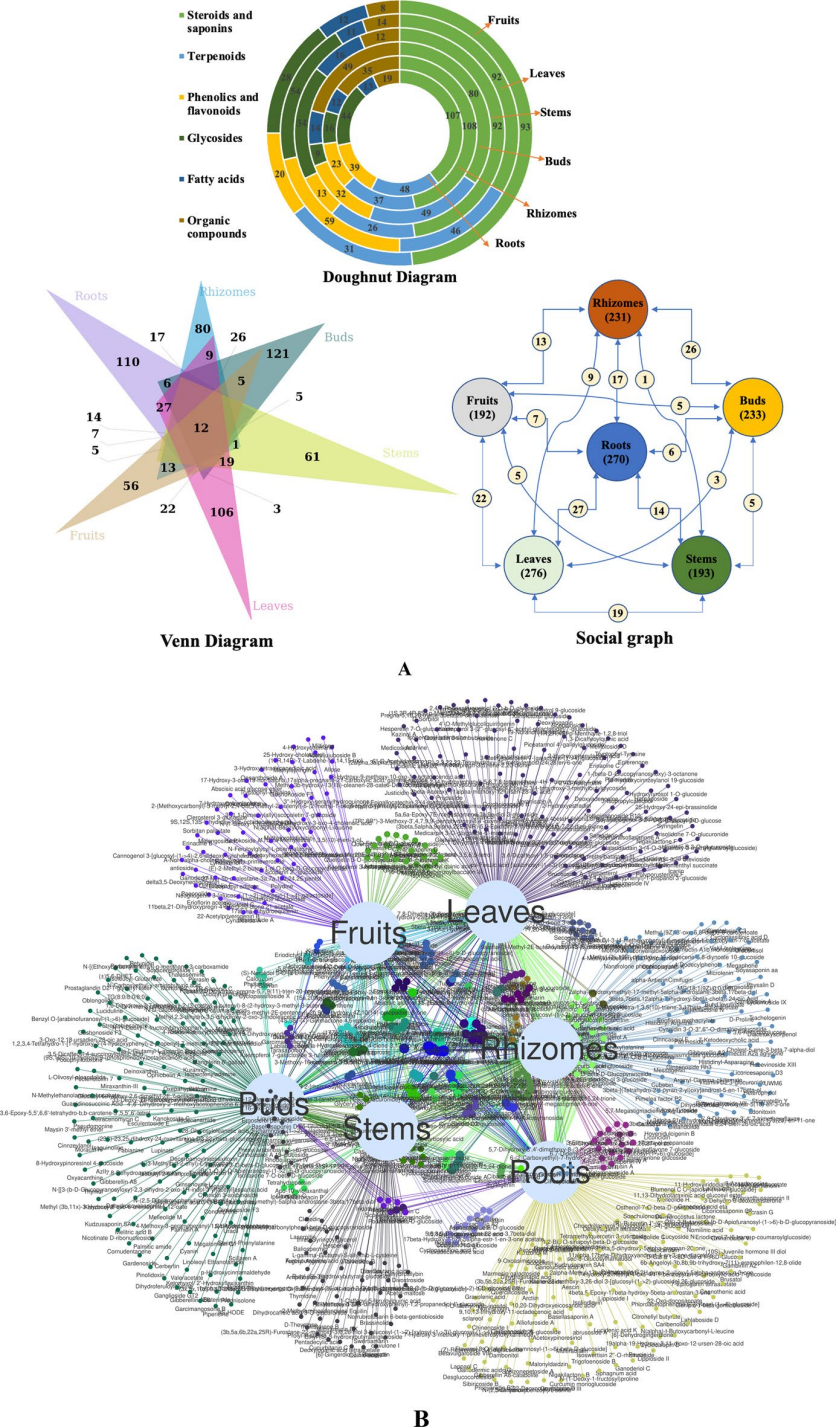
(**A**) Doughnut diagram, Venn diagram, and social graph of metabolites identified using METLIN database in different organs of *T. govanianum*. (**B**) Venn network plot of identified compounds (METLIN database) in different organs of *T. govanianum.*

The acquired raw data from UHPLC-QTOF-IMS were searched against the METLIN database for the tentative identification of different classes of compounds. Accuracy score > 95% was selected for the identification of metabolites and total 839 compounds were identified through METLIN database in different samples such as roots (270), rhizomes (231), rhizomatous buds (233), stems (193), leaves (276), fruits (192) of *T. govanianum* (Table S3). Most of these compounds were commonly present in different organs of the plant. These identified metabolites were further classified in six different categories of compounds comprising of saponins (steroidal, triterpenoid, and their derivatives; 289 metabolites), terpenoids (mono, bi, di, sesqui terpenoids; 140), glycosides (carbohydrates and derivatives; 110 metabolites), fatty acids (fatty esters, acid, saturated, and unsaturated fats; 48 metabolites), phenolics and flavonoids (138 metabolites) and other (organic, nitrogen-containing metabolites including amino acids and nucleobases; 114 metabolites). Doughnut diagrams showed the numbers of metabolites of different categories present in the six organs of the plant. The circles from inner to outsides represented the samples of roots, rhizomes, buds, stems, leaves and fruits, respectively. Among all the parts, roots and rhizomes were dominated with “steroids and saponins” (107 and 108). Similarly, leaves were found enriched with glycoside (54), phenolics and flavonoids (59) along with steroids and saponins (92) (Fig. 4A). Further, similarity and variation of metabolites among all the organs shown in venn diagram and social graph (Fig. 4A). Twelve metabolites were observed common in all the organs, whereas each organ contain unique metabolites viz. roots, rhizomes, rhizomatous buds, stems, leaves, and fruits were found enriched with 110, 80, 121, 61, 106, 56 metabolites, respectively (Fig. 4A). Social graph illustrated that the 80 metabolites of leaves were commonly present in different organs i.e., roots, rhizomes, rhizomatous buds, stems, and fruits contained 27, 9, 3, 19, and 22 common metabolites. Similarly, roots and rhizomes had 71 and 66 common metabolites with different organs parts of the plants (Fig. 4A). Furthermore, venn network plot indicated the relation of 839 identified metabolites with the different organs of *T. govanianum* (Fig. 4B).

### Multivariate statistical analysis

**Figure 5:**
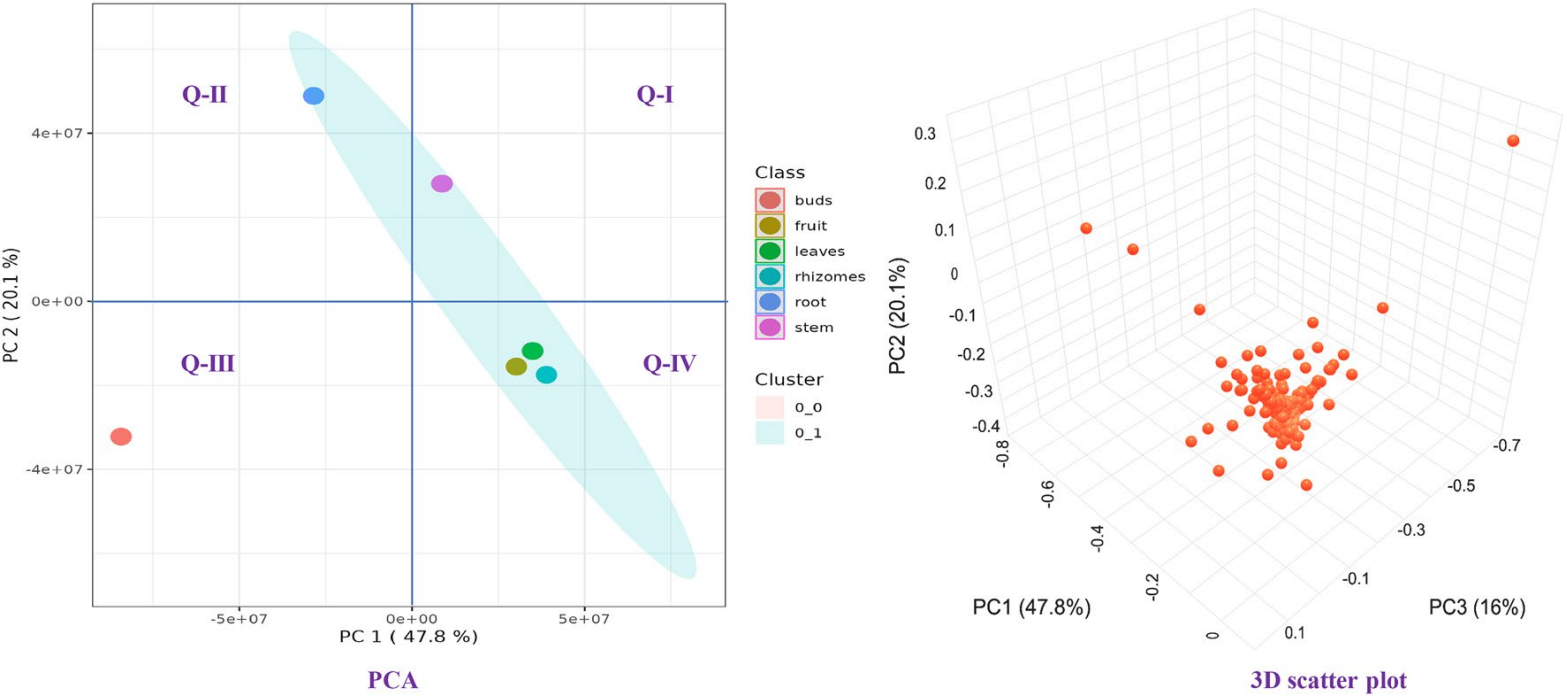
Principal component analysis (PCA) and 3D scatter plot of *T. govanianum* organs.

**Figure 6:**
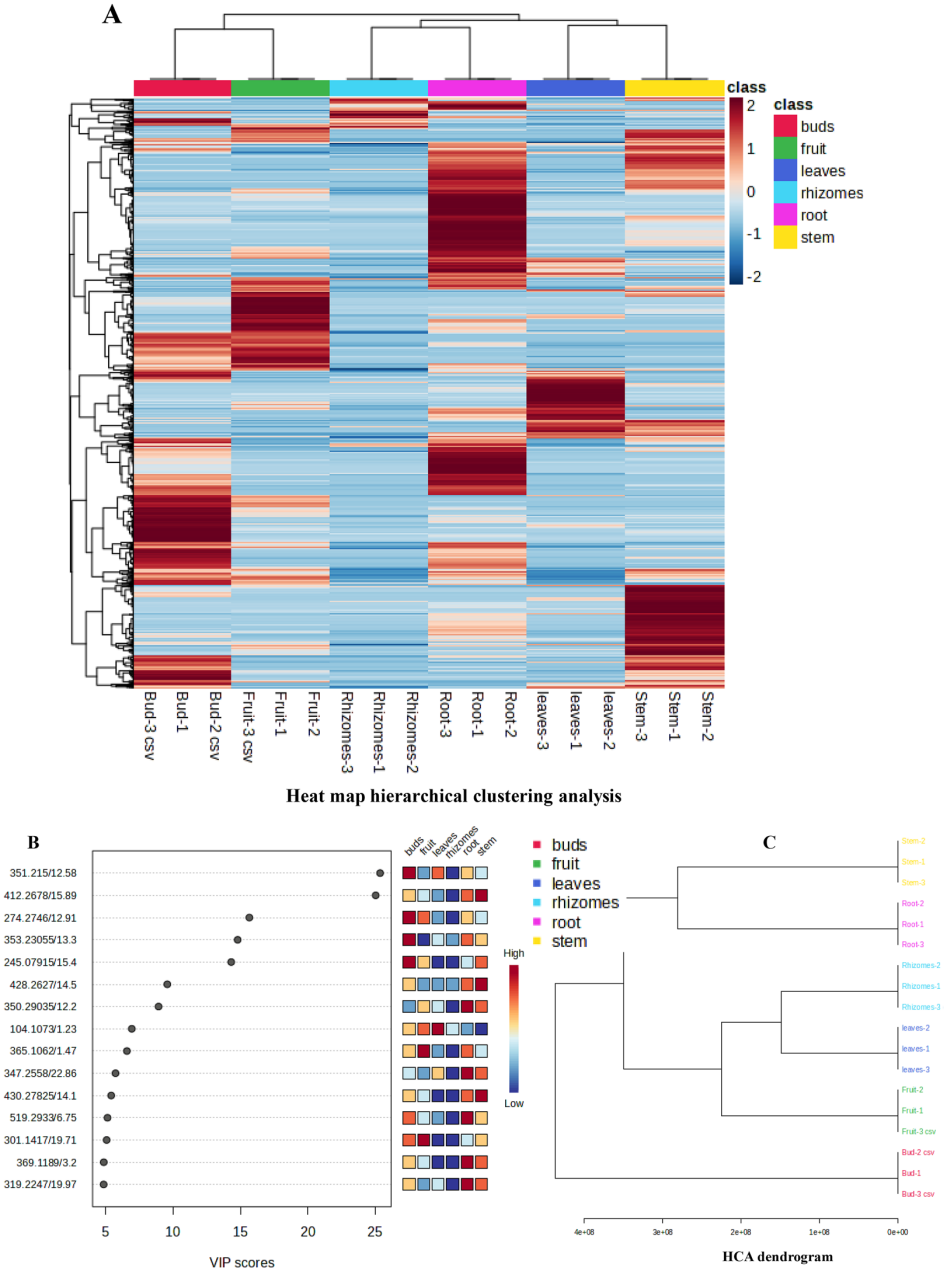
(**A**) Heat map-HCA, (**B**) VIP scores, and (**C**) HCA dendrogram analysis in the different organs of *T. govanianum.* *Heat map hierarchical clustering analysis, content of each metabolite was normalized to the complete linkage hierarchical clustering. Each example is visualized in a single column and each metabolite is represented by a single row. Brown indicates high abundance, whereas metabolites with low relative abundance are shown in blue. (**B**) Identified mass-to-charge ratio (*m/z*) with the variable importance in projection (VIP) scores that discriminate between specific organs. (**C**) Dendrogram of the investigated organs specific samples based on the metabolites obtained after MS data analysis.

Prior to the differential analysis, a principal component analysis (PCA) was conducted on data obtained from METALIN database to observe the degree of variation and similarity between different organs. PCA was used to identify data patterns and the analysis showed the data points were closely grouped or overlapped that demonstrated its good reproducibility. The first two principal components PC1 and PC2 explained 47.8% and 20.1% of the variability in the dataset, respectively, and showed the association with different organs. In the PCA plot, rhizomatous buds were concentrated on the left lower quadrant (Q-III) of the plot, replicates of fruits, leaves, and rhizomes were distributed on the right lower quadrant (Q-IV), while stem and roots were distributed in the upper quadrants (Q-I & Q-II) (Fig. 5). Similarly, 3D scatter plot of PCA showed metabolites proliferation in three axes PC1, PC2, PC3 with percent variation accounted for PC-3 was 16% (Fig. 5). Further, a hierarchical cluster analysis (HCA) was plotted using the *Z*-score normalized metabolite content and showed the relationships of 839 metabolites with 6 different organs of *T. govanianum*. Metabolites with the same characteristics were identified using euclidean distance and were grouped according to complete linkage, following the intergroup variation of the metabolite characteristics were assessed. Heat map hierarchical clustering analysis showed the three main groups clustering along the horizontal direction. The first group included stems and leaves, the second group included roots and rhizomes and the third group included rhizomatous buds and fruits (Fig. 6A). This grouping illustrated in heat map represented the same characteristics of the metabolites along with intergroup variation of the metabolites were assessed along the vertical direction. The brown areas indicated the availability of specific compounds between samples. Identified metabolites (in METLIN database) were arranged based on their mass to charge ratio (*m/z*) and contributed significantly for the differentiation among the organs of *T. govanianum.* Metabolites were extracted by using VIP scores as a quantitative estimation of the discriminatory power of each individual metabolite. Overall, the top 15 metabolites with a VIP score greater than 5 were considered for the organ-specific informative metabolite markers (Fig. 6B). These metabolites suggest organ-specific alterations in polypodine B, steroid derivatives and fatty acids. VIP scores clearly showed the content of octadecanoic acid (*m/z* 351.215) and polypodine B (*m/z* 519.293) among the various organs of *T. govanianum*. It was observed that content of polypodine B was highest in roots followed by rhizomatous buds of *T. govanianum* (Fig. 6B)*.* Further, HCA dendrogram clearly showed that the samples of rhizomatous buds were dramatically different as compared to other organs of *T. govanianum* (Fig. 6C). Thus, the PCA, HCA, VIP score plot and HCA dendrogram results suggested that metabolites differences may be responsible for the variation between samples grouping.

### Quantified metabolites statistical analysis

The statistical analysis (heatmap, Ven-diagram, stacked charts, PCA, PCoA) were performed on targeted metabolites which showed similarities, discriminations, distributions, and variations among the different organs of *T. govanianum*. MVA using heat map and ballon plot clearly showed the differentiation among roots, rhizomes, rhizomatous buds, leaves, stems, and fruits samples of *T. govanianum*. The analysis provided useful information for the quantified metabolites and it was observed that greater the quantity of metabolites, greater will be the size of ballon/ heat colour. The results clearly showed that 20-hydroxyecdysone was present in all organs of *T. govanianum* while diosgenin was found only in rhizomes. Further, ballon plot showed that rhizomes were found enriched with dioscin and fructose, while fruits were rich in fructose, glucose, and galactose (Fig. S6). Further, correlation among various organs were shown by correlation diagram which provided the probability of similarities among various organs of the plant. It clearly showed that roots were closely associated with stems (*P* = 0.81) and leaves (*P* = 0.95), while rhizomes were in association with rhizomatous buds (*P* = 0.77) and fruits (*P* = 0.67). Among all samples, rhizomatous buds and stems were found closely associated with all the parts with *P* = 0.23–0.81. Normal probability plots also showed correlation coefficient in the range of 0.614–0.837 (Fig. S6).

### Cholinesterase inhibitory activity

**Figure 7:**
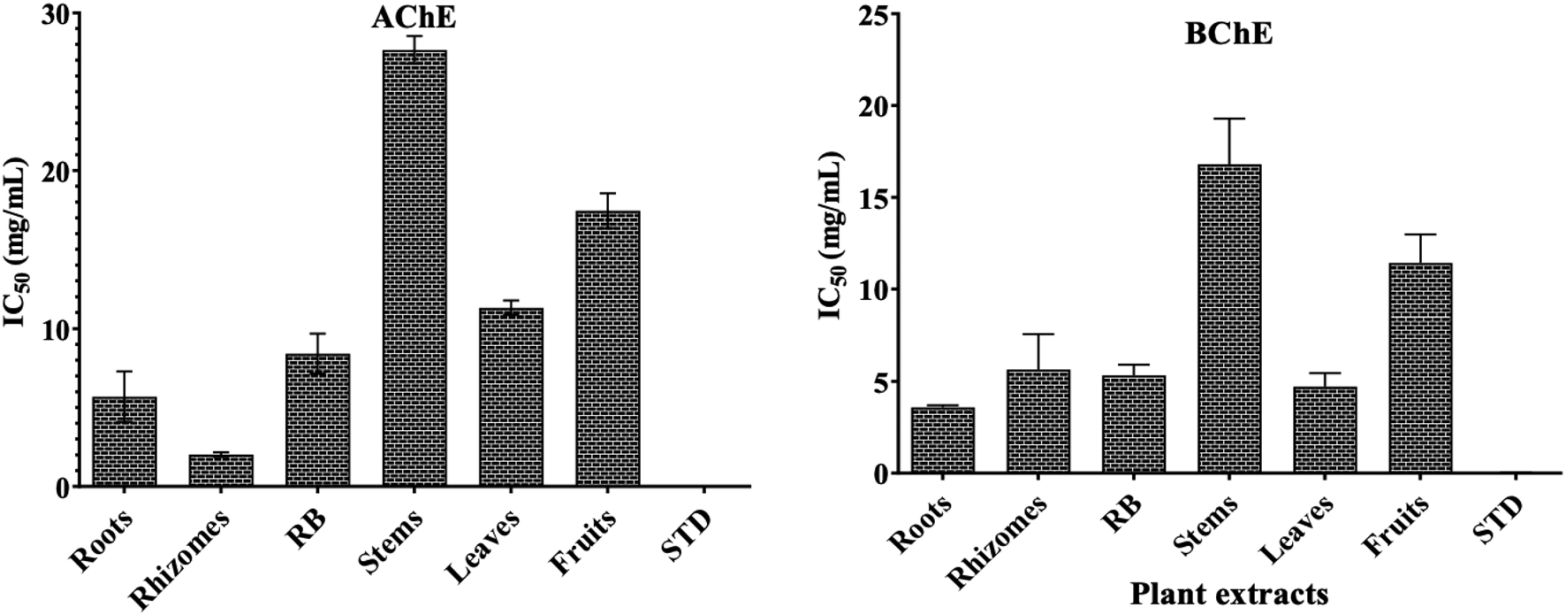
Cholinesterase (AChE and BChE) enzyme inhibition (IC_50_) activity of different organs of *T. govanianum.*

Acetylcholinesterase (AChE) is found at postsynaptic neuromuscular junctions and immediately breaks down acetylcholine (natural neurotransmitter) into acetic acid and choline. These AChE helps to terminate neuronal transmission and signalling that causes problems in communication of neuronal signals and sometime causes Alzheimer’s disease (AD). To prevent this neurotransmitter degradation through enzymes we had targeted to screen the samples for AChE and BChE enzymes inhibitory activity by *in-vitro* enzyme inhibition assays. The effect of *T. govanianum* samples were expressed as percentages of inhibition and IC_50_ values were depicted in Table 3. The results of AChE inhibition by the extract revealed that among different samples, underground part extracts viz. rhizomes (IC_50_: 2.02 ± 0.15 mg/mL) exhibited highest inhibitory effect followed by roots (IC_50_: 5.69 ± 1.61 mg/mL) and rhizomatous buds (IC_50_: 8.42 ± 1.27 mg/mL) as compared to positive control i.e., galantamine (IC_50_: 3.6 ± 0.6 µg/mL) (Fig. 7). However, aerial parts i.e. stem, leaves, and fruits showed inhibition in the ranges of IC_50_: 11.30–27.65 mg/mL. Similarly, underground parts such as roots (IC_50_: 3.58 ± 0.12 mg/mL) showed highest BChE inhibition followed by leaves (IC_50_: 4.72 ± 0.73 mg/mL) as compared to positive control galantamine (IC_50_: 32.0 ± 1.6 µg/mL; Table 3).

**Table 3:** Cholinesterase inhibitory activity of different organs of* T. govanianum.*

Activity	IC_50_ (mg/mL)						
	Roots	Rhizomes	RB	Stem	Leaves	Fruit	Galantamine
AChE	5.69 ± 1.61	2.02 ± 0.15	8.42 ± 1.27	27.65 ± 0.89	11.30 ± 0.46	17.45 ± 1.11	0.0036 ± 0.0006
BChE	3.58 ± 0.12	5.65 ± 1.93	5.32 ± 0.60	16.81 ± 2.48	4.72 ± 0.73	11.43 ± 1.56	0.0320 ± 0.0016

*Data shown as mean ± SD, RB = Rhizomatous buds, AChE = acetylcholinesterase*,* BChE = butyrylcholinesterase.

## Discussion

The metabolomics study of the different parts of the *T. govanianum* were not conducted earlier and only the metabolomics of underground parts were reported^3^, because of the utilization and trade of underground parts in the market. Except few traditional uses in tribal communities the aerial parts were unutilized and waste for the traders and farmers. Therefore, to explore the potential of whole plants of *T. govanianum*, especially the aerial parts, the current study was designed to conduct a comprehensive metabolome analysis of different parts/ organs of aerial and underground parts. The comprehensive metabolome suggested that underground parts contain steroids, sugars and elements while aerial parts are rich in polyphenols, steroids, terpenoids and their derivatives. Earlier study also suggested that total phenolics, flavonoids as well as saponin were estimated in different aerial and underground parts. Polyphenolics were also profiled in both the samples^5^. In current study an accurate and simple UPLC-PDA method was developed, validated and used for the profiling of steroidal compounds (20-hydroxyecdysone, pennogenin-3-O-*β*-chacotrioside, dioscin, trillin, and diosgenin) in different parts samples of *T. govanianum*. The 20-hydroxyecdysone (phytoecdysteroids) was previously reported in water and ethanol extract of *T. govanianum* rhizomes^3^ and our findings also revealed its presence in all samples. Further, dioscin is an important steroidal saponin and a well-known precursor for the synthesis of hormones as well as various synthetic contraceptives in pharmaceutical industries^12^. Dioscin and trillin can be converted into diosgenin (a cortico-steroid hormone) by hydrolysis process. Diosgenin is a precursor for the synthesis of progesterone (sex hormone) and also present in genus *Trillium*^11^. The dioscin content was found very high in rhizomes 33–34% and to best of our knowledge this is the first report for dioscin from *T. govanianum*. Further, the presence of sex hormones precursors in *T. govanianum* supports its traditional claim in the treatment of sexual disorders^11^. However, trillin was not detected in any sample of *T. govanianum*. This might be due to the low quantity of trillin in plant extracts or might be formed as a hydrolytic product of dioscin or related compounds. Similarly, free sugars analysis showed the presence of 2-deoxy-ribose, fructose, glucose, and galactose. Higher amount of fructose in rhizomes (454.76 ± 12.14 mg/g) and fruits (338.74 ± 5.94 mg/g) play an important role in the overall structural growth and enhance their tolerance to abiotic stresses like cold, drought and salinity^94^. Presence of sufficient amount of free sugars provide ability to plant for sustainability at high altitude environmental conditions^22^. Further, non-targeted metabolomics based on UHPLC-Q-TOF-IMS successfully profiled six organs/parts of *T. govanianum*. Total **103** metabolites were identified tentatively in *T. govanianum* samples and comprised of **6** carbohydrates, **11** terpenoids, **4** polyphenols, **5** flavonoids, **73** steroids and saponins, and **4** other organic compounds. Previously, UHPLC-Q-TOF-IMS-based metabolites profiling was able to report only 26 metabolites^3^ and currently it was 103. This huge information on the metabolites will also be helpful to guide for isolation of metabolites as well as to set quality traits of the plant. Moreover, 839 metabolites of different classes were also identified and profiled using METLIN database search and that provided the beneficial information of metabolic flux among the different parts. The statistical analysis provided visual information about similarities and differences among the different samples in terms of organs. Furthermore, samples were screened for cholinesterase inhibitors through enzyme inhibition assays. As per the clinical evidences, cholinesterase inhibitors are one of the most capable treatments for neurodegenerative disorder especially, Alzheimer’s disease^95^. Both AChE and BChE are the targets for inhibition because AChE predominates over BChE. As disease progresses, the activity of AChE declines in certain parts of brain to 10–15% of normal activity, whereas BChE activity increases to compensate the loss in AChE activity. Hence, inhibition of both enzymes is complimentary for treatment of mild-to-severe forms of AD. Plant derived molecules have also been shown cholinesterase inhibitory activity in addition to the approved drugs for AD. Therefore, underground parts (roots, rhizomes, and rhizomatous buds) showed better inhibition to both AChE and BChE enzymes as compared to the aerial parts (stems, leaves, and fruits). These variations might be due to the presence of different metabolites in different extracts and also the different mode of action for the inhibition of the enzymes. In nutshell, *T. govanianum* shows potential to inhibit cholinesterase enzyme and prevent the degradation of acetylcholine neurotransmitter.

## Material and methods

### Chemicals and reagents

2-deoxy-rhamnose, arabinose, fructose, galactose, glucose, mannose, myoinositol, rhamnose, and trehalose were purchased from TCI Chemicals (Ind.) Pvt. Ltd. 20-hydroxyecdysone, pennogenin-3-O-*β*-chacotrioside, dioscin, trillin, diosgenin (purity > 98%, were isolated from *T. govanianum* rhizomes at CSIR-IHBT, India. Formic acid, methanol, and acetonitrile were of HPLC-mass grade procured from Merck Ltd, Mumbai, India. Water from a Milli-Q purification system (Millipore, Bedford, MA, USA) was used. Solvents such as water and ethanol used were of analytical grade.

### Collection and identification of plant material

For collection of plants, all relevant permits/permissions have been obtained. In this study fresh plant materials of *T. govanianum* were collected from farmers’ fields at Rajgundha of Barot valley, Distt-Kangra, in the month of August 2020. The experimental research and field studies on plants, including the collection of plant material comply with the institutional, national, and international guidelines and legislation. The specimen of plant was deposited at Biodiversity Division of CSIR-Institute of Himalayan Bioresource Technology, Palampur, H.P. India. The collected specimen was authenticated as “*Trillium govanianum* Wall. ex D. Don” (voucher specimen number: PLP-16470). Further, the aerial (stems, leaves, and fruits) and underground parts (roots, rhizomes, buds) of *T. govanianum* (5 g each) were separated and crushed to powder using mortar pestle. The crushed material of different parts was extracted with ethanol, using percolation for 24 h. Extracts were then filtered, dried under reduced pressure, and kept at 4 °C for further analysis. 10 mg/mL concentration of each extract was prepared in HPLC grade methanol for further analysis of targeted and non-targeted metabolomics. Biological and technical samples were used in triplicates.

### Targeted metabolomics

#### UPLC-PDA based analysis of steroidal compounds

The quantitative and quantitative analysis of five steroids [namely 20-hydroxyecdysone (**1**), pennogenin-3-O-*β*-chacotrioside (**2**), dioscin (**3**), trillin (**4**), diosgenin (**5**)) was performed on Waters UPLC-QTOF Micromass system. The separation of analytes was carried out on ACE Ultra Core 2.5 Super C18 column (2.1 mm × 100 mm and particle size of 2.5 µm) and the column temperature was kept at 30ºC. The mobile phase consisted of water (0.1% formic acid) as solvent A and acetonitrile (0.1% formic acid) as solvent B, with a steep gradient programmed as: 0.0–0.3 min, 18% B; 0.3–5 min, 18–60% B; 5.0–8.0 min, 60–85% B; 8.0–11.0 min, 85–90% B; 11.0–16.0 min, 90% B; 16.0–17.0 min, 90–18% B (initial) and 17.0–19.0 min initial conditioning for next injection. The flow rate and injection volume were kept at 0.27 mL/min and 2 µL, respectively. Wavelength 195 nm was selected for the analysis and the results were expressed as mg of compound/g of dried extract ± SD. Further, method was validated for linearity, sensitivity, precision, recovery, stability, and reproducibility. Eight different concentrations (3.906–500 µg/mL) of stock solutions (0.5 mg/mL) of each compound were used to plot calibration curve. The calibration curve was plotted based on areas *vs* concentration of standards. The intra-day and inter-day precision were used to define repeatability and reproducibility of the method. The intra-day variation was assessed by performing three repetitive injections of the standard solution on the same day, while the inter-day variation was evaluated over three consecutive days. Further, the accuracy of the method was assessed using a recovery test which was calculated by adding the known concentrations of four different concentrations level to the sample and percentage quantitative recovery with the spiked amount was calculated. Further, concurrent qualitative and quantitative analysis of compounds **1–5** were performed in different organs of *T. govanianum*.

#### UPLC-ELSD-based analysis of sugars

Nine sugars viz. 2-deoxy-rhamnose, arabinose, fructose, galactose, glucose, mannose, myoinositol, rhamnose, and trehalose were analysed using Waters Acquity LC/MS-SQD equipped with ELSD. The method comprises of mobile phase A (water) and B (acetonitrile) having isocratic program at 80% B for 20 min. The elution was performed at a solvent flow rate of 0.21 mL/min. Further, UPLC parameters were as follows; column temperature 30.0 °C, gas pressure: 40.0 psi, gas pressure data channel: off, data rate: 10 points per second, gain: 500.0, time constant: 0.100 s, signal output full scale LSU: 2000 LSU, signal output offset: 0.000 mV, auxiliary output source: nebulizer temperature, drift tube temperature set point: 50.0 °C, drift tube temperature limit: 25.0 °C, drift tube data channel: off, column heater temperature limit: 5.0 °C, pulse width: 0.1 s, Rect wave period: 0.2 s; threshold level: 1 s.

#### Macro and microelements analysis

Estimation of macro and micro-elements (Na, Ca, Mn, Cu, Zn, Fe, Mg, and K), along with heavy metals concentrations (Pb, Cd as toxic and Ni, Cr as essential) were determined in the different parts of the plant by using Shimadzu model AA 6300 Atomic Absorption Spectrophotometer (Tokyo Japan). Briefly, each plant part (0.50 g) of rhizomes, roots, rhizomatous buds, stems, leaves, and fruits was placed in a 100 mL volumetric flask, and 14 mL of acids mixture (HNO_3_: H_2_SO_4_: HClO_4_ with ratio of 9:3:1) was added. The estimation of element was performed as described in the AOAC method^96,97^ and results were expressed as mg/g of dried plant material ± SD.

### Untargeted metabolomics of *T. govanianum*

#### UHPLC-QTOF-MS/MS-based identification of metabolites

The metabolites were analysed using 6560 Ion Mobility Q-TOF LC/MS (Agilent, Santa Clara, USA). The separation of analytes was carried out on ACE Ultra Core 2.5 Super C18 column (2.1 mm × 100 mm and particle size of 2.5 µm). The gradient elution method consisted of mobile phases water (0.1% formic acid) as phase A and acetonitrile (0.1% formic acid) as phase B, with a steep gradient programmed as: 0.0–0.3 min, 10% B; 0.3–8 min, 45% B; 8.0–12.0 min, 60% B; 12–16 min, 85% B; 16.0–18.0 min, 90% B; 18–27 min, 90% B, and 27.0–27.5 min, 10% (initial), 27.5–30.0 min initial conditioning for next injection. The flow rate and injection volume were kept at 0.21 mL/min and 2 µL, respectively. Further, mass spectrometry parameters were in positive mode as follows. Ionization mode: ESI positive, ion Source: dual AJS ESI, gas temperature: 300 °C, gas flow: 5.0 L/min, nebulizer gas pressure: 35 psig, nozzle voltage: 1000 V, capillary voltage: 3500 V, fragmentor voltage: 400 V, scan range of MS spectra: 100–1700 m*/z* with 1.00 spectra/sec speed, isolation width MS/MS: 4 amu, MS threshold: 200 Abs, MS/MS Abs. threshold: 5, ion mobility mode: QTOF.

In metabolites profiling, the data was obtained in positive ion mode and processed through Mass Hunter software. Initially, the metabolites were identified using UV spectra, retention time, and MS/MS fragmentation. Further, for comprehensive chemical profiling, the data was processed through Mass Hunter (qualitative analysis software) and analysis of molecular features and confirmation of compounds in each sample was performed via the Molecular Feature Extractor (MFE) algorithm. Compounds were identified after fixing each parameter such as: retention time, intensity, mass fragmentation, and searched against the METLIN database to identify unknown metabolites in *T. govanianum* extracts. Further, most prominent metabolites were identified by analysing mass fragmentation, MS/MS patterns, molecular weight, UV absorption, and previous literature reports as well as all compounds were confirmed via the Human Metabolome Database (HMDB) (http://www.hmdb.ca/), METLIN (http://metlin.scripps.edu/), ChemSpider (http://www.chemspider.com/) and Kyto Encyclopedia of Genes and Genomes (KEGG) (http://www.kegg.com/). Identified compounds were looked for the possible structure through high-resolution MS and MS/MS spectrum analysis, and compared with online database.

#### In-vitro anti-Alzheimer’s activity

The enzymes acetylcholinesterase (AChE) and butyrylcholinesterase (BChE) inhibitory activity were performed based on Ellman’s assay^98^. Enzymes hydrolyses the substrates (acetylthiocholine or butyryl thiocholine iodide) and convert them into thiocholine, which reacts with Ellman’s reagent (DTNB) to produce “2-nitrobenzoic-5-mercaptothiocholine” and “5-thio-2-nitrobenzoate” which can be detected at 405 nm of wavelength^99^. Briefly, 160 μL of phosphate buffer (pH 7.0), 20 μL of enzyme (AChE/BChE; 0.22 U/mL), and 20 μL test solution (plant extracts, standard) were incubated for 10 min at 4 °C. Then the reaction was initiated by addition of 10 μL of substrate (acetylthiocholine iodide/butyrylthiocholine iodide 0.68 mM) and 10 μL DTNB (0.03 mmol/L). Thereafter, the reaction mixture was incubated for 30 min at 37 °C and absorbance was recorded at 405 nm in a 96 well microtiter plate. A blank for each run consisted of 200 μL buffer, 10 μL substrate and 10 μL DTNB. Each sample were analysed in triplicate. Inhibition percentage of AChE/BChE was determined using the formula:

$${\text{Inhibition}}\,{\text{Percentage }}\left( \% \right) = \left( {\text{C - T}} \right)/{\text{C}} \times {100}$$

where C is the activity of the enzyme without the test sample and T is the activity of the enzyme with the test sample. The results were expressed as IC_50_ ± SD values.

#### Data analysis and visualization

Data were represented as mean ± standard deviation (SD) of three independent experiments. The data is centered, normalized (confidence level > 95%), and subjected for statistical analysis via Multi Experiment Viewer (MeV; v. 4.9.0) and Past 4.02 (v. 1.0.0.0). Further, online statistical analysis such as eVenn, SRplote were also performed. The datasets of non-targeted metabolites were subjected to the SR plot software to generate the heat maps while targeted metabolites were subjected to the Past 4.02 software. For the activity part statistical analysis was carried out using analysis of variance (ANOVA) followed by Dunnett's multiple comparisons test. It was performed using GraphPad Prism software (v.8.0; GraphPad Software Inc., San. Diego, CA, USA). Values with* p* ≤ 0.05 were taken as statistically significant.

## Conclusion

In this study, detailed metabolites compositional variation in different parts (roots, rhizomes, rhizomatous buds, stems, leaves, and fruits) of *T. govanianum* was explored for the first time that includes estimation of five steroidal compounds, sugar, and micro/macro elements via UPLC-PDA, UPLC-ELSD, and AAS, respectively. Comprehensively, more than 800 metabolites were identified through manual approach and METLIN data search in *T. govanianum*. Steroids was found most abundant class in the underground parts. Study also suggested that *T. govanianum* is a rich source of dioscin and can be converted easily to trillin and diosgenin. Further both can be converted into progesterone (a corticosteroid hormone). The presence of sex hormone precursor in *T. govanianum* strongly supports its traditional claims, as it is effective in sexual problems. In overall, the metabolomics insights the chemical information of *T. govanianum* that can act be a signature for the quality assessment of *T. govanianum* and its derived products. The study will also useful for agro-biotechnological interventions for sustainable cultivation. Furthermore, *T. govanianum* showed anti-alzheimer potential and can be targeted for the isolation of lead anti-alzheimer agents. The study will pave a way for the development phytopharmaceutical product.

## Data availability

All data used/analysed to support the findings of this study are included in this article and its supplementary material files or available from the first author or corresponding author on reasonable request.

### Supplementary Information

Supplementary Information.

## Abbreviations

Rt: Retention time
UV: Ultraviolet
IMS: Ion mobility separation
UPLC-PDA: Ultra performance liquid chromatography photo diode array
ESI-MS: Electro spray ionisation mass spectrometry
UHPLC-QTOF-IMS: Ultra high-performance liquid chromatography -quadrupole time of flight mass spectrometry
UHPLC: Ultra-high performance liquid chromatography
PCA: Principal component analysis
HCA: Hierarchical cluster analysis
AOAC: Association of analytical communities
HMBD: Human Metabolome Database
AD: Alzheimer’s disease
