# Supplementary material for: Organs-specific metabolomics and anticholinesterase activity suggests a trade-off between metabolites for therapeutic advantages of Trillium govanianum Wall. ex D. Don
Source: Sci Rep. 2024 May 9;14:10675. doi: 10.1038/s41598-024-61160-w (PMC11082168; doi:10.1038/s41598-024-61160-w)
Supplement: Supplementary file 1 — Supplementary Information. [file 41598_2024_61160_MOESM1_ESM.docx]

**Supplementary Material**

**Organs-specific metabolomics and anticholinesterase activity suggests a trade-off between metabolites for therapeutic advantages of *Trillium govanianum* Wall. ex D. Don**

Dinesh Kumar^a,b^, Vandana Kumari^a,b^, Dinesh Kumar^a,b^*

^a^Chemical Technology Division, CSIR-Institute of Himalayan Bioresource Technology, Palampur 176 061 (HP), India.

^b^Academy of Scientific and Innovative Research, Ghaziabad-201002, Uttar Pradesh, India

***Corresponding author**

Dr. Dinesh Kumar, Pr. Scientist,

Chemical Technology Division, CSIR-Institute of Himalayan Bioresource Technology, Palampur 176 061 (HP), India. Tel no. +91 1894 230426; Fax no. +91 1894 230433, Mob. +918626824184

Email: sharmadinesh82@gmail.com; [dineshkumar@ihbt.res.in](about:blank)


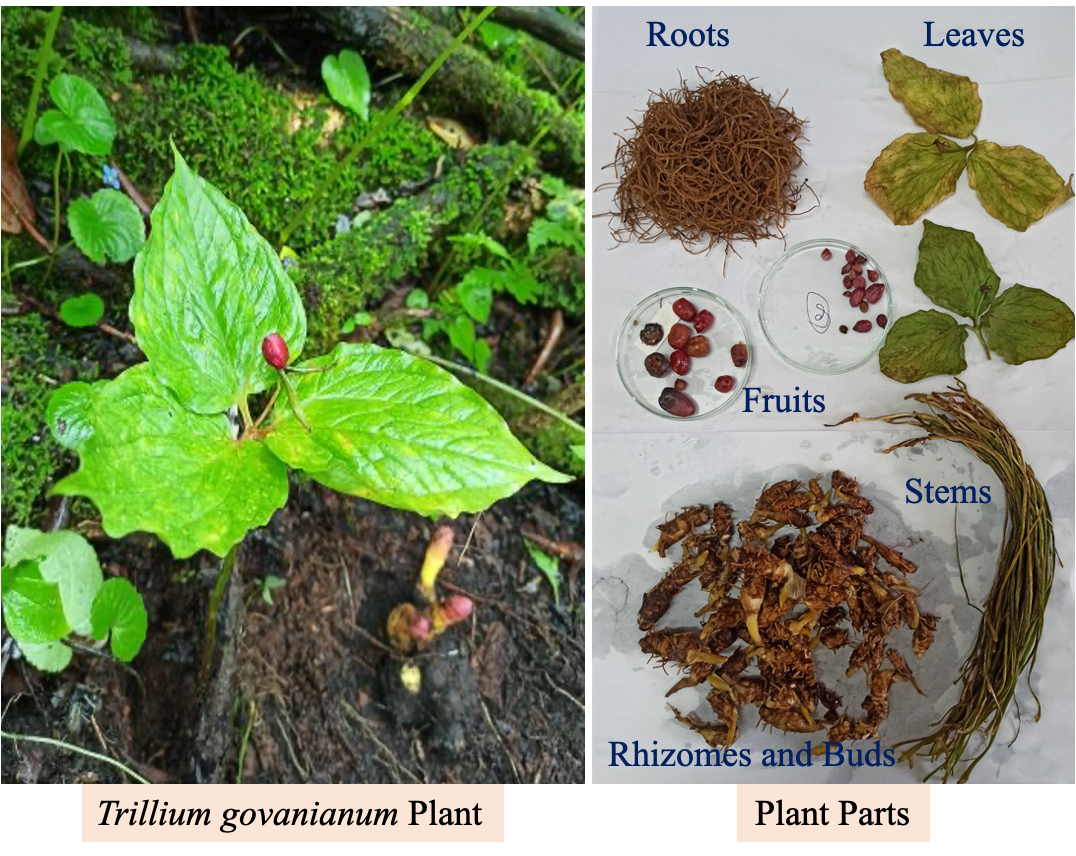


**Fig. S1.** *Trillium govanianum* plant and its separated organs.

*Compound **1** = 20-hydroxyecdysone, Compound **2** = Pennogenin-3-O-*β*-chacotrioside, Compound **3** = Dioscin, Compound **4** = Trillin, Compound **5** = Diosgenin.

**Fig. S2.** Calibration curve of compounds **1-5**.

*2-deoxy-rhamnose (**1**), rhamnose (**2**), arabinose (**3**), mannose (**4**), fructose (**5**), glucose (**6**), galactose (**7**), myoinositol (**8**), and trehalose (**9**).

**Fig. S3.** UPLC-ELSD chromatogram of reference sugar standards and different organs of *Trillium govanianum.*

***A**-Roots, **B**-Rhizomes, **C**- Rhizomatous buds, **D**-Stem, **E**- Leaves, **F**-Fruits.

**Fig. S4.** Total ion chromatogram (TIC) of different organs of *T. govanianum.*

*****20-Hydroxyecdysone **(28)**, Govanoside B (**36**), Anguivioside XV (**41**), Trikamsteroside B **(42)**, Polyphylloside III **(44)**, Pennogenin tetraglycoside **(48)**, Polyphyllin VI (**59**), Pennogenin-3-*O*-*β*-*D*-glucopyranoside (**72**), Stigmasterol-​3-​*O*-*​β*-​D-​glucopyranoside **(75)**, Dioscin (**80**).

**Fig. S5.** Schematic MS fragmentation pattern of identified compounds.


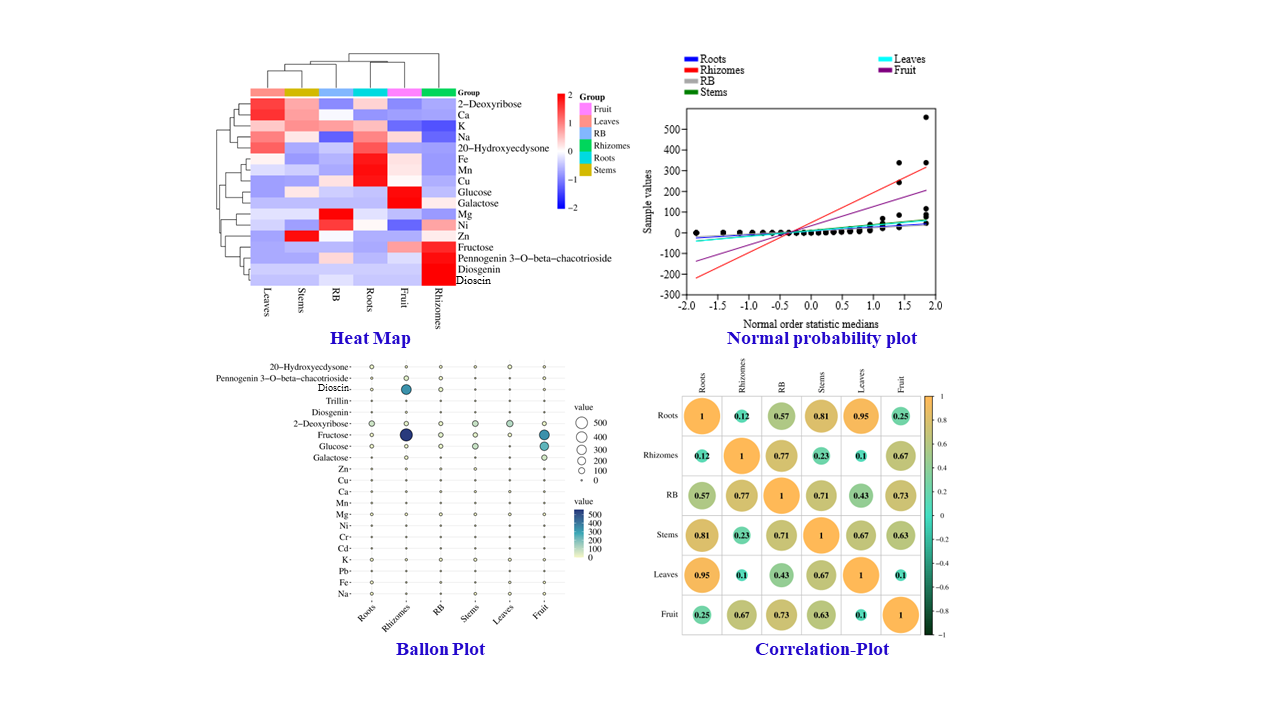


**Fig. S6.** Heatmap, normal probability plot, ballon plot, and correlation plot of quantified metabolites.

**Table S1.** Validation of UPLC-PDA method for the determination of compounds **1-5**.

| **Compounds** | **Regression equation** | **Coefficient of regression (R^2^)** | **LOD (µg/mL)** | **LOQ (µg/mL)** | **Precision** | | **Recovery (%)** | | | **Average recovery (%)** |
| --- | --- | --- | --- | --- | --- | --- | --- | --- | --- | --- |
|  |  |  |  |  | **Intraday**  **(RSD)** % | **Interday**  **(RSD)** % | **R1** | **R2** | **R3** |  |
| **1** | Y = 2915.1*X + 11041 | 0.9986 | 3.81 ± 0.94 | 11.55 ± 2.85 | 1.23 | 2.08 | 88.52 | 98.02 | 102.1 | 96.22 |
| **2** | Y = 2397.4*X + 25422 | 0.9945 | 7.26 ± 1.11 | 22.00 ± 3.38 | 0.89 | 1.08 | 96.22 | 80.03 | 87.03 | 87.76 |
| **3** | Y = 2709.1*X + 19969 | 0.997 | 5.42 ± 0.94 | 16.44 ± 2.84 | 0.94 | 1.30 | 97.59 | 88.38 | 99.14 | 95.04 |
| **4** | Y = 4278.1*X + 25628 | 0.9978 | 4.66 ± 1.58 | 14.12 ± 4.77 | 0.767 | 1.35 | 113.4 | 105.5 | 97.67 | 105.52 |
| **5** | Y = 5879.9*X + 26855 | 0.9989 | 3.35 ± 0.35 | 10.15 ± 1.06 | 0.44 | 0.75 | 101 | 98.16 | 105 | 101.39 |

*Data shown as mean ± SD, **1** = 20-hydroxyecdysone, **2** = Pennogenin-3-O-*β*-chacotrioside, **3** = Dioscin, **4** = Trillin, **5** = Diosgenin, **LOD** = limit of detection, **LOQ** = limit of quantification.

**Table S2.** UPLC-MS/MS chromatogram (ESI+) of identified compounds and their major fragments.

| **S.N.** | **Structure** |
| --- | --- |
| 1 | **** |
| 2 | **** |
| 3 | **** |
| 4 | **** |
| 5 | **** |
| 6 | **** |
| 7 | **** |
| 8 | **** |
| 9 | **** |
| 10 | **** |
| 11 | **** |
| 12 | **** |
| 13 | **** |
| 14 | **** |
| 15 | **** |
| 16 | **** |
| 17 | **** |
| 18 | **** |
| 19 | **** |
| 20 | **** |
| 21 | **** |
| 22 | **** |
| 23 | **** |
| 24 | **** |
| 25 | **** |
| 26 | **** |
| 27 | **** |
| 28 | **** |
| 29 | **** |
| 30 | **** |
| 31 | **** |
| 32 | **** |
| 33 | **** |
| 34 | **** |
| 35 | **** |
| 36 | **** |
| 37 | **** |
| 38 | **** |
| 39 | **** |
| 40 | **** |
| 41 | **** |
| 42 | **** |
| 43 | **** |
| 44 | **** |
| 45 | **** |
| 46 | **** |
| 47 | **** |
| 48 | **** |
| 49 | **** |
| 50 | **** |
| 51 | **** |
| 52 | **** |
| 53 | **** |
| 54 | **** |
| 55 | **** |
| 56 | **** |
| 57 | **** |
| 58 | **** |
| 59 | **** |
| 60 | **** |
| 61 | **** |
| 62 | **** |
| 63 | **** |
| 64 | **** |
| 65 | **** |
| 66 | **** |
| 67 | **** |
| 68 | **** |
| 69 | **** |
| 70 | **** |
| 71 | **** |
| 72 | **** |
| 73 | **** |
| 74 | **** |
| 75 | **** |
| 76 | **** |
| 77 | **** |
| 78 | **** |
| 79 | **** |
| 80 | **** |
| 81 | **** |
| 82 | **** |
| 83 | **** |
| 84 | **** |
| 85 | **** |
| 86 | **** |
| 87 | **** |
| 88 | **** |
| 89 | **** |
| 90 | **** |
| 91 | **** |
| 92 | **** |
| 93 | **** |
| 94 | **** |
| 95 | **** |
| 96 |  |
| 97 |  |
| 98 |  |
| 99 |  |
| 100 |  |
| 101 |  |
| 102 |  |
| 103 |  |
|  |  |

**Table S3.** Details of metabolites identified through Metlin database (Confidence level >95%).

| **Name** | **Formula** | **Mass** | **Score (MFE)** | **Root** | **Rhizomes** | **Buds** | **Stem** | **Leaves** | **Fruits** |
| --- | --- | --- | --- | --- | --- | --- | --- | --- | --- |
| Desglucocheirotoxol | C29 H44 O10 | 552.2975 | 99.4 | 89065 | 0 | 0 | 676269 | 360101 | 0 |
| (25S)-5beta-spirostan-3beta-yl beta-D-glucoside | C33 H54 O8 | 578.3805 | 100 | 246563 | 0 | 0 | 0 | 0 | 0 |
| Lucidenic acid F | C27 H36 O6 | 456.2522 | 100 | 262831 | 0 | 0 | 49423 | 0 | 0 |
| Uscharidin | C29 H38 O9 | 530.2518 | 100 | 207841 | 0 | 29705 | 0 | 0 | 0 |
| Helveticoside | C29 H42 O9 | 534.2812 | 100 | 66034 | 2744256 | 5396420 | 34420 | 0 | 0 |
| Aescin | C55 H86 O24 | 1130.5496 | 100 | 161170 | 0 | 0 | 0 | 0 | 0 |
| Sarcostin | C21 H34 O6 | 382.2355 | 95.6 | 114706 | 0 | 0 | 0 | 374075 | 0 |
| rhodexin A | C29 H44 O9 | 536.298 | 100 | 64797 | 94784 | 343564 | 0 | 0 | 107316 |
| k-Strophanthin-beta | C36 H54 O14 | 710.3471 | 100 | 65829 | 0 | 0 | 0 | 47643 | 197609 |
| 6,7-Didehydrofevicordin F 3-[glucosyl-(1->6)-glucoside] | C41 H60 O17 | 824.3798 | 100 | 31895 | 29558 | 0 | 0 | 0 | 0 |
| Leontogenin | C27 H42 O5 | 446.3035 | 100 | 127826 | 0 | 0 | 49366 | 52322 | 0 |
| Nomilinic acid | C28 H36 O10 | 532.231 | 100 | 60258 | 0 | 0 | 0 | 0 | 0 |
| 19alpha-19-Hydroxy-3,11-dioxo-12-ursen-28-oic acid | C30 H44 O5 | 484.3211 | 95.4 | 34604 | 0 | 0 | 0 | 0 | 0 |
| Kudzusaponin SA4 | C47 H74 O20 | 958.4745 | 100 | 20500 | 0 | 0 | 0 | 0 | 0 |
| Soyasaponin A2 | C53 H86 O24 | 1106.5471 | 97.2 | 23689 | 0 | 0 | 27988 | 0 | 0 |
| digitogenin | C27 H44 O5 | 448.3185 | 100 | 37987 | 56722 | 0 | 73125 | 0 | 0 |
| Quercilicoside A | C36 H58 O11 | 666.3981 | 100 | 78588 | 0 | 0 | 0 | 0 | 0 |
| Betavulgaroside VIII | C46 H68 O20 | 940.4324 | 94.4 | 19386 | 0 | 0 | 0 | 0 | 0 |
| Assamsaponin E | C59 H92 O26 | 1216.5866 | 100 | 1052446 | 108416 | 0 | 22392 | 103630 | 0 |
| Lablaboside D | C60 H94 O28 | 1262.5919 | 100 | 21608 | 0 | 0 | 0 | 0 | 0 |
| Basellasaponin A | C47 H70 O21 | 970.4405 | 100 | 39566 | 0 | 0 | 0 | 0 | 0 |
| Amaranthussaponin II | C48 H74 O20 | 970.4774 | 100 | 27200 | 0 | 0 | 0 | 0 | 0 |
| Lucidenic acid K | C27 H36 O7 | 472.2467 | 100 | 391440 | 0 | 0 | 0 | 0 | 0 |
| Lucyoside N | C36 H58 O10 | 650.4029 | 100 | 209741 | 0 | 0 | 0 | 0 | 0 |
| Avenacoside A | C51 H82 O23 | 1062.5249 | 100 | 546028 | 0 | 0 | 79497 | 369158 | 0 |
| Licoricesaponin F3 | C48 H72 O19 | 952.4659 | 100 | 35867 | 0 | 0 | 74971 | 154132 | 233051 |
| Basellasaponin B | C47 H68 O21 | 968.4294 | 100 | 51114 | 196954 | 60871 | 32127 | 0 | 0 |
| Cyclopassifloside V | C43 H72 O17 | 860.485 | 100 | 205079 | 0 | 0 | 27242 | 38710 | 0 |
| Caraganoside A | C52 H84 O21 | 1044.5474 | 95.2 | 62968 | 0 | 0 | 0 | 0 | 0 |
| Goyasaponin III | C49 H76 O19 | 968.4952 | 100 | 44205 | 0 | 0 | 0 | 0 | 0 |
| Pisumsaponin I | C51 H80 O21 | 1028.522 | 100 | 165305 | 16970 | 47225 | 112400 | 366205 | 9637 |
| Ganodermic acid TQ | C32 H46 O5 | 510.3337 | 100 | 70173 | 0 | 0 | 0 | 0 | 0 |
| Ginsenoside Ia | C42 H72 O14 | 800.4906 | 100 | 105637 | 99757 | 83484 | 102687 | 88061 | 76552 |
| Cyclopassifloside II | C37 H62 O11 | 682.4282 | 100 | 285694 | 121098 | 117189 | 0 | 135412 | 176158 |
| Ganolucidic acid A | C30 H44 O6 | 500.3147 | 100 | 70286 | 0 | 0 | 0 | 0 | 0 |
| Sanguisorbin B | C35 H56 O7 | 588.4039 | 100 | 202867 | 0 | 0 | 0 | 102298 | 0 |
| Tsugarioside B | C37 H60 O7 | 616.4349 | 100 | 105015 | 0 | 0 | 0 | 252596 | 0 |
| Ganoderiol C | C32 H54 O5 | 518.3978 | 99.8 | 93305 | 0 | 0 | 0 | 0 | 0 |
| Theasapogenol A | C30 H50 O6 | 506.3595 | 100 | 672895 | 200444 | 198907 | 438685 | 1072408 | 148409 |
| Olitorin | C35 H52 O14 | 696.3333 | 100 | 22907 | 0 | 0 | 0 | 0 | 30723 |
| 17beta-Hydroxy-4-oxa-5alpha-estr-1-en-3-one acetate | C19 H26 O4 | 318.1832 | 97.3 | 53273 | 0 | 0 | 34421 | 0 | 0 |
| Ajugasterone C | C27 H44 O7 | 480.3097 | 95.1 | 1857137 | 929856 | 1686748 | 1900864 | 3042674 | 485439 |
| ajugalactone | C29 H40 O8 | 516.2707 | 100 | 302376 | 25725 | 66223 | 0 | 0 | 0 |
| Strophanthidin | C23 H32 O6 | 404.2216 | 100 | 83725 | 0 | 0 | 90464 | 0 | 0 |
| 5α-Cholestane-3α,7α,12α,26,27-pentol | C27 H48 O5 | 452.3485 | 96.8 | 147758 | 72023 | 0 | 0 | 0 | 0 |
| Estra-1,3,5(10)-triene-3,6alpha,17beta-triol triacetate | C24 H30 O6 | 414.2052 | 100 | 1515640 | 118579 | 76329 | 1522957 | 510513 | 1432938 |
| 3-Ethoxyandrosta-3,5-dien-17beta-ol propanoate | C24 H36 O3 | 372.2648 | 95.4 | 130576 | 130506 | 0 | 0 | 0 | 0 |
| Canarigenin 3-[glucosyl-(1->4)-6-deoxy-alloside] | C35 H52 O13 | 680.3388 | 100 | 570988 | 68259 | 29126 | 139944 | 229270 | 28416 |
| Condurango glycoside A | C53 H78 O17 | 986.5236 | 95.6 | 62550 | 60936 | 51607 | 81090 | 0 | 0 |
| Sibiricoside B | C50 H80 O24 | 1064.5014 | 97.8 | 12734 | 0 | 0 | 0 | 0 | 0 |
| 17-alpha-estradiol-3-glucuronide | C24 H32 O8 | 448.2065 | 100 | 523218 | 0 | 0 | 0 | 0 | 165322 |
| polypodine B | C27 H44 O8 | 496.305 | 100 | 40511940 | 333832 | 2230514 | 0 | 551902 | 141882 |
| 6beta,19-Epoxy-17beta-hydroxyandrost-4-en-3-one | C19 H26 O3 | 302.1889 | 98.4 | 81514 | 194307 | 351698 | 99636 | 172127 | 268975 |
| ecdysone | C27 H44 O6 | 464.3147 | 100 | 81539 | 64512 | 0 | 0 | 136642 | 24239 |
| Capsicosin | C57 H94 O29 | 1242.5886 | 100 | 87294 | 0 | 0 | 117576 | 0 | 0 |
| Trigofoenoside B | C45 H76 O19 | 920.4981 | 100 | 122343 | 0 | 0 | 0 | 0 | 0 |
| Trigofoenoside D | C51 H84 O23 | 1064.5395 | 100 | 132849 | 51814 | 0 | 120079 | 98333 | 0 |
| Convallamaroside | C57 H94 O27 | 1210.5993 | 100 | 797347 | 0 | 0 | 103303 | 182847 | 0 |
| (3b,5b,22a,25R)-Furostane-22-methoxy-3,26-diol 3-[glucosyl-(1->2)-glucoside] 26-glucoside | C46 H78 O19 | 934.5094 | 100 | 20833 | 0 | 0 | 0 | 0 | 0 |
| Chinenoside I | C49 H80 O23 | 1036.5125 | 96.7 | 31071 | 0 | 0 | 0 | 0 | 0 |
| diginatin | C41 H64 O15 | 796.4226 | 100 | 41321 | 12392 | 0 | 0 | 0 | 0 |
| (17alpha,23S)-17,23-Epoxy-29-hydroxy-27-norlanosta-1,8-diene-3,15,24-trione | C29 H40 O5 | 468.2874 | 100 | 54298 | 0 | 0 | 43659 | 63536 | 0 |
| Cistocardin | C51 H84 O24 | 1080.5339 | 100 | 40398 | 0 | 0 | 478922 | 0 | 0 |
| Digoxin | C41 H64 O14 | 780.4278 | 100 | 79905 | 49227 | 0 | 0 | 0 | 0 |
| Protodioscin | C51 H84 O22 | 1048.5459 | 100 | 392463 | 0 | 0 | 370028 | 0 | 10268646 |
| Aginoside progenin | C39 H64 O15 | 772.4245 | 100 | 151185 | 0 | 0 | 179285 | 0 | 413546 |
| Chinenoside VI | C44 H70 O19 | 902.4546 | 100 | 43907 | 0 | 0 | 0 | 0 | 0 |
| Amaranthussaponin IV | C47 H70 O20 | 954.4448 | 100 | 130782 | 143611 | 0 | 0 | 0 | 0 |
| Chinenoside II | C49 H78 O22 | 1018.499 | 95.9 | 18695 | 0 | 0 | 0 | 0 | 61067 |
| Coagulin R 3-glucoside | C34 H48 O11 | 632.3187 | 95.2 | 40280 | 0 | 0 | 52596 | 0 | 0 |
| Chondrillasterol 3-[glucosyl-(1->4)-glucoside] | C41 H68 O11 | 736.4755 | 95.6 | 11141 | 0 | 0 | 0 | 0 | 0 |
| Alliofuroside A | C44 H72 O18 | 888.4702 | 96.8 | 14340 | 0 | 0 | 0 | 0 | 0 |
| Melongoside K | C51 H82 O22 | 1046.5302 | 100 | 701388 | 0 | 17817 | 56053 | 297238 | 26113 |
| Fistuloside B | C45 H72 O18 | 900.4732 | 100 | 2181422 | 103288 | 313033 | 71002 | 115025 | 429662 |
| Diosgenin 3-[glucosyl-(1->4)-rhamnosyl-(1->4)-glucoside] | C45 H72 O17 | 884.4771 | 100 | 219147 | 352738 | 65600 | 774855 | 1763692 | 0 |
| Schidigerasaponin C2 | C39 H62 O14 | 754.4143 | 100 | 58914 | 141765 | 269362 | 49932 | 0 | 0 |
| Nuatigenin | C27 H42 O4 | 430.3082 | 96.8 | 198896 | 471373 | 84041 | 0 | 94162 | 0 |
| Desglucocoroloside | C29 H44 O7 | 504.3124 | 96.7 | 106336 | 0 | 0 | 0 | 0 | 0 |
| Asparagoside F | C50 H82 O22 | 1034.5302 | 98.9 | 10575 | 0 | 0 | 17012 | 0 | 0 |
| (3b,5a,6b,25R)-3,6-Dihydroxyspirostane-2,12-dione 3-[4'-(2''-glucosyl-3''-xylosyl)-galactoside] | C50 H78 O25 | 1078.4841 | 100 | 174760 | 26365 | 0 | 30528 | 113510 | 43510 |
| abrusoside A | C36 H54 O10 | 646.3767 | 99.4 | 98748 | 0 | 0 | 0 | 0 | 0 |
| Cortisol | C21 H30 O5 | 362.2079 | 100 | 99990 | 192758 | 180660 | 0 | 2449572 | 59131 |
| 6beta-(Dimethylamino)-3beta,5-dihydroxy-5alpha-pregnan-20-one | C23 H39 N O3 | 377.293 | 100 | 50296 | 0 | 0 | 0 | 0 | 0 |
| 11alpha,17beta-Dihydroxyandrost-4-en-3-one diacetate | C23 H32 O5 | 388.2247 | 100 | 46328 | 0 | 0 | 0 | 0 | 0 |
| 22-Deoxocucurbitacin D | C30 H46 O6 | 502.3301 | 100 | 102908 | 0 | 0 | 0 | 0 | 0 |
| 4beta,5-Epoxy-17beta-hydroxy-5beta-androstan-3-one | C19 H28 O3 | 304.2027 | 97.6 | 18908 | 0 | 0 | 0 | 0 | 0 |
| Cucurbitacin S | C30 H42 O6 | 498.2968 | 100 | 95265 | 20404 | 0 | 0 | 0 | 0 |
| Capsicoside C3 | C44 H70 O17 | 870.4619 | 100 | 16941 | 0 | 0 | 0 | 0 | 0 |
| 28-Homobrassinolide | C29 H50 O6 | 494.359 | 100 | 95996 | 0 | 47859 | 215354 | 0 | 0 |
| 7alpha,24-dihydroxycholest-4-en-3-one | C27 H44 O3 | 416.329 | 95.4 | 42019 | 22991 | 0 | 0 | 0 | 21298 |
| Teasterone | C28 H48 O4 | 448.3534 | 100 | 96188 | 126940 | 0 | 0 | 0 | 88410 |
| (24R)-5b,8b-Epidioxyergosta-6,22E-dien-3b-ol 3-glucoside | C34 H54 O8 | 590.3812 | 100 | 544712 | 0 | 0 | 0 | 62467 | 0 |
| Cyclopassifloic acid C | C31 H52 O7 | 536.3722 | 100 | 839200 | 0 | 0 | 81242 | 0 | 0 |
| (3beta,22R,23R,24S)-3,22,23-Trihydroxystigmastan-6-one | C29 H50 O4 | 462.3692 | 100 | 163672 | 0 | 0 | 99071 | 374719 | 0 |
| 2alpha-Methyl-17beta-[(tetrahydro-2H-pyran-2-yl)oxy]-5alpha-androstan-3-one | C25 H40 O3 | 388.2967 | 100 | 95118 | 0 | 0 | 0 | 0 | 0 |
| 3-Dehydro-6-deoxoteasterone | C28 H48 O3 | 432.3591 | 100 | 52245 | 0 | 0 | 0 | 0 | 0 |
| Norethindrone enanthate | C27 H38 O3 | 410.2829 | 100 | 528888 | 666755 | 95486 | 152821 | 28016 | 0 |
| Sarcoaldesterol A | C30 H52 O4 | 476.3867 | 98.5 | 71236 | 0 | 0 | 0 | 159468 | 0 |
| Cyclopassifloic acid B | C31 H52 O6 | 520.3749 | 100 | 126565 | 0 | 0 | 589218 | 172403 | 0 |
| Elatoside E | C46 H74 O16 | 882.4984 | 100 | 94295 | 0 | 0 | 0 | 0 | 14426 |
| Scopoloside II | C39 H62 O15 | 770.4095 | 100 | 67581 | 0 | 14374 | 0 | 0 | 0 |
| Yamogenin 3-O-neohesperidoside | C39 H62 O12 | 722.4263 | 100 | 92010 | 685142 | 158881 | 44464 | 0 | 19041 |
| Ganoderic acid eta | C30 H44 O8 | 532.3046 | 98.7 | 117394 | 0 | 0 | 0 | 0 | 0 |
| Camellidin II | C53 H84 O24 | 1104.5405 | 100 | 20834 | 137036 | 0 | 0 | 0 | 0 |
| Licoricesaponin C2 | C42 H62 O15 | 806.4072 | 96.6 | 14822 | 0 | 0 | 0 | 0 | 0 |
| Fistuloside A | C39 H62 O13 | 738.4197 | 100 | 151455 | 141914 | 118609 | 171647 | 260567 | 75219 |
| Urocortisol | C21 H34 O5 | 366.2391 | 100 | 0 | 370648 | 0 | 0 | 0 | 0 |
| Tuberoside K | C45 H74 O18 | 902.4863 | 100 | 0 | 82263 | 0 | 217226 | 0 | 170863 |
| Tsugaric acid C | C32 H50 O5 | 514.3664 | 100 | 0 | 86603 | 0 | 74929 | 646821 | 0 |
| TR-Saponin A | C52 H78 O20 | 1022.505 | 96.9 | 0 | 16571 | 0 | 0 | 0 | 13843 |
| Theasaponin | C59 H92 O27 | 1232.5811 | 100 | 0 | 410132 | 35665 | 0 | 0 | 0 |
| Sterol 3-beta-D-glucoside | C23 H38 O6 | 410.2652 | 100 | 0 | 174597 | 38835 | 0 | 279974 | 0 |
| Soyasaponin aa | C53 H82 O21 | 1054.5262 | 100 | 0 | 69977 | 0 | 0 | 0 | 0 |
| Pseudoprotodioscin | C51 H82 O21 | 1030.5363 | 100 | 0 | 7022530 | 1307760 | 292166 | 6216126 | 5946070 |
| Prednisolone tebutate | C27 H38 O6 | 458.2676 | 100 | 0 | 92285 | 73763 | 40146 | 0 | 43416 |
| Physalin D | C28 H32 O11 | 544.1937 | 99.4 | 0 | 7542 | 0 | 0 | 0 | 0 |
| Ophiopogonin A | C41 H64 O13 | 764.4331 | 100 | 0 | 37549 | 0 | 0 | 0 | 0 |
| nuatigenin 3-beta-D-glucopyranoside | C33 H52 O9 | 592.362 | 100 | 0 | 174637 | 43537 | 176579 | 0 | 620521 |
| Neoconvallatoxoloside | C35 H54 O15 | 714.348 | 100 | 0 | 21154 | 0 | 0 | 0 | 0 |
| Nandrolone phenpropionate | C27 H34 O3 | 406.2507 | 98.8 | 0 | 37391 | 0 | 0 | 0 | 0 |
| Momordin Ia | C42 H66 O13 | 778.4477 | 100 | 0 | 20782 | 0 | 0 | 0 | 0 |
| Messagenin | C29 H48 O3 | 444.3593 | 100 | 0 | 35596 | 0 | 0 | 0 | 0 |
| Medicoside F | C47 H76 O18 | 928.4988 | 96.6 | 0 | 17210 | 0 | 0 | 0 | 0 |
| Lucidenolactone | C27 H36 O6 | 456.2524 | 100 | 0 | 24762 | 0 | 0 | 0 | 28510 |
| Lucidenic acid C | C27 H40 O7 | 476.2782 | 100 | 0 | 67062 | 133730 | 82032 | 147877 | 61564 |
| lokundjoside | C29 H44 O10 | 552.2942 | 100 | 0 | 106442 | 83882 | 0 | 0 | 0 |
| Licoricesaponin E2 | C42 H60 O16 | 820.3872 | 100 | 0 | 20188 | 0 | 0 | 0 | 0 |
| Licoricesaponin D3 | C50 H76 O21 | 1012.4845 | 94.5 | 0 | 9948 | 0 | 0 | 0 | 0 |
| Hydrocortisone caproate | C27 H40 O6 | 460.2837 | 100 | 0 | 66506 | 23582 | 0 | 0 | 79660 |
| Hebevinoside XIII | C49 H76 O16 | 920.5117 | 100 | 0 | 16835 | 0 | 0 | 0 | 0 |
| Ginsenoside Rh3 | C36 H60 O7 | 604.4327 | 100 | 0 | 183487 | 0 | 0 | 0 | 0 |
| Fevicordin B 2-[rhamnosyl-(1->4)-glucosyl-(1->6)-glucoside] | C49 H74 O22 | 1014.468 | 98.5 | 0 | 18747 | 27345 | 61894 | 0 | 0 |
| Diosgenin 3-[glucosyl-(1->6)-glucosyl-(1->4)-rhamnosyl-(1->4)[rhamnosyl-(1->2)]-glucoside] | C57 H92 O26 | 1192.5872 | 100 | 0 | 235707 | 1826248 | 73446 | 94580 | 0 |
| Dioscin | C45 H72 O16 | 868.4825 | 100 | 0 | 27850 | 1373982 | 37141 | 366654 | 1672669 |
| Dihydrocortisol | C21 H32 O5 | 364.2232 | 100 | 0 | 45930 | 0 | 0 | 0 | 0 |
| Dehydrotestosterone | C19 H26 O2 | 286.1939 | 96.7 | 0 | 161778 | 141592 | 0 | 0 | 89969 |
| Cyclopassifloic acid D | C30 H48 O6 | 504.3439 | 96.5 | 0 | 82530 | 0 | 0 | 0 | 0 |
| Corchoroside B | C29 H42 O8 | 518.287 | 100 | 0 | 3705799 | 377214 | 0 | 0 | 0 |
| Convallasaponin A | C32 H52 O9 | 580.3596 | 100 | 0 | 636600 | 809110 | 0 | 0 | 613672 |
| Cholest-5-ene-3-beta,7-alpha-diol | C27 H46 O2 | 402.3504 | 100 | 0 | 49642 | 0 | 0 | 0 | 0 |
| Celastrol | C29 H38 O4 | 450.2754 | 100 | 0 | 59120 | 0 | 0 | 0 | 0 |
| Camellioside C | C53 H82 O23 | 1086.5238 | 100 | 0 | 3081248 | 1408573 | 0 | 0 | 0 |
| Betavulgaroside VII | C41 H62 O16 | 810.4019 | 100 | 0 | 25097 | 0 | 0 | 0 | 0 |
| Betavulgaroside IV | C41 H62 O15 | 794.4079 | 100 | 0 | 123726 | 213708 | 0 | 0 | 0 |
| Betavulgaroside II | C41 H60 O15 | 792.3963 | 98.8 | 0 | 84874 | 0 | 0 | 36617 | 0 |
| Asterlingulatoside D | C57 H92 O25 | 1176.5924 | 100 | 0 | 79511 | 149514 | 0 | 0 | 36544 |
| Androst-4-ene-3alpha,17beta-diol diacetate | C23 H34 O4 | 374.2443 | 100 | 0 | 553189 | 495431 | 0 | 0 | 0 |
| Amaranthussaponin III | C47 H72 O19 | 940.4645 | 100 | 0 | 105440 | 190474 | 0 | 0 | 0 |
| alpha-Antiarin | C29 H42 O11 | 566.2706 | 100 | 0 | 21409 | 0 | 0 | 0 | 0 |
| adonitoxin | C29 H42 O10 | 550.274 | 100 | 0 | 29335 | 0 | 0 | 0 | 0 |
| 9alpha-Hydroxyandrosta-1,4-diene-3,17-dione | C19 H24 O3 | 300.174 | 100 | 0 | 30166 | 110767 | 0 | 0 | 40305 |
| 7-Ketodeoxycholic acid | C24 H38 O5 | 406.2705 | 100 | 0 | 106582 | 0 | 0 | 0 | 0 |
| 5beta-Pregnane-3alpha,17alpha,20alpha-triol-11-one | C21 H34 O4 | 350.2446 | 100 | 0 | 59826 | 0 | 0 | 0 | 37645 |
| 5alpha-Androstane-3beta,17beta-diol diacetate | C23 H36 O4 | 376.2599 | 100 | 0 | 1283858 | 0 | 0 | 0 | 90536 |
| 3-Methoxyestra-1,3,5(10),16-tetraene | C19 H24 O | 268.1836 | 100 | 0 | 121811 | 0 | 0 | 0 | 39022 |
| 3-Methoxy-16-octylestra-1,3,5(10)-triene-16beta,17beta-diol | C27 H42 O3 | 414.3141 | 100 | 0 | 1492933 | 263724 | 36890 | 0 | 610052 |
| 3-dehydroecdysone | C27 H42 O6 | 462.299 | 100 | 0 | 733878 | 2271008 | 334976 | 130498 | 51216 |
| 3beta-[(Tetrahydro-2H-pyran-2-yl)oxy]androst-5-en-17beta-ol | C24 H38 O3 | 374.2804 | 100 | 0 | 73101 | 0 | 0 | 0 | 0 |
| 3beta,7beta,12alpha-Trihydroxy-5beta-cholan-24-oic Acid | C24 H40 O5 | 408.2858 | 100 | 0 | 667183 | 0 | 0 | 0 | 0 |
| 3beta,17-Dihydroxy-5alpha-pregnane-11,20-dione 3-acetate | C23 H34 O5 | 390.2412 | 100 | 0 | 137578 | 0 | 0 | 0 | 67274 |
| 3beta,15alpha-Diacetoxylanosta-8,24-dien-26-oic acid | C34 H52 O6 | 556.3746 | 100 | 0 | 187482 | 0 | 0 | 0 | 0 |
| 3alpha-Hydroxy-5beta-pregn-16-ene-11,20-dione 3-acetate | C23 H32 O4 | 372.2308 | 100 | 0 | 116726 | 0 | 0 | 0 | 32553 |
| 3alpha,21-Dihydroxy-D-homo-5beta-pregn-17a(20)-en-11-one | C22 H34 O3 | 346.2494 | 100 | 0 | 3225574 | 0 | 0 | 0 | 0 |
| 2alpha-(Hydroxymethyl)-17-methyl-5alpha-androstane-3beta,17beta-diol | C21 H36 O3 | 336.2669 | 100 | 0 | 107288 | 0 | 0 | 0 | 0 |
| 28-Glucopyranosyl-3-methyloleanolic acid | C37 H60 O8 | 632.432 | 100 | 0 | 100665 | 0 | 0 | 0 | 0 |
| 26-Glucosyl-1,3,11,22-tetrahydroxyergosta-5,24-dien-26-oate | C34 H54 O11 | 638.3679 | 100 | 0 | 42955 | 105936 | 0 | 298974 | 0 |
| 25-Acetylvulgaroside | C27 H42 O7 | 478.2942 | 100 | 0 | 92521 | 494340 | 109989 | 0 | 152262 |
| 17-Propylestra-1,3,5(10)-triene-3,17beta-diol diacetate | C25 H34 O4 | 398.2447 | 99.1 | 0 | 76622 | 0 | 0 | 0 | 0 |
| 17beta-Hydroxyestr-5(10)-en-3-one | C18 H26 O2 | 274.1942 | 100 | 0 | 50400 | 0 | 0 | 0 | 0 |
| 17beta-Hydroxy-2alpha-(methoxymethyl)-17-methyl-5alpha-androstan-3-one | C22 H36 O3 | 348.2653 | 100 | 0 | 75364 | 0 | 0 | 0 | 50321 |
| 16-alpha,17-Epoxypregn-4-ene-3,20-dione | C21 H28 O3 | 328.2044 | 100 | 0 | 93243 | 0 | 0 | 0 | 137436 |
| 11β,21-Dihydroxy-5β-pregnane-3,20-dione | C21 H32 O4 | 348.2285 | 96.1 | 0 | 33954 | 0 | 0 | 0 | 64806 |
| (3beta,17alpha,23S)-17,23-Epoxy-3,29-dihydroxy-27-norlanosta-7,9(11)-diene-15,24-dione | C29 H42 O5 | 470.3007 | 100 | 0 | 105197 | 86998 | 0 | 0 | 0 |
| (23S,24S)-17,23-Epoxy-24,29-dihydroxy-27-norlanost-8-ene-3,15-dione | C29 H44 O5 | 472.3167 | 100 | 0 | 115717 | 99581 | 0 | 58620 | 0 |
| (17Z)-3,11-Dioxopregna-4,17(20)-dien-21-oic acid methyl ester | C22 H28 O4 | 356.1979 | 99.5 | 0 | 32615 | 248847 | 66214 | 112914 | 0 |
| (15a,20R)-Dihydroxypregn-4-en-3-one 20-[glucosyl-(1->4)-6-acetyl-glucoside] | C35 H54 O14 | 698.3494 | 100 | 0 | 297271 | 72473 | 0 | 444501 | 0 |
| Vinaginsenoside R13 | C48 H84 O20 | 980.5537 | 98.8 | 0 | 0 | 19109 | 0 | 0 | 0 |
| TR-Saponin C | C54 H82 O21 | 1066.5329 | 95.5 | 0 | 0 | 19708 | 0 | 0 | 0 |
| Scillaren A | C36 H52 O13 | 692.3425 | 99 | 0 | 0 | 27072 | 0 | 0 | 0 |
| Prednisolone | C21 H28 O5 | 360.1926 | 95.9 | 0 | 0 | 41369 | 0 | 0 | 0 |
| Physalin O | C28 H32 O10 | 528.2018 | 92.4 | 0 | 0 | 61972 | 0 | 0 | 0 |
| Phenolic steroid | C18 H24 O | 256.1831 | 100 | 0 | 0 | 182285 | 0 | 59115 | 0 |
| Ophiopogonin D | C44 H70 O16 | 854.4662 | 98.3 | 0 | 0 | 37486 | 0 | 60192 | 30973 |
| Gamabufogenin | C24 H34 O5 | 402.2389 | 96.9 | 0 | 0 | 32589 | 0 | 0 | 0 |
| Fistuloside C | C45 H72 O19 | 916.4537 | 97.7 | 0 | 0 | 13097 | 32677 | 96333 | 0 |
| Elaterinide | C38 H54 O13 | 718.3535 | 100 | 0 | 0 | 29126 | 176233 | 0 | 0 |
| Cucurbitacin P | C30 H48 O7 | 520.3379 | 96.9 | 0 | 0 | 54319 | 0 | 0 | 0 |
| 3beta,21-Dihydroxy-pregna-5,7,9(11)-trien-20-one diacetate | C25 H32 O5 | 412.2261 | 100 | 0 | 0 | 138972 | 0 | 0 | 33874 |
| 3beta,19-Dihydroxyandrost-5-en-17-one 3-acetate | C21 H30 O4 | 346.215 | 100 | 0 | 0 | 518312 | 494043 | 0 | 453090 |
| 2alpha-(Hydroxymethyl)-5alpha-androstane-3beta,17beta-diol | C20 H34 O3 | 322.2512 | 99.3 | 0 | 0 | 21691 | 148072 | 0 | 0 |
| (23S)-23,25-dihdroxy-24-oxovitamine D3 23-(beta-glucuronide) | C33 H50 O10 | 606.3391 | 100 | 0 | 0 | 80256 | 0 | 0 | 0 |
| Periandrin V | C41 H62 O14 | 778.412 | 95.6 | 0 | 0 | 87966 | 0 | 0 | 0 |
| Medicagenic acid 3-O-b-D-glucuronide | C36 H54 O12 | 678.36 | 95.5 | 0 | 0 | 34161 | 0 | 0 | 0 |
| Ginsenoside F3 | C41 H70 O13 | 770.4812 | 100 | 0 | 0 | 36632 | 0 | 0 | 0 |
| Esculentoside E | C35 H54 O11 | 650.3643 | 96.2 | 0 | 0 | 19792 | 0 | 0 | 0 |
| Dehydrosoyasaponin I | C48 H76 O18 | 940.5003 | 95.3 | 0 | 0 | 10762 | 0 | 0 | 0 |
| AzII | C54 H82 O23 | 1098.5242 | 90 | 0 | 0 | 11563 | 0 | 0 | 0 |
| 3-Oxo-12,18-ursadien-28-oic acid | C30 H44 O3 | 452.3271 | 95.6 | 0 | 0 | 54777 | 0 | 0 | 0 |
| Kudzusaponin SA3 | C53 H86 O23 | 1090.5494 | 96.6 | 0 | 0 | 147402 | 0 | 0 | 0 |
| Cerbertin | C32 H44 O11 | 604.2864 | 100 | 0 | 0 | 19427 | 0 | 0 | 0 |
| Calotropin | C29 H40 O9 | 532.2646 | 98.8 | 0 | 0 | 19950 | 0 | 0 | 22952 |
| Divostroside | C30 H46 O8 | 534.3181 | 98.8 | 0 | 0 | 0 | 31891 | 0 | 0 |
| Hydrocortisone cypionate | C29 H42 O6 | 486.2985 | 100 | 0 | 0 | 0 | 110713 | 85486 | 0 |
| Polygalacin D | C57 H92 O27 | 1208.5829 | 100 | 0 | 0 | 0 | 192280 | 348781 | 0 |
| (3b,5a,6b,22a,25R)-Furostane-22-methoxy-3,6,26-triol 3-[glucosyl-(1->2)-[xylosyl-(1->3)]-glucosyl-(1->4)-galactoside] 26-glucoside | C57 H96 O29 | 1244.6019 | 100 | 0 | 0 | 0 | 32780 | 0 | 0 |
| 5,6:8,9-Diepoxyergost-22-ene-3,7beta-diol | C28 H44 O4 | 444.3236 | 100 | 226668 | 0 | 0 | 78232 | 0 | 0 |
| Capsicoside C | C52 H88 O25 | 1112.5549 | 99.4 | 0 | 0 | 0 | 34893 | 18736 | 0 |
| Tragopogonsaponin M | C51 H76 O16 | 944.5144 | 97.5 | 0 | 0 | 0 | 62745 | 103889 | 276362 |
| Basellasaponin D | C47 H68 O22 | 984.4232 | 100 | 0 | 0 | 0 | 53103 | 0 | 0 |
| Cyclopassifloside VII | C37 H62 O13 | 714.4198 | 100 | 0 | 0 | 0 | 38729 | 259458 | 0 |
| 28-Glucosyl-30-methyl-3b,23-dihydroxy-12-oleanene-28,30-dioate 3-[arabinosyl-(1->3)-glucuronide] | C48 H74 O21 | 986.4701 | 98.4 | 0 | 0 | 0 | 54806 | 0 | 0 |
| Tarasaponin I | C47 H74 O18 | 926.4875 | 100 | 0 | 0 | 0 | 23206 | 0 | 52806 |
| 28-Glucosyloleanolic acid 3-[rhamnosyl-(1->2)-galactosyl-(1->3)-glucuronide] | C54 H84 O24 | 1116.5339 | 100 | 0 | 0 | 0 | 32445 | 0 | 0 |
| Cyclopassifloside III | C43 H72 O16 | 844.4839 | 99.6 | 0 | 0 | 0 | 11343 | 71894 | 116431 |
| Kudzusaponin SA2 | C47 H76 O19 | 944.4967 | 100 | 0 | 0 | 0 | 31350 | 0 | 0 |
| Lablabsaponin I | C54 H82 O22 | 1082.5273 | 100 | 0 | 0 | 0 | 51296 | 0 | 262679 |
| Fasciculol C | C30 H52 O6 | 508.3772 | 100 | 0 | 0 | 0 | 154753 | 150463 | 42847 |
| Tuberoside | C34 H56 O8 | 592.3954 | 100 | 0 | 0 | 0 | 103306 | 112157 | 0 |
| Yayoisaponin C | C51 H84 O25 | 1096.5291 | 100 | 0 | 0 | 0 | 82817 | 0 | 0 |
| Ampeloside Bs1 | C45 H74 O20 | 934.4767 | 100 | 0 | 0 | 0 | 44668 | 0 | 0 |
| Balagyptin | C39 H64 O16 | 788.4194 | 97.5 | 0 | 0 | 0 | 57321 | 0 | 0 |
| 1alpha,3beta,22R-Trihydroxyergosta-5,24E-dien-26-oic acid 3-O-b-D-glucoside 26-O-[b-D-glucosyl-(1->2)-b-D-glucosyl] ester | C46 H74 O20 | 946.475 | 100 | 0 | 0 | 0 | 19082 | 0 | 0 |
| Cyclopassifloic acid E | C31 H52 O8 | 552.368 | 96.5 | 0 | 0 | 0 | 97010 | 335832 | 880382 |
| Cucurbitacin C | C32 H48 O8 | 560.3344 | 100 | 0 | 0 | 0 | 115055 | 0 | 0 |
| Corchorusoside A | C35 H54 O14 | 698.3521 | 100 | 0 | 0 | 0 | 45519 | 53671 | 0 |
| Torvoside G | C34 H56 O9 | 608.3882 | 97.4 | 0 | 0 | 0 | 129324 | 0 | 0 |
| makisterone B | C28 H46 O7 | 494.321 | 95.3 | 0 | 0 | 0 | 57737 | 0 | 0 |
| Contignasterol | C29 H48 O7 | 508.338 | 100 | 0 | 0 | 0 | 542281 | 155682 | 0 |
| Brassinolide | C28 H48 O6 | 480.3437 | 100 | 0 | 0 | 0 | 52480 | 0 | 0 |
| Ergosterol peroxide | C28 H44 O3 | 428.329 | 96.8 | 0 | 0 | 0 | 48466 | 0 | 0 |
| Equilin | C18 H20 O2 | 268.1455 | 100 | 0 | 0 | 0 | 207041 | 0 | 0 |
| Yiamoloside B | C43 H68 O15 | 824.4573 | 100 | 0 | 0 | 0 | 22484 | 0 | 0 |
| Cymarin | C30 H44 O9 | 548.2953 | 100 | 0 | 0 | 0 | 108535 | 345969 | 673701 |
| Lucidenic acid B | C27 H38 O7 | 474.2611 | 96.5 | 0 | 0 | 0 | 0 | 66063 | 0 |
| hellebrin | C36 H52 O15 | 724.3212 | 100 | 0 | 0 | 0 | 0 | 35392 | 0 |
| Notoginsenoside R10 | C30 H50 O9 | 554.3431 | 96.7 | 0 | 0 | 0 | 0 | 87177 | 0 |
| Bruceoside A | C32 H42 O16 | 682.2514 | 96.8 | 0 | 0 | 0 | 0 | 33033 | 0 |
| Musaroside | C30 H44 O10 | 564.2906 | 100 | 0 | 0 | 0 | 0 | 141774 | 34581 |
| Melilotoside D | C52 H86 O20 | 1030.571 | 100 | 0 | 0 | 0 | 0 | 79148 | 0 |
| 25-Hydroxy-24-epi-brassinolide | C28 H48 O7 | 496.3389 | 100 | 0 | 0 | 0 | 0 | 308057 | 0 |
| Soyasapogenol B 24-O-b-D-glucoside | C36 H60 O8 | 620.4281 | 100 | 0 | 0 | 0 | 0 | 330317 | 0 |
| Elatoside K | C53 H84 O23 | 1088.5403 | 100 | 0 | 0 | 0 | 0 | 160750 | 43637 |
| Cynarasaponin D | C47 H74 O19 | 942.4799 | 96.8 | 0 | 0 | 0 | 0 | 13843 | 26847 |
| Scopoloside I | C45 H72 O20 | 932.4628 | 97 | 0 | 0 | 0 | 0 | 36311 | 0 |
| Medicoside J | C52 H82 O23 | 1074.525 | 100 | 0 | 0 | 0 | 0 | 33259 | 0 |
| 2-Hydroxyestradiol | C18 H24 O3 | 288.1729 | 100 | 0 | 0 | 0 | 0 | 43296 | 0 |
| Licoricesaponin A3 | C48 H72 O21 | 984.4557 | 96.3 | 0 | 0 | 0 | 0 | 24428 | 0 |
| Polyporusterone F | C28 H46 O5 | 462.3345 | 95.8 | 0 | 0 | 0 | 0 | 256240 | 0 |
| Pregna-5,16,20-triene-3beta,20-diol diacetate | C25 H34 O4 | 398.2451 | 100 | 0 | 0 | 0 | 0 | 387647 | 0 |
| Sarmentoloside | C29 H44 O11 | 568.2892 | 97.2 | 0 | 0 | 0 | 0 | 185342 | 0 |
| 19-Norandrostenedione | C18 H24 O2 | 272.1783 | 97.2 | 0 | 0 | 0 | 0 | 37187 | 0 |
| Eplerenone | C24 H30 O6 | 414.2032 | 100 | 0 | 0 | 0 | 0 | 168319 | 0 |
| Lucidenic acid D2 | C29 H38 O8 | 514.2563 | 100 | 0 | 0 | 0 | 0 | 253310 | 0 |
| A-Norpregn-3(5)-ene-2,20-dione | C20 H28 O2 | 300.2081 | 100 | 0 | 0 | 0 | 0 | 34520 | 0 |
| Campestanol | C28 H50 O | 402.3831 | 100 | 0 | 0 | 0 | 0 | 197899 | 0 |
| Cucurbitacin E | C32 H44 O8 | 556.3041 | 100 | 0 | 0 | 0 | 0 | 332718 | 502283 |
| 3beta-Acetoxy-11alpha-methoxy-12-ursen-28-oic acid | C34 H54 O5 | 542.3948 | 98 | 0 | 0 | 0 | 0 | 77093 | 0 |
| (3beta,5alpha,6beta,9alpha,22E,24R)-23-Methylergosta-7,22-diene-3,5,6,9-tetrol | C29 H48 O4 | 460.3556 | 100 | 0 | 0 | 0 | 0 | 165732 | 81770 |
| (2alpha,3alpha,5alpha,22R,23R)-2,3,22,23-Tetrahydroxy-25-methylergost-24(28)en-6-one | C29 H48 O5 | 476.3496 | 100 | 0 | 0 | 0 | 0 | 126074 | 0 |
| Typhasterol | C28 H48 O4 | 448.3558 | 100 | 0 | 0 | 0 | 0 | 375183 | 0 |
| 6-Deoxocastasterone | C28 H50 O4 | 450.3712 | 100 | 0 | 0 | 0 | 0 | 376064 | 0 |
| (3beta,5alpha,9alpha,22E,24R)-5,9-Epidioxy-3-hydroxyergosta-7,22-dien-6-one | C28 H42 O4 | 442.3077 | 100 | 0 | 0 | 0 | 0 | 82082 | 0 |
| Schleicherastatin 3 | C29 H50 O3 | 446.3714 | 100 | 0 | 0 | 0 | 0 | 120535 | 0 |
| A-Nor-5alpha-cholestan-2-one | C26 H44 O | 372.3261 | 100 | 0 | 0 | 0 | 0 | 0 | 135022 |
| antioside | C29 H44 O10 | 552.2948 | 97.3 | 0 | 0 | 0 | 0 | 0 | 43224 |
| 25-Hydroxy-cholesterol | C27 H46 O2 | 402.3477 | 95.9 | 0 | 0 | 0 | 0 | 0 | 28752 |
| Neogitogenin 3-[glucosyl-(1->2)-glucosyl-(1->4)-galactoside] | C45 H74 O19 | 918.4811 | 100 | 0 | 0 | 0 | 0 | 0 | 235280 |
| 17beta-Methylestra-1,3,5(10)-trien-3-ol | C19 H26 O | 270.1979 | 100 | 0 | 0 | 0 | 0 | 0 | 14036 |
| Methylprotodioscin | C52 H86 O22 | 1062.559 | 98.4 | 0 | 0 | 0 | 0 | 0 | 15965 |
| 22-Acetylpriverogenin B | C32 H52 O5 | 516.3792 | 100 | 0 | 0 | 0 | 0 | 0 | 89931 |
| delta3,5-Deoxyneotigogenin | C27 H40 O2 | 396.3025 | 98.7 | 0 | 0 | 0 | 0 | 0 | 11156 |
| Ganoderiol I | C31 H50 O5 | 502.3639 | 100 | 0 | 0 | 0 | 0 | 0 | 83035 |
| Methyl 3b-hydroxy-13(18)-oleanen-28-oate | C33 H52 O4 | 512.387 | 100 | 0 | 0 | 0 | 0 | 0 | 235807 |
| 11beta,21-Dihydroxypregn-4-ene-3,20-dione 21-acetate | C23 H32 O5 | 388.2259 | 98.5 | 0 | 0 | 0 | 0 | 0 | 53620 |
| 17alpha-Dihydroequilenin | C18 H20 O2 | 268.1462 | 100 | 0 | 0 | 0 | 0 | 0 | 28687 |
| Lynestrenol | C20 H28 O | 284.2127 | 98 | 0 | 0 | 0 | 0 | 0 | 91846 |
| 17-Hydroxy-3-oxo-19-nor-5alpha,17alpha-pregnane-21-carboxylic acid, gamma-lactone | C21 H30 O3 | 330.2183 | 100 | 0 | 0 | 0 | 0 | 0 | 14913 |
| Sibiricoside A | C57 H94 O28 | 1226.5912 | 99.2 | 0 | 0 | 0 | 0 | 0 | 58565 |
| 27-Nor-5b-cholestane-3a,7a,12a,24,25-pentol | C26 H46 O5 | 438.3335 | 100 | 0 | 0 | 0 | 0 | 0 | 18910 |
| Cannogenol 3-[glucosyl-(1->4)-2,6-dideoxy-xylohexoside] | C35 H54 O13 | 682.3537 | 100 | 0 | 0 | 0 | 0 | 0 | 11424 |
| Clerosterol 3-glucoside | C35 H58 O6 | 574.421 | 98 | 0 | 0 | 0 | 0 | 0 | 61999 |
| Melongoside P | C51 H86 O23 | 1066.5609 | 100 | 0 | 0 | 0 | 0 | 0 | 34556 |
| Testosterone isocaproate | C25 H38 O3 | 386.2833 | 100 | 0 | 0 | 0 | 0 | 0 | 78403 |
| Tuberoside J | C39 H64 O14 | 756.4291 | 100 | 0 | 0 | 0 | 0 | 0 | 156805 |
| Stylisterol C | C28 H46 O3 | 430.3449 | 97.4 | 0 | 0 | 0 | 0 | 0 | 110863 |
| 12-epi-Scalaradial | C27 H40 O4 | 428.2932 | 100 | 0 | 279656 | 0 | 380739 | 0 | 43455 |
| 7a,12a-Dihydroxy-3-oxo-4-cholenoic acid | C24 H36 O5 | 404.2566 | 100 | 0 | 0 | 0 | 0 | 0 | 47790 |
| Acetyljujuboside B | C54 H86 O22 | 1086.5599 | 100 | 0 | 0 | 0 | 0 | 0 | 15656 |
| Cyclopassifloside X | C37 H62 O12 | 698.4266 | 100 | 0 | 0 | 69867 | 0 | 0 | 17915 |
| Shanzhiside | C16 H24 O11 | 392.1283 | 100 | 90555 | 77614 | 0 | 0 | 0 | 0 |
| 7-Epiloganin tetraacetate | C25 H34 O14 | 558.1951 | 98.2 | 19845 | 0 | 0 | 0 | 0 | 0 |
| Schizonepetoside A | C16 H26 O7 | 330.1687 | 97.1 | 264446 | 0 | 0 | 0 | 0 | 0 |
| Picrasin B | C21 H28 O6 | 376.187 | 99.3 | 150006 | 0 | 0 | 0 | 0 | 46284 |
| Nigakilactone B | C22 H32 O6 | 392.221 | 100 | 661990 | 0 | 0 | 0 | 0 | 0 |
| Gibberellin A8-catabolite | C19 H22 O7 | 362.1364 | 98.8 | 69387 | 0 | 0 | 0 | 0 | 0 |
| Annoglabasin C | C23 H34 O6 | 406.2341 | 100 | 1181499 | 435604 | 169269 | 0 | 270155 | 950406 |
| Pleniradin | C15 H20 O4 | 264.1347 | 96.1 | 121857 | 0 | 0 | 0 | 67472 | 0 |
| Ceanothenic acid | C29 H42 O4 | 454.3072 | 100 | 142640 | 0 | 0 | 0 | 0 | 0 |
| Paucin | C23 H32 O10 | 468.1985 | 100 | 85927 | 0 | 0 | 0 | 0 | 0 |
| Lactupicrin | C23 H22 O7 | 410.137 | 100 | 28698 | 0 | 0 | 0 | 0 | 0 |
| Pisumionoside | C19 H32 O9 | 404.2051 | 100 | 55102 | 0 | 0 | 0 | 355405 | 0 |
| Citronellyl beta-sophoroside | C22 H40 O11 | 480.2553 | 100 | 479283 | 63098 | 128541 | 164600 | 205304 | 97240 |
| Glaucarubin | C25 H36 O10 | 496.2287 | 98 | 47145 | 0 | 0 | 0 | 0 | 0 |
| 3-O-Methylniveusin A | C21 H28 O8 | 408.1779 | 97.6 | 119615 | 0 | 0 | 0 | 0 | 46247 |
| Scorzoside | C21 H30 O8 | 410.1931 | 99.1 | 811727 | 30779 | 103314 | 0 | 9772819 | 0 |
| Brusatol | C26 H32 O11 | 520.1956 | 100 | 254652 | 0 | 0 | 0 | 0 | 0 |
| Agnuside | C22 H26 O11 | 466.1469 | 100 | 73096 | 0 | 30234 | 101078 | 0 | 0 |
| Lippioside II | C25 H30 O14 | 554.1645 | 100 | 50388 | 0 | 0 | 0 | 0 | 0 |
| Melleolide H | C24 H30 O7 | 430.1989 | 100 | 87978 | 0 | 0 | 0 | 0 | 0 |
| Kiwiionoside | C19 H34 O9 | 406.2214 | 100 | 120112 | 0 | 0 | 0 | 0 | 0 |
| (3b,4b,11b,14b)-11-Ethoxy-3,4-epoxy-14-hydroxy-12-cyathen-15-al 14-xyloside | C27 H42 O8 | 494.2883 | 100 | 209886 | 32510 | 57258 | 0 | 113826 | 0 |
| Cinncassiol D2 glucoside | C26 H42 O11 | 530.2731 | 100 | 68010 | 0 | 96919 | 0 | 0 | 11491 |
| (3S,5R,6R,7E)-3,5,6-Trihydroxy-7-megastigmen-9-one | C13 H22 O4 | 242.1529 | 100 | 122632 | 0 | 0 | 233389 | 240257 | 0 |
| Asebotoxin II | C23 H36 O6 | 408.2503 | 100 | 4821580 | 3439834 | 0 | 0 | 0 | 0 |
| 6b-Angeloyl-3b,8b,9b-trihydroxy-7(11)-eremophilen-12,8-olide | C20 H28 O7 | 380.1828 | 98.6 | 95165 | 0 | 0 | 0 | 0 | 0 |
| Santamarin | C15 H20 O3 | 248.1406 | 97.8 | 182835 | 0 | 0 | 0 | 152213 | 34979 |
| Glycinoeclepin C | C29 H38 O8 | 514.257 | 100 | 578838 | 0 | 22637 | 0 | 0 | 0 |
| Lactapiperanol D | C18 H28 O5 | 324.1948 | 100 | 105239 | 0 | 0 | 0 | 906831 | 0 |
| Asacoumarin A | C24 H30 O5 | 398.2084 | 100 | 76970 | 0 | 27283 | 0 | 0 | 0 |
| Perilloside B | C16 H24 O7 | 328.153 | 100 | 85034 | 0 | 136914 | 97575 | 0 | 0 |
| Annosquamosin B | C19 H32 O3 | 308.2354 | 95 | 166963 | 0 | 0 | 0 | 177664 | 97663 |
| sclareol | C20 H36 O2 | 308.2716 | 100 | 22026 | 0 | 0 | 0 | 0 | 0 |
| Dehydrocostus lactone | C15 H18 O2 | 230.1316 | 100 | 150305 | 0 | 0 | 0 | 0 | 0 |
| Multiradiatin | C20 H22 O6 | 358.1426 | 100 | 67990 | 0 | 0 | 0 | 0 | 0 |
| Schizonepetoside E | C16 H28 O8 | 348.1795 | 97.6 | 94818 | 78225 | 0 | 0 | 0 | 52794 |
| Lamioside | C18 H28 O11 | 420.162 | 100 | 2556795 | 59909 | 28530 | 215248 | 0 | 0 |
| Deoxyloganin tetraacetate | C25 H34 O13 | 542.1982 | 100 | 271132 | 0 | 0 | 0 | 0 | 0 |
| Sonchuionoside C | C19 H30 O8 | 386.1948 | 100 | 215537 | 0 | 0 | 0 | 0 | 0 |
| Cotylenin F | C33 H54 O11 | 626.3677 | 100 | 1764203 | 52075 | 0 | 84202 | 498025 | 135529 |
| Genipin 1-beta-gentiobioside | C23 H34 O15 | 550.1924 | 100 | 32070 | 0 | 0 | 0 | 0 | 0 |
| Rehmaionoside B | C19 H34 O8 | 390.2259 | 100 | 342286 | 0 | 0 | 63644 | 838162 | 103534 |
| Iridotrial glucoside | C16 H24 O8 | 344.1459 | 98 | 29574 | 0 | 31538 | 0 | 0 | 0 |
| 11-Hydroxyiridodial glucoside pentaacetate | C26 H36 O13 | 556.2146 | 100 | 53308 | 0 | 0 | 0 | 0 | 0 |
| Iridodial glucoside tetraacetate | C24 H34 O11 | 498.2089 | 100 | 786077 | 190964 | 65953 | 0 | 252503 | 0 |
| Picrasin G | C21 H28 O7 | 392.1822 | 100 | 71509 | 0 | 0 | 0 | 0 | 0 |
| Viguiestenin | C21 H28 O7 | 392.1817 | 97.1 | 0 | 56162 | 82993 | 0 | 0 | 0 |
| Vernoflexuoside | C21 H28 O8 | 408.1773 | 100 | 0 | 249382 | 401267 | 0 | 0 | 79296 |
| Tocopheronic acid | C16 H22 O5 | 294.1453 | 100 | 0 | 186924 | 0 | 0 | 0 | 0 |
| Taraxacolide 1-O-b-D-glucopyranoside | C21 H32 O9 | 428.2025 | 96.8 | 0 | 24881 | 0 | 0 | 0 | 0 |
| Sugeonyl acetate | C17 H24 O3 | 276.1725 | 100 | 0 | 47661 | 0 | 206006 | 50027 | 41535 |
| Sergeolide | C25 H28 O11 | 504.1635 | 95.7 | 0 | 22238 | 0 | 0 | 0 | 0 |
| plaunotol | C20 H34 O2 | 306.2563 | 98.1 | 0 | 136728 | 0 | 0 | 0 | 0 |
| Pimelea factor P2 | C37 H50 O9 | 638.3456 | 100 | 0 | 55411 | 0 | 0 | 0 | 0 |
| Patrinoside | C21 H34 O11 | 462.2085 | 100 | 0 | 174600 | 0 | 0 | 0 | 0 |
| Nigakilactone N | C21 H30 O7 | 394.1972 | 97 | 0 | 54377 | 0 | 0 | 0 | 0 |
| Nigakilactone H | C22 H32 O8 | 424.2087 | 100 | 0 | 6445276 | 6154328 | 0 | 0 | 0 |
| Microlenin | C29 H34 O7 | 494.2291 | 100 | 0 | 47875 | 0 | 0 | 0 | 0 |
| Lyciumoside IX | C35 H56 O15 | 716.3599 | 100 | 0 | 29066 | 0 | 0 | 0 | 0 |
| Hovenidulcigenin B | C32 H50 O7 | 546.355 | 99.4 | 0 | 244686 | 0 | 0 | 0 | 0 |
| Erinacine P | C27 H40 O8 | 492.2723 | 100 | 0 | 9753 | 0 | 0 | 291596 | 0 |
| Cinncassiol E | C20 H30 O8 | 398.1922 | 100 | 0 | 68569 | 0 | 0 | 0 | 0 |
| Cinncassiol D4 2-glucoside | C26 H42 O10 | 514.2767 | 100 | 0 | 99062 | 63922 | 0 | 0 | 0 |
| Cinncassiol C3 | C20 H30 O7 | 382.1972 | 99.7 | 0 | 478212 | 0 | 0 | 0 | 0 |
| Cincassiol B | C20 H32 O8 | 400.2098 | 100 | 0 | 30973 | 0 | 0 | 0 | 0 |
| Cascarillin | C22 H32 O7 | 408.2132 | 99.6 | 0 | 63296 | 59267 | 0 | 0 | 0 |
| Callystatin A | C29 H44 O4 | 456.3218 | 100 | 0 | 56456 | 0 | 0 | 0 | 50301 |
| beta-Micropteroxanthin | C27 H40 O2 | 396.3034 | 100 | 0 | 416888 | 469927 | 0 | 0 | 0 |
| Azafrin | C27 H38 O4 | 426.2778 | 100 | 0 | 85548 | 1647850 | 132292 | 305795 | 0 |
| Alkhanol | C15 H22 O4 | 266.1506 | 100 | 0 | 45145 | 0 | 0 | 1635690 | 0 |
| Abscisic alcohol 11-glucoside | C21 H32 O8 | 412.2079 | 100 | 0 | 105093 | 139393 | 0 | 173921 | 44359 |
| 9alpha-(3-Methyl-2E-butenoyloxy)-4S-hydroxy-10(14)-oplopen-3-one 4-acetate | C22 H32 O5 | 376.2231 | 100 | 0 | 140666 | 0 | 0 | 158722 | 0 |
| 7,8-Dehydro-beta-micropteroxanthin | C27 H38 O2 | 394.2883 | 100 | 0 | 1635122 | 1110554 | 82533 | 599582 | 0 |
| Thymol | C10 H14 O | 150.1049 | 98.4 | 0 | 0 | 28797 | 0 | 0 | 0 |
| Sugetriol triacetate | C21 H30 O6 | 378.2046 | 100 | 0 | 0 | 60663 | 455819 | 95666 | 31530 |
| Secologanin | C17 H24 O10 | 388.1353 | 100 | 0 | 0 | 44657 | 0 | 0 | 0 |
| Picrasin C | C23 H34 O7 | 422.2286 | 97.9 | 0 | 0 | 77792 | 0 | 0 | 0 |
| Oxepahyperforin | C35 H52 O5 | 552.3825 | 100 | 0 | 0 | 5356336 | 0 | 0 | 0 |
| Ophiobolin A | C25 H36 O4 | 400.2595 | 98.7 | 0 | 0 | 36177 | 0 | 0 | 0 |
| Michelenolide | C15 H20 O4 | 264.1349 | 98.8 | 0 | 0 | 75859 | 0 | 0 | 0 |
| Mexicanin I | C15 H18 O4 | 262.1196 | 100 | 0 | 0 | 60949 | 0 | 0 | 0 |
| Methyl (3b,11x)-3-Hydroxy-8-oxo-6-eremophilen-12-oate | C16 H24 O4 | 280.1665 | 100 | 0 | 0 | 30842 | 0 | 0 | 0 |
| Melleolide M | C23 H29 Cl O7 | 452.1607 | 100 | 0 | 0 | 193398 | 0 | 0 | 0 |
| Kinoprene | C18 H28 O2 | 276.2092 | 100 | 0 | 0 | 309322 | 0 | 72408 | 0 |
| Ipolamiide | C17 H26 O11 | 406.1444 | 96 | 0 | 0 | 29176 | 197518 | 0 | 0 |
| Goshonoside F3 | C32 H52 O13 | 644.3389 | 100 | 0 | 0 | 34487 | 0 | 0 | 0 |
| Ginsenoyne L | C32 H46 O2 | 462.3476 | 99.2 | 0 | 0 | 44005 | 0 | 0 | 0 |
| Gibberellin A86 | C19 H24 O8 | 380.1459 | 98 | 0 | 0 | 39365 | 0 | 0 | 0 |
| Gibberellin A8 | C19 H24 O7 | 364.1503 | 97.2 | 0 | 0 | 41629 | 0 | 0 | 0 |
| Gardenoside | C17 H24 O11 | 404.1303 | 100 | 0 | 0 | 42346 | 0 | 0 | 0 |
| Eleganin | C22 H26 O9 | 434.1596 | 98.8 | 0 | 0 | 17865 | 0 | 0 | 0 |
| Condelphine | C25 H39 N O6 | 449.2761 | 100 | 0 | 0 | 38525 | 0 | 0 | 0 |
| Cinnzeylanol | C20 H32 O7 | 384.2137 | 100 | 0 | 0 | 91143 | 0 | 0 | 0 |
| Cavipetin C | C24 H36 O4 | 388.2613 | 100 | 0 | 0 | 357367 | 291198 | 0 | 0 |
| Boviquinone 4 | C26 H36 O4 | 412.2595 | 100 | 0 | 0 | 6306910 | 0 | 0 | 0 |
| Apo-10'-violaxanthal | C27 H36 O3 | 408.2666 | 100 | 0 | 0 | 124789 | 93609 | 0 | 0 |
| 7,8-Dehydroastaxanthianthin | C40 H50 O4 | 594.373 | 100 | 0 | 0 | 277443 | 0 | 0 | 0 |
| 4-Methoxy-3-geranylgeranyl-1,2-dihydroxybenzene | C27 H40 O3 | 412.2984 | 100 | 0 | 0 | 910203 | 0 | 0 | 0 |
| 33-Deoxy-33-hydroperoxyfurohyperforin | C35 H52 O6 | 568.377 | 100 | 0 | 0 | 1424683 | 0 | 0 | 0 |
| 2,9-Bis(3-methyl-2E-pentenoyl)-2b,9a-dihydroxy-4Z,10(14)-oplopadien-3-one | C27 H38 O5 | 442.2723 | 100 | 0 | 0 | 51841 | 149003 | 167941 | 22077 |
| (4S,5S)-(+)-Germacrone 4,5-epoxide | C15 H22 O2 | 234.1624 | 100 | 0 | 0 | 73125 | 68566 | 54518 | 0 |
| Clerodin | C24 H34 O7 | 434.2309 | 100 | 0 | 0 | 0 | 98501 | 0 | 0 |
| Rhodojaponin IV | C24 H38 O8 | 454.2554 | 99.8 | 0 | 0 | 0 | 39234 | 0 | 0 |
| Baliospermin | C32 H50 O8 | 562.3496 | 100 | 0 | 0 | 0 | 341779 | 0 | 0 |
| Deoxyloganic acid tetraacetate | C24 H32 O13 | 528.1805 | 98.3 | 0 | 0 | 0 | 8846 | 0 | 0 |
| 3-Hydroxy-beta-ionol 3-[glucosyl-(1->6)-glucoside] | C25 H42 O12 | 534.2659 | 100 | 1459484 | 0 | 0 | 583834 | 57507 | 317421 |
| 25-Cinnamoyl-vulgaroside | C34 H46 O7 | 566.3267 | 100 | 0 | 0 | 0 | 63380 | 0 | 0 |
| 5,8-Epoxy-5,8-dihydro-10'-apo-b,y-carotene-3,10'-diol | C27 H38 O3 | 410.2809 | 97.1 | 0 | 0 | 0 | 103860 | 73642 | 0 |
| 3-Hydroxy-10'-apo-b,y-carotenal | C27 H36 O2 | 392.2716 | 100 | 0 | 0 | 0 | 159776 | 403619 | 0 |
| Laserpitin | C25 H38 O7 | 450.2603 | 100 | 0 | 0 | 0 | 171976 | 0 | 0 |
| Cinncassiol A 19-glucoside | C26 H40 O12 | 544.2502 | 96.1 | 0 | 0 | 0 | 71235 | 0 | 0 |
| Stevioside | C38 H60 O18 | 804.3774 | 96.2 | 0 | 0 | 0 | 0 | 75430 | 0 |
| Samaderin A | C18 H18 O6 | 330.1128 | 99.8 | 0 | 0 | 0 | 0 | 24340 | 0 |
| (1R*,2R*,4R*,8S*)-p-Menthane-1,2,8,9-tetrol 9-glucoside | C16 H30 O9 | 366.1894 | 100 | 0 | 0 | 0 | 0 | 1057561 | 0 |
| (1alpha,2alpha,4betaH,6alpha,8R)-p-Menthane-2,6,8,9-tetrol | C10 H20 O4 | 204.1361 | 100 | 0 | 0 | 0 | 0 | 109041 | 0 |
| (1S,2R,4R,8S)-p-Menthane-2,8,9-triol 2-glucoside | C16 H30 O8 | 350.1945 | 98.2 | 0 | 0 | 0 | 0 | 221825 | 0 |
| (1R,2R,4S)-p-Menthane-1,2,8-triol | C10 H20 O3 | 188.1417 | 100 | 0 | 0 | 0 | 0 | 150042 | 0 |
| Lyciumoside IV | C38 H64 O16 | 776.4194 | 97.1 | 0 | 0 | 0 | 0 | 91200 | 0 |
| Veranisatin A | C16 H22 O8 | 342.1309 | 96.6 | 0 | 0 | 0 | 0 | 65915 | 0 |
| 10-Deacetyl-2-debenzoylbaccatin III | C22 H32 O9 | 440.2029 | 100 | 0 | 0 | 0 | 0 | 70377 | 81947 |
| Harpagoside | C24 H30 O11 | 494.1787 | 96.1 | 0 | 0 | 0 | 0 | 39409 | 0 |
| Erinacine C | C25 H38 O6 | 434.2644 | 100 | 0 | 0 | 0 | 0 | 562804 | 0 |
| (6S,9R)-Vomifoliol | C13 H20 O3 | 224.1415 | 100 | 176974 | 0 | 0 | 0 | 1817220 | 280277 |
| Kikkanol A | C15 H26 O3 | 254.189 | 100 | 0 | 0 | 0 | 0 | 211178 | 77912 |
| Lactaronecatorin A | C15 H22 O3 | 250.1573 | 95.4 | 0 | 0 | 0 | 0 | 232717 | 48484 |
| Nigakilactone F | C22 H32 O7 | 408.2134 | 100 | 0 | 0 | 0 | 0 | 360495 | 0 |
| Monomenthyl succinate | C14 H24 O4 | 256.1681 | 100 | 0 | 0 | 0 | 0 | 81321 | 0 |
| 19-Hydroxycinnzeylanol 19-glucoside | C26 H42 O13 | 562.2631 | 100 | 0 | 0 | 0 | 0 | 476386 | 0 |
| Palustric acid | C20 H30 O2 | 302.2244 | 100 | 0 | 0 | 0 | 0 | 119832 | 0 |
| Hericenone C | C35 H54 O6 | 570.3912 | 95.7 | 0 | 0 | 0 | 0 | 169199 | 0 |
| Erioflorin acetate | C21 H26 O7 | 390.1701 | 97.6 | 0 | 0 | 0 | 0 | 0 | 24409 |
| Erinacine B | C25 H36 O6 | 432.2509 | 100 | 0 | 0 | 0 | 0 | 0 | 42878 |
| Columbaridione | C20 H24 O5 | 344.1612 | 100 | 0 | 0 | 0 | 0 | 0 | 143180 |
| 2-(Methoxycarbonyl)-5-methyl-2,4-bis(3-methyl-2-butenyl)-6-(2-methyl-1-oxopropyl)-5-(4-methyl-3-pentenyl)cyclohexanone | C29 H46 O4 | 458.3382 | 100 | 0 | 0 | 0 | 0 | 0 | 120397 |
| Cynaroside A | C21 H32 O10 | 444.1996 | 100 | 0 | 0 | 0 | 0 | 0 | 16904 |
| Goshonoside F5 | C32 H54 O13 | 646.3584 | 100 | 0 | 0 | 0 | 0 | 0 | 8738 |
| Miltirone | C19 H22 O2 | 282.1625 | 100 | 0 | 0 | 0 | 0 | 0 | 35633 |
| Ophiobolin F | C25 H42 O | 358.3218 | 100 | 0 | 0 | 0 | 0 | 0 | 78876 |
| (13R,14R)-7-Labdene-13,14,15-triol | C20 H36 O3 | 324.2667 | 100 | 0 | 0 | 0 | 0 | 0 | 76355 |
| 3''-Hydroxy-geranylhydroquinone | C16 H22 O3 | 262.1544 | 97.9 | 0 | 0 | 0 | 0 | 0 | 76389 |
| Lappaol C | C30 H34 O10 | 554.2182 | 100 | 26615 | 0 | 0 | 0 | 0 | 0 |
| Sphagnum acid | C11 H10 O5 | 222.053 | 100 | 199735 | 0 | 0 | 0 | 0 | 0 |
| 4-O-Caffeoylshikimic acid | C16 H16 O8 | 336.0832 | 100 | 44320 | 0 | 0 | 0 | 90057 | 0 |
| Gravolenic acid | C14 H16 O6 | 280.0934 | 100 | 124216 | 0 | 0 | 0 | 0 | 0 |
| [6]-Dehydrogingerdione | C17 H22 O4 | 290.1527 | 99.9 | 24723 | 0 | 0 | 0 | 0 | 0 |
| Osthenol-7-O-beta-D-gentiobioside | C26 H34 O13 | 554.1957 | 100 | 43690 | 0 | 0 | 0 | 0 | 0 |
| Malonyldaidzin | C24 H22 O12 | 502.1106 | 100 | 12826 | 0 | 0 | 0 | 0 | 0 |
| Scopoletin | C10 H8 O4 | 192.0427 | 100 | 53248 | 0 | 0 | 0 | 0 | 0 |
| Marmesin galactoside | C20 H24 O9 | 408.1409 | 98.1 | 38102 | 0 | 0 | 0 | 0 | 0 |
| Herniarin | C10 H8 O3 | 176.0481 | 100 | 133507 | 491223 | 97736 | 111712 | 59657 | 0 |
| (R)-Rutaretin 1'-(6''-sinapoylglucoside) | C31 H34 O14 | 630.1914 | 100 | 23286 | 0 | 0 | 0 | 0 | 0 |
| Swertianolin | C20 H20 O11 | 436.0992 | 100 | 32627 | 0 | 0 | 0 | 0 | 0 |
| Gallocatechin-4beta-ol | C15 H14 O8 | 322.0679 | 99.3 | 24097 | 0 | 0 | 0 | 39984 | 0 |
| Eriodictyol 7-(6-trans-p-coumaroylglucoside) | C30 H28 O13 | 596.1484 | 96.7 | 25505 | 0 | 0 | 0 | 0 | 0 |
| 7-Hydroxy-3-methoxy-1-primeverosyloxyxanthone | C25 H28 O14 | 552.1478 | 100 | 44738 | 0 | 0 | 0 | 0 | 0 |
| Procyanidin B2 | C30 H26 O12 | 578.1424 | 100 | 27330 | 0 | 0 | 0 | 0 | 0 |
| Aromadendrin 3,7-diglucoside | C27 H32 O16 | 612.1689 | 95.8 | 49384 | 0 | 0 | 0 | 22944 | 0 |
| Tetramethylquercetin 3-rutinoside | C31 H38 O16 | 666.2145 | 100 | 135650 | 0 | 0 | 0 | 0 | 0 |
| Isoswertisin 2''-O-rhamnoside | C28 H32 O14 | 592.1794 | 100 | 46911 | 0 | 0 | 0 | 0 | 0 |
| 2''-(6''-p-Coumaroylglucosyl)quercitrin | C36 H36 O18 | 756.1888 | 100 | 29532 | 0 | 0 | 0 | 54862 | 0 |
| Flavonol 3-O-[alpha-L-rhamnosyl-(1->6)-beta-D-glucoside] | C27 H30 O12 | 546.1725 | 100 | 3414350 | 0 | 0 | 0 | 0 | 0 |
| 2'',6''-Di-O-acetylononin | C26 H26 O11 | 514.1483 | 100 | 827261 | 132589 | 89129 | 0 | 0 | 0 |
| Gambiriin A2 | C30 H28 O12 | 580.155 | 97.9 | 108510 | 0 | 0 | 0 | 0 | 0 |
| Arctiin | C27 H34 O11 | 534.2107 | 100 | 231721 | 0 | 0 | 0 | 0 | 0 |
| 1-Acetoxypinoresinol | C22 H24 O8 | 416.1486 | 100 | 55577 | 0 | 0 | 0 | 0 | 0 |
| Dalpanin | C26 H30 O12 | 534.1745 | 100 | 99411 | 0 | 0 | 0 | 44389 | 0 |
| 5,7-Dihydroxy-3',4'-dimethoxy-8-(3-hydroxy-3-methylbutyl)-isoflavone 7-glucoside | C28 H34 O12 | 562.2066 | 100 | 157738 | 14131 | 0 | 0 | 0 | 0 |
| Kanzonol M | C23 H26 O6 | 398.1717 | 100 | 294348 | 0 | 0 | 0 | 0 | 0 |
| Citrusin A | C26 H34 O12 | 538.2061 | 100 | 628363 | 109314 | 0 | 0 | 0 | 0 |
| Phenethyl rutinoside | C20 H30 O10 | 430.1832 | 95.8 | 48046 | 0 | 0 | 0 | 55676 | 0 |
| Dihydrosinapic acid | C11 H14 O5 | 226.0847 | 100 | 27226 | 0 | 0 | 0 | 0 | 0 |
| Norswertianolin | C19 H18 O11 | 422.084 | 100 | 28093 | 0 | 0 | 0 | 0 | 0 |
| Trachelogenin | C21 H24 O7 | 388.1512 | 100 | 0 | 64522 | 0 | 0 | 0 | 0 |
| Mahaleboside | C15 H16 O8 | 324.0831 | 100 | 0 | 45471 | 0 | 0 | 0 | 0 |
| Licoricidin | C26 H32 O5 | 424.2231 | 100 | 0 | 306472 | 0 | 0 | 0 | 0 |
| Leonuriside A | C14 H20 O9 | 332.1074 | 100 | 265532 | 25776 | 0 | 0 | 0 | 0 |
| Kelampayoside A | C20 H30 O13 | 478.1696 | 100 | 25231 | 15887 | 0 | 0 | 0 | 0 |
| Kanzonol K | C26 H28 O6 | 436.1871 | 100 | 0 | 428143 | 418982 | 0 | 0 | 0 |
| Grandidentatin | C21 H28 O9 | 424.173 | 100 | 1805513 | 34962 | 43893 | 0 | 0 | 0 |
| Gomisin L1 methyl ether | C23 H28 O6 | 400.1867 | 100 | 0 | 213081 | 0 | 0 | 0 | 0 |
| Cyanidin 3-O-3'',6''-O-dimalonylglucoside | C27 H25 O17 | 621.1103 | 100 | 0 | 13744 | 0 | 0 | 0 | 0 |
| Camellianin A | C29 H32 O15 | 620.1723 | 100 | 0 | 22069 | 0 | 0 | 0 | 0 |
| Austalide L | C25 H32 O6 | 428.2185 | 100 | 0 | 63374 | 0 | 0 | 443846 | 0 |
| Arbutin | C12 H16 O7 | 272.0882 | 100 | 63807 | 70823 | 53322 | 0 | 0 | 0 |
| Acrovestone | C32 H42 O8 | 554.286 | 100 | 0 | 109097 | 18374 | 0 | 0 | 0 |
| 5',8-Dihydroxy-3',4',7-trimethoxyflavan | C18 H20 O6 | 332.1238 | 100 | 0 | 63581 | 0 | 0 | 0 | 0 |
| 4-Dodecylphenol | C18 H30 O | 262.2304 | 100 | 0 | 195646 | 0 | 0 | 0 | 0 |
| 3-O-Caffeoyl-4-O-methylquinic acid | C17 H20 O9 | 368.1117 | 100 | 0 | 146485 | 22281 | 0 | 0 | 0 |
| 2,2-Dibutyl-3-(4-methoxyphenyl)-4-methyl-2H-1-benzopyran-7-ol acetate | C27 H34 O4 | 422.2466 | 100 | 0 | 44951 | 0 | 0 | 0 | 0 |
| (+)-Eudesmin | C22 H26 O6 | 386.1742 | 100 | 0 | 80585 | 124270 | 0 | 0 | 25322 |
| Tigloylgomicin H | C28 H36 O8 | 500.239 | 98.7 | 0 | 0 | 334968 | 0 | 0 | 0 |
| Ssioriside | C27 H38 O12 | 554.2361 | 97.2 | 0 | 0 | 26278 | 0 | 0 | 0 |
| Podorhizol beta-D-glucoside | C28 H34 O13 | 578.1973 | 95.4 | 3632139 | 0 | 11531 | 0 | 0 | 0 |
| Podophyllotoxone | C22 H20 O8 | 412.1144 | 97.5 | 0 | 0 | 33978 | 0 | 0 | 0 |
| Phyllanthin | C24 H34 O6 | 418.2352 | 97.1 | 0 | 0 | 75565 | 0 | 0 | 19578 |
| p-Hydroxycinnamaldehyde | C9 H8 O2 | 148.0517 | 100 | 0 | 0 | 92191 | 0 | 0 | 0 |
| Petunidin | C16 H13 O7 | 317.0664 | 100 | 0 | 0 | 11305 | 0 | 0 | 0 |
| Oxyacanthine | C37 H40 N2 O6 | 608.2887 | 100 | 0 | 0 | 165787 | 0 | 0 | 0 |
| Moracin N | C19 H18 O4 | 310.1188 | 96.6 | 0 | 0 | 228121 | 0 | 0 | 0 |
| Melitric acid B | C27 H20 O11 | 520.102 | 100 | 0 | 0 | 88908 | 0 | 0 | 0 |
| Maysin 3'-methyl ether | C28 H30 O14 | 590.1651 | 96 | 0 | 0 | 12020 | 0 | 0 | 0 |
| Kurarinol | C26 H32 O7 | 456.2133 | 100 | 0 | 0 | 135490 | 0 | 0 | 0 |
| Kanzonol J | C26 H30 O5 | 422.2063 | 100 | 0 | 0 | 33927 | 0 | 0 | 0 |
| Feruloylputrescine | C14 H20 N2 O3 | 264.1478 | 100 | 0 | 0 | 347599 | 0 | 0 | 0 |
| Eriodictyol 7-(6-galloylglucoside) | C28 H26 O15 | 602.1247 | 100 | 0 | 0 | 68941 | 0 | 2790340 | 0 |
| Dihydroferuloylglycine | C12 H15 N O5 | 253.0935 | 99.9 | 0 | 0 | 49315 | 0 | 0 | 0 |
| Dihydro-3-coumaric acid | C9 H10 O3 | 166.0631 | 100 | 0 | 0 | 65027 | 0 | 0 | 0 |
| Cyanin | C27 H31 O16 | 611.1612 | 100 | 0 | 0 | 16556 | 0 | 0 | 0 |
| 8-Hydroxypinoresinol 4-glucoside | C26 H32 O12 | 536.1888 | 96.5 | 0 | 0 | 9287 | 0 | 0 | 0 |
| 4-Amino-4-deoxychorismic acid | C10 H11 N O5 | 225.0627 | 100 | 0 | 0 | 83984 | 0 | 0 | 0 |
| 4',6'-Dihydroxy-2'-methoxyacetophenone 6'-glucoside | C15 H20 O9 | 344.1093 | 100 | 0 | 0 | 43429 | 0 | 0 | 0 |
| 3,5-Dicaffeoyl-4-succinoylquinic acid | C29 H28 O15 | 616.1407 | 100 | 0 | 0 | 37648 | 0 | 0 | 0 |
| 3,4,5-Trimethoxyphenyl glucoside | C15 H22 O9 | 346.1266 | 100 | 300738 | 31339 | 24321 | 0 | 0 | 43599 |
| (-)-Sativan | C17 H18 O4 | 286.1178 | 97.8 | 0 | 0 | 56094 | 0 | 0 | 90897 |
| Kaempferol 7-galactoside 3-rutinoside | C33 H40 O20 | 756.2106 | 100 | 0 | 0 | 0 | 22126 | 0 | 13943 |
| Flavonol 3-O-beta-D-glucosyl-(1->2)-beta-D-glucoside | C27 H30 O13 | 562.163 | 97.1 | 0 | 0 | 0 | 60940 | 0 | 0 |
| Liquiritin apioside | C26 H30 O13 | 550.1694 | 100 | 0 | 0 | 0 | 132917 | 0 | 0 |
| Diosmin | C28 H32 O15 | 608.1721 | 100 | 0 | 0 | 0 | 28057 | 0 | 0 |
| Hesperidin | C28 H34 O15 | 610.1899 | 100 | 0 | 0 | 0 | 56153 | 0 | 0 |
| 2,3-Dehydrosilybin | C25 H20 O10 | 480.105 | 100 | 0 | 0 | 0 | 6862 | 0 | 0 |
| Isoflavone 7-O-beta-D-glucoside | C21 H20 O8 | 400.1163 | 100 | 0 | 0 | 0 | 34993 | 0 | 0 |
| threo-Syringoylglycerol | C11 H16 O6 | 244.094 | 96.2 | 0 | 0 | 0 | 157413 | 0 | 0 |
| Dihydrocapsiate | C18 H28 O4 | 308.1986 | 96.1 | 0 | 0 | 0 | 61554 | 0 | 0 |
| 6-Hydroxyshogaol | C17 H24 O4 | 292.1678 | 100 | 0 | 0 | 0 | 544291 | 463454 | 2693242 |
| Isorhamnetin 3-O-[b-D-xylopyranosyl-(1->6)-b-D-glucopyranoside] | C27 H30 O16 | 610.1532 | 100 | 0 | 0 | 0 | 92893 | 17545 | 71559 |
| Quercetin 3-(2G-xylosylrutinoside) | C32 H38 O20 | 742.1929 | 100 | 0 | 0 | 0 | 0 | 14549 | 50905 |
| Sophoraisoflavanone A | C21 H22 O6 | 370.1386 | 100 | 0 | 0 | 0 | 0 | 51365 | 0 |
| cis-Ferulic acid [arabinosyl-(1->3)-[glucosyl-(1->6)]-glucosyl] ester | C27 H38 O18 | 650.2055 | 100 | 0 | 0 | 0 | 0 | 13786 | 0 |
| Genistein 4',7-O-diglucuronide | C27 H26 O17 | 622.1244 | 96.3 | 0 | 0 | 0 | 0 | 15355 | 0 |
| Syringetin | C17 H14 O8 | 346.0719 | 100 | 0 | 0 | 0 | 0 | 37255 | 0 |
| Quercetin 3-O-glucosyl-rutinoside | C33 H40 O21 | 772.2062 | 100 | 0 | 0 | 0 | 0 | 46910 | 20660 |
| Gancaonin X | C21 H22 O4 | 338.1517 | 100 | 0 | 0 | 0 | 0 | 169223 | 0 |
| 4-O-beta-D-Glucosyl-4-hydroxycinnamate | C15 H18 O8 | 326.1008 | 100 | 0 | 0 | 0 | 64753 | 164152 | 0 |
| 9-Hydroxy-4-methoxypsoralen 9-glucoside | C18 H18 O10 | 394.0884 | 96.4 | 0 | 0 | 0 | 0 | 398834 | 0 |
| 1-O-Feruloyl-β-D-glucose | C16 H20 O9 | 356.1117 | 100 | 0 | 0 | 0 | 0 | 40579 | 285529 |
| Herbacetin 3,8-diglucoside | C27 H30 O17 | 626.1487 | 100 | 0 | 0 | 0 | 0 | 27991 | 0 |
| Kazinol A | C25 H30 O4 | 394.2142 | 99.1 | 0 | 0 | 0 | 0 | 193182 | 0 |
| 3-Methylquercetin 7-[galactosyl-(1->4)-glucoside] | C28 H32 O17 | 640.1648 | 100 | 0 | 0 | 0 | 0 | 28865 | 0 |
| 1-O-Sinapoyl-β-D-glucose | C17 H22 O10 | 386.1219 | 100 | 0 | 0 | 0 | 0 | 97775 | 0 |
| 2''-O-Acetylrutin | C29 H32 O17 | 652.1639 | 100 | 0 | 0 | 0 | 0 | 168998 | 0 |
| 5'-Demethoxydeoxypodophyllotoxin | C21 H20 O6 | 368.1291 | 99.8 | 0 | 0 | 0 | 0 | 46784 | 0 |
| (+)-Syringaresinol O-beta-D-glucoside | C28 H36 O13 | 580.2186 | 100 | 301472 | 0 | 0 | 0 | 37630 | 0 |
| 4'-O-Methylglucoliquiritigenin | C22 H24 O9 | 432.1407 | 100 | 0 | 0 | 0 | 0 | 65655 | 0 |
| Quercetin 3-sambubioside | C26 H28 O16 | 596.1383 | 97.3 | 0 | 0 | 0 | 0 | 21513 | 0 |
| Prupaside | C27 H36 O12 | 552.2193 | 100 | 0 | 0 | 0 | 0 | 156378 | 0 |
| 6''-O-Acetyldaidzin | C23 H22 O10 | 458.122 | 96.9 | 0 | 0 | 0 | 0 | 15378 | 0 |
| Kaempferol 3-xylosylglucoside | C26 H28 O15 | 580.1436 | 100 | 0 | 0 | 0 | 0 | 224516 | 77697 |
| Chavicol | C9 H10 O | 134.0688 | 100 | 0 | 0 | 0 | 0 | 293706 | 0 |
| Myricitrin | C21 H20 O12 | 464.0964 | 100 | 0 | 0 | 0 | 0 | 65576 | 0 |
| 4'-O-Methyl-(-)-epicatechin 3'-O-glucuronide | C22 H24 O12 | 480.1275 | 99.3 | 0 | 0 | 0 | 0 | 90034 | 0 |
| (-)-Epigallocatechin 3-(4-methyl-gallate) | C23 H20 O11 | 472.1015 | 100 | 0 | 0 | 0 | 0 | 15671 | 0 |
| Isoorientin 6''-rhamnoside | C27 H30 O15 | 594.1591 | 100 | 0 | 0 | 0 | 0 | 66851 | 72780 |
| Eujambolin | C24 H24 O13 | 520.1231 | 98.3 | 0 | 0 | 0 | 0 | 15339 | 0 |
| Luteolin | C15 H10 O6 | 286.0486 | 100 | 0 | 0 | 0 | 0 | 649709 | 0 |
| Astragalin | C21 H20 O11 | 448.1015 | 100 | 0 | 0 | 0 | 0 | 3857407 | 13201 |
| Pollenin B | C22 H22 O12 | 478.1115 | 100 | 0 | 0 | 0 | 0 | 42111 | 39191 |
| Eucommin A | C27 H34 O12 | 550.2045 | 100 | 0 | 0 | 0 | 0 | 105228 | 0 |
| Kanzonol N | C22 H24 O6 | 384.1596 | 100 | 0 | 0 | 0 | 0 | 96070 | 0 |
| Poncirin | C28 H34 O14 | 594.2005 | 100 | 0 | 0 | 0 | 0 | 83685 | 0 |
| 3',4',5-Trihydroxy-3,7-dimethoxyflavone 5-glucoside | C23 H24 O12 | 492.1275 | 99.9 | 0 | 0 | 0 | 0 | 254935 | 0 |
| 3,4-Dicaffeoyl-1,5-quinolactone | C25 H22 O11 | 498.1157 | 100 | 0 | 0 | 0 | 0 | 45430 | 0 |
| (7R*,8R*)-3-Methoxy-3',4,7,9,9'-pentahydroxy-8,4'-oxyneolignan 4-xyloside | C24 H32 O11 | 496.1952 | 100 | 0 | 0 | 0 | 0 | 192729 | 0 |
| Natsudaidain 3-(4-O-3-hydroxy-3-methylglutaroylglucoside) | C33 H40 O18 | 724.2223 | 100 | 0 | 0 | 0 | 0 | 21494 | 0 |
| Irisolidone 7-O-glucuronide | C23 H22 O12 | 490.1116 | 100 | 0 | 0 | 0 | 0 | 52852 | 0 |
| Icariin | C33 H40 O15 | 676.2367 | 96.9 | 0 | 0 | 0 | 0 | 36277 | 0 |
| Justicidin A | C22 H18 O7 | 394.1037 | 100 | 0 | 0 | 0 | 0 | 189509 | 0 |
| 1,3-Dicaffeoylquinic acid | C25 H24 O12 | 516.1278 | 100 | 0 | 0 | 0 | 0 | 95435 | 0 |
| Hesperetin 7-O-glucuronide | C23 H24 O11 | 476.1376 | 99 | 0 | 0 | 0 | 0 | 7741 | 0 |
| 2,3-Dihydro-2-(4-hydroxyphenyl)-5,6,7,8-tetramethoxy-4H-1-benzopyran-4-one | C19 H20 O7 | 360.1198 | 100 | 0 | 0 | 0 | 0 | 92047 | 0 |
| 2',7-Dihydroxy-4'-methoxy-8-prenylflavan 2',7-diglucoside | C33 H44 O14 | 664.2741 | 100 | 0 | 0 | 0 | 0 | 15172 | 0 |
| Kaempferol 3-[2''-glucosyl-6''-acetyl-galactoside] 7-glucoside | C35 H42 O22 | 814.2201 | 96.7 | 0 | 0 | 0 | 0 | 19432 | 0 |
| 6-Caffeoylsucrose | C21 H28 O14 | 504.1485 | 100 | 0 | 0 | 0 | 0 | 121376 | 0 |
| 4-Hydroxycoumarin | C9 H6 O3 | 162.032 | 100 | 0 | 0 | 0 | 0 | 0 | 118219 |
| 3-(1,1-Dimethylallyl)scopoletin 7-glucoside | C21 H26 O9 | 422.1569 | 100 | 0 | 0 | 0 | 0 | 0 | 16880 |
| 7-Hydroxyenterolactone | C18 H18 O5 | 314.1156 | 100 | 0 | 0 | 0 | 0 | 0 | 76203 |
| p-Coumaroyl quinic acid | C16 H18 O8 | 338.1003 | 98.9 | 0 | 0 | 0 | 0 | 58447 | 17418 |
| Polydine | C20 H22 O10 | 422.1195 | 100 | 0 | 0 | 0 | 0 | 0 | 192182 |
| Scoparin 2''-glucoside | C28 H32 O16 | 624.1697 | 100 | 0 | 0 | 0 | 0 | 0 | 55066 |
| Popul+A4:A50in | C20 H22 O8 | 390.1324 | 100 | 42315 | 0 | 0 | 0 | 83105 | 0 |
| 1-O-E-Cinnamoyl-(6-arabinosylglucose) | C20 H26 O11 | 442.1485 | 100 | 85287 | 0 | 0 | 57712 | 136312 | 0 |
| 1,2-Bis-O-sinapoyl-beta-D-glucoside | C28 H32 O14 | 592.1783 | 100 | 72789 | 0 | 0 | 0 | 0 | 0 |
| Isopentyl gentiobioside | C17 H32 O11 | 412.1951 | 100 | 323523 | 0 | 0 | 0 | 270659 | 9937 |
| Methyl (R)-9-hydroxy-10-undecene-5,7-diynoate glucoside | C18 H24 O8 | 368.1463 | 96.8 | 28404 | 0 | 0 | 0 | 124909 | 0 |
| 9-Hydroxy-7-megastigmen-3-one glucoside | C19 H32 O7 | 372.2159 | 100 | 578563 | 0 | 0 | 428158 | 6702336 | 0 |
| 6-Epi-7-isocucurbic acid glucoside | C18 H30 O8 | 374.195 | 100 | 509805 | 0 | 0 | 0 | 508158 | 0 |
| (3S,7E,9S)-9-Hydroxy-4,7-megastigmadien-3-one 9-glucoside | C19 H30 O7 | 370.1977 | 100 | 201756 | 64005 | 0 | 0 | 247953 | 42830 |
| 1-Octen-3-yl glucoside | C14 H26 O6 | 290.1732 | 100 | 80366 | 0 | 0 | 0 | 111643 | 21421 |
| Blumenol C O-[apiosyl-(1->6)-glucoside] | C24 H40 O11 | 504.2596 | 100 | 52187 | 0 | 0 | 0 | 0 | 0 |
| Crosatoside B | C20 H30 O11 | 446.1798 | 100 | 124337 | 0 | 0 | 0 | 0 | 75505 |
| Gastrodin | C13 H18 O7 | 286.1045 | 100 | 65229 | 67536 | 0 | 0 | 160905 | 0 |
| Sesamolinol 4'-O-b-D-glucosyl (1->6)-O-b-D-glucoside | C32 H40 O17 | 696.2298 | 97.9 | 82688 | 0 | 0 | 0 | 29258 | 0 |
| 6-(2-Carboxyethyl)-7-hydroxy-2,2-dimethyl-4-chromanone glucoside | C20 H26 O10 | 426.1493 | 96.9 | 36654 | 36241 | 0 | 0 | 0 | 0 |
| Sesaminol glucosyl-(1->2)-[glucosyl-(1->6)]-glucoside | C38 H48 O22 | 856.2638 | 96.4 | 10340 | 0 | 0 | 0 | 51156 | 0 |
| D-Gal α 1->6D-Gal α 1->6D-Glucose | C18 H32 O16 | 504.1696 | 100 | 499456 | 0 | 0 | 0 | 0 | 0 |
| Palatinose | C12 H22 O11 | 342.1175 | 100 | 14298315 | 0 | 0 | 1360749 | 605176 | 16915964 |
| 1-O-Methyl-myo-inositol | C7 H14 O6 | 194.079 | 100 | 78387 | 0 | 0 | 0 | 0 | 0 |
| Dambonitol | C8 H16 O6 | 208.0954 | 96.3 | 393573 | 0 | 0 | 0 | 0 | 0 |
| (x)-1,2-Propanediol 1-O-b-D-glucopyranoside | C9 H18 O7 | 238.1049 | 100 | 121994 | 0 | 0 | 0 | 0 | 78492 |
| Verbasoside | C20 H30 O12 | 462.1703 | 100 | 43044 | 0 | 0 | 0 | 134579 | 0 |
| 7-Hydroxy-2-methyl-4-oxo-4H-1-benzopyran-5-carboxylic acid 7-glucoside | C17 H18 O10 | 382.0888 | 100 | 194639 | 0 | 0 | 0 | 0 | 0 |
| 2,3-Butanediol glucoside | C10 H20 O7 | 252.1219 | 96.5 | 236361 | 0 | 0 | 395134 | 0 | 630905 |
| Salidroside | C14 H20 O7 | 300.1209 | 100 | 183575 | 0 | 0 | 113606 | 0 | 44924 |
| (S)-Mandelic acid O-beta-D-Glucopyranoside | C14 H18 O8 | 314.1007 | 100 | 47034 | 0 | 0 | 0 | 855625 | 0 |
| (2S)-2-Butanol O-[b-D-Apiofuranosyl-(1->6)-b-D-glucopyranoside] | C15 H28 O10 | 368.1692 | 100 | 173981 | 0 | 0 | 0 | 0 | 0 |
| Zizybeoside II | C25 H38 O16 | 594.2166 | 100 | 33313 | 0 | 0 | 45534 | 85488 | 19494 |
| Isolindleyin | C23 H26 O11 | 478.1464 | 100 | 23759 | 0 | 0 | 0 | 0 | 0 |
| Jasmolone glucoside | C17 H26 O7 | 342.1665 | 100 | 739710 | 0 | 0 | 0 | 0 | 0 |
| Phloroacetophenone 6'-[xylosyl-(1->6)-glucoside] | C21 H30 O13 | 490.1692 | 100 | 51769 | 0 | 0 | 0 | 0 | 0 |
| Cymorcin diglucoside | C22 H34 O12 | 490.2042 | 96.6 | 41142 | 0 | 0 | 0 | 122152 | 0 |
| (-)-menthyl beta-D-glucoside | C16 H30 O6 | 318.2048 | 100 | 236455 | 0 | 0 | 115534 | 0 | 0 |
| 5''-(4-Hydroxy-(E)-cinnamoyl) alpha-L-arabinofuranosyl-(1->3)-beta-D-xylopyranosyl-(1->4)-D-xylopyranoside | C24 H32 O15 | 560.1755 | 100 | 1188861 | 0 | 0 | 124489 | 702281 | 57090 |
| 2-Phenylethyl beta-D-glucopyranoside | C14 H20 O6 | 284.1266 | 99.8 | 49165 | 85628 | 0 | 274494 | 418323 | 0 |
| Zizybeoside I | C19 H28 O11 | 432.164 | 100 | 304522 | 99614 | 112207 | 48916 | 0 | 58630 |
| Cyclocalopin D | C23 H32 O13 | 516.1758 | 100 | 109728 | 0 | 0 | 0 | 0 | 0 |
| Palmatoside G | C25 H32 O10 | 492.2011 | 100 | 49749 | 0 | 0 | 0 | 0 | 0 |
| Taraxinic acid glucosyl ester | C21 H28 O9 | 424.1728 | 100 | 72030 | 0 | 0 | 0 | 2139229 | 0 |
| Acanthoside D | C34 H46 O18 | 742.2691 | 100 | 335093 | 0 | 0 | 0 | 234960 | 0 |
| Lippioside I | C25 H30 O13 | 538.1692 | 100 | 52999 | 0 | 0 | 0 | 0 | 0 |
| 11,13-Dihydrotaraxinic acid glucosyl ester | C21 H30 O9 | 426.1879 | 96.9 | 191531 | 0 | 0 | 0 | 0 | 0 |
| (Z)-Resveratrol 3,4'-diglucoside | C26 H32 O13 | 552.1854 | 96.4 | 115677 | 0 | 0 | 0 | 0 | 0 |
| Gingerglycolipid A | C33 H56 O14 | 676.3649 | 100 | 146692 | 125545 | 56300 | 0 | 0 | 0 |
| Ethyl 7-epi-12-hydroxyjasmonate glucoside | C20 H32 O9 | 416.203 | 99.3 | 0 | 103578 | 0 | 0 | 0 | 0 |
| Citrusin C | C16 H22 O7 | 326.1351 | 100 | 0 | 1304516 | 0 | 0 | 1249512 | 0 |
| Biflorin | C16 H18 O9 | 354.0936 | 100 | 161802 | 51760 | 0 | 0 | 0 | 33759 |
| Asperuloside tetraacetate | C26 H30 O15 | 582.1575 | 100 | 0 | 12112 | 16687 | 141343 | 32983 | 14867 |
| 7,8-Dihydro-3b,6a-dihydroxy-alpha-ionol 9-[apiosyl-(1->6)-glucoside] | C24 H42 O12 | 522.267 | 99 | 0 | 31664 | 0 | 0 | 0 | 51222 |
| 5,7-Megastigmadien-9-ol glucoside | C19 H32 O6 | 356.2183 | 100 | 0 | 34391 | 0 | 0 | 0 | 0 |
| 3,6-Anhydroglucose | C6 H10 O5 | 162.0533 | 100 | 0 | 279858 | 0 | 0 | 0 | 0 |
| Secogalioside | C17 H24 O12 | 420.126 | 100 | 0 | 69757 | 0 | 0 | 20058 | 0 |
| Linalool 3,7-oxide beta-primeveroside | C21 H36 O11 | 464.2269 | 98.1 | 0 | 24039 | 44471 | 0 | 0 | 0 |
| Pantoyllactone glucoside | C12 H20 O8 | 292.1167 | 100 | 0 | 0 | 176844 | 0 | 380263 | 106303 |
| Nicotinate D-ribonucleoside | C11 H14 N O6 | 256.0818 | 100 | 0 | 0 | 111475 | 0 | 0 | 0 |
| N-D-Glucosylarylamine | C12 H17 N O5 | 255.1095 | 96.1 | 0 | 0 | 140753 | 0 | 0 | 0 |
| Glucosyl 6-hydroxy-2,6-dimethyl-2E,7-octadienoate | C16 H26 O8 | 346.1612 | 100 | 0 | 0 | 32496 | 0 | 0 | 0 |
| Benzyl O-[arabinofuranosyl-(1->6)-glucoside] | C18 H26 O10 | 402.1514 | 100 | 0 | 0 | 133931 | 0 | 0 | 0 |
| Butyl (S)-3-hydroxybutyrate glucoside | C14 H26 O8 | 322.163 | 100 | 0 | 0 | 0 | 513334 | 0 | 0 |
| Ethyl (S)-3-hydroxybutyrate glucoside | C12 H22 O8 | 294.132 | 100 | 0 | 0 | 0 | 53741 | 0 | 0 |
| 5a,6a-Epoxy-7E-megastigmene-3b,9e-diol 9-glucoside | C19 H32 O8 | 388.21 | 100 | 0 | 0 | 0 | 871704 | 224719 | 99399 |
| (R)-1-O-b-D-glucopyranosyl-1,3-octanediol | C14 H28 O7 | 308.1834 | 100 | 0 | 0 | 0 | 106460 | 88652 | 0 |
| Blumenol C O-[rhamnosyl-(1->6)-glucoside] | C25 H42 O11 | 518.2707 | 100 | 0 | 0 | 0 | 413162 | 112277 | 0 |
| Norrubrofusarin 6-beta-gentiobioside | C26 H30 O15 | 582.1582 | 97.7 | 0 | 0 | 0 | 49767 | 0 | 0 |
| Prenyl arabinosyl-(1->6)-glucoside | C16 H28 O10 | 380.1683 | 96.2 | 0 | 0 | 0 | 55361 | 0 | 0 |
| Swertiamarin | C16 H22 O10 | 374.1219 | 100 | 0 | 0 | 0 | 62765 | 0 | 0 |
| Mithramycin DK | C52 H74 O24 | 1082.4626 | 96.6 | 0 | 0 | 0 | 109058 | 0 | 0 |
| Garcimangosone D | C19 H20 O9 | 392.1109 | 100 | 0 | 0 | 0 | 96675 | 0 | 18890 |
| Acetyl-maltose | C14 H24 O12 | 384.1271 | 100 | 0 | 0 | 0 | 303506 | 0 | 0 |
| D-Thevetose | C7 H14 O5 | 178.0832 | 96.2 | 0 | 0 | 0 | 108835 | 0 | 0 |
| Quinic acid | C7 H12 O6 | 192.0633 | 97.6 | 0 | 0 | 0 | 118030 | 0 | 0 |
| 2'-Methoxy-3-(2,4-dihydroxyphenyl)-1,2-propanediol 4'-glucoside | C16 H24 O9 | 360.1422 | 96.6 | 0 | 0 | 0 | 70955 | 0 | 0 |
| 2-Methoxycarbonylphenyl beta-D-glucopyranoside | C14 H18 O8 | 314.1009 | 98.6 | 0 | 0 | 0 | 103852 | 0 | 0 |
| Ptelatoside A | C19 H26 O10 | 414.153 | 100 | 0 | 0 | 0 | 48675 | 0 | 0 |
| Furocoumarinic acid glucoside | C17 H18 O9 | 366.0936 | 97.8 | 0 | 0 | 0 | 74813 | 0 | 0 |
| (3x,5x,10x)-9,10-Didehydroisohumbertiol O-[rhamnosyl-(1->4)-rhamnosyl-(1->2)-[rhamnosyl-(1->6)]-glucoside] | C39 H62 O18 | 818.3937 | 96.3 | 0 | 0 | 0 | 21105 | 0 | 0 |
| Isopentyl beta-D-glucoside | C11 H22 O6 | 250.1417 | 100 | 0 | 0 | 0 | 83329 | 85449 | 0 |
| Phenylethyl primeveroside | C19 H28 O10 | 416.1674 | 100 | 0 | 0 | 0 | 57762 | 102064 | 0 |
| (S)-Nerolidol 3-O-[a-L-rhamnopyranosyl-(1->4)-a-L-rhamnopyranosyl-(1->6)-b-D-glucopyranoside] | C33 H56 O14 | 676.3652 | 100 | 0 | 0 | 0 | 408169 | 87396 | 0 |
| Dihydrocaffeic acid 3-O-glucuronide | C15 H18 O10 | 358.0905 | 96.4 | 0 | 0 | 0 | 3555445 | 0 | 0 |
| D-Sorbitol | C6 H14 O6 | 182.0791 | 100 | 0 | 0 | 0 | 0 | 238039 | 0 |
| Antirrhinoside | C15 H22 O10 | 362.1208 | 100 | 0 | 0 | 0 | 0 | 49562 | 124304 |
| 3-(3,4-Dihydroxyphenyl)-1-propanol 3'-glucoside | C15 H22 O8 | 330.1292 | 96.2 | 0 | 0 | 0 | 0 | 205177 | 0 |
| Glucosylisomaltol | C12 H16 O8 | 288.0853 | 100 | 0 | 0 | 0 | 0 | 75311 | 100668 |
| Lucuminic acid | C19 H26 O12 | 446.1427 | 100 | 0 | 0 | 0 | 0 | 264256 | 0 |
| Oleandrose | C7 H14 O4 | 162.0893 | 100 | 0 | 0 | 0 | 244578 | 340045 | 0 |
| D-Linalool 3-(6''-malonylglucoside) | C19 H30 O9 | 402.1899 | 100 | 0 | 103899 | 0 | 0 | 11402811 | 0 |
| 1-(beta-D-Glucopyranosyloxy)-3-octanone | C14 H26 O7 | 306.1675 | 100 | 0 | 0 | 0 | 0 | 137021 | 0 |
| Plumieride | C21 H26 O12 | 470.1425 | 100 | 0 | 0 | 0 | 0 | 39205 | 0 |
| Xanthotoxol glucoside | C17 H16 O9 | 364.0792 | 100 | 0 | 0 | 0 | 0 | 88040 | 0 |
| Syringin | C17 H24 O9 | 372.1429 | 100 | 0 | 0 | 0 | 0 | 299214 | 0 |
| Corchoionoside B | C19 H28 O9 | 400.1733 | 100 | 0 | 0 | 0 | 0 | 75206 | 0 |
| Urolithin B 3-O-glucuronide | C19 H16 O9 | 388.0803 | 100 | 0 | 0 | 0 | 0 | 46278 | 0 |
| 6-Feruloylglucose 2,3,4-trihydroxy-3-methylbutylglycoside | C21 H30 O12 | 474.1668 | 100 | 0 | 0 | 0 | 0 | 85041 | 0 |
| Deoxyloganin | C17 H26 O9 | 374.1584 | 100 | 0 | 0 | 0 | 0 | 83014 | 0 |
| Hydroxytyrosol 1-O-glucoside | C14 H20 O8 | 316.1168 | 100 | 0 | 0 | 0 | 0 | 117375 | 0 |
| trans-p-Menthane-7,8-diol 8-glucoside | C16 H30 O7 | 334.1996 | 96.6 | 0 | 0 | 0 | 0 | 144846 | 88220 |
| 5a,6a-Epoxy-7E-megastigmene-3a,9e-diol 3-glucoside | C19 H32 O8 | 388.2101 | 100 | 0 | 0 | 0 | 0 | 682519 | 0 |
| Betulalbuside A | C16 H28 O7 | 332.1843 | 99.1 | 0 | 0 | 0 | 0 | 1385972 | 0 |
| 4-(4-Hydroxyphenyl)-2-butanone O-[2-galloyl-6-cinnamoylglucoside] | C32 H32 O12 | 608.1887 | 100 | 0 | 0 | 0 | 0 | 74182 | 0 |
| Tremulacin | C27 H28 O11 | 528.1634 | 95.6 | 0 | 0 | 0 | 0 | 45032 | 0 |
| 2,4,6-Phenanthrenetriol 2-O-b-D-glucoside | C20 H20 O8 | 388.1165 | 100 | 0 | 0 | 0 | 0 | 34473 | 0 |
| Piceatannol 4'-galloylglucoside | C27 H26 O13 | 558.1371 | 100 | 0 | 0 | 0 | 0 | 65271 | 0 |
| Isorhapontin | C21 H24 O9 | 420.1443 | 100 | 0 | 0 | 0 | 0 | 29578 | 15120 |
| Benzyl beta-primeveroside | C18 H26 O10 | 402.1516 | 100 | 0 | 0 | 0 | 0 | 0 | 85372 |
| Kanokoside A | C21 H32 O12 | 476.1893 | 96 | 0 | 0 | 0 | 0 | 0 | 26128 |
| Abscisic acid glucose ester | C21 H30 O9 | 426.1893 | 100 | 0 | 0 | 0 | 0 | 0 | 84835 |
| Paeonolide | C20 H28 O12 | 460.1552 | 100 | 0 | 0 | 0 | 0 | 0 | 52780 |
| Allose | C6 H12 O6 | 180.0638 | 96.7 | 0 | 0 | 0 | 0 | 0 | 4219620 |
| (E)-2-Methyl-2-buten-1-ol O-beta-D-Glucopyranoside | C11 H20 O6 | 248.1262 | 100 | 0 | 0 | 0 | 0 | 0 | 81495 |
| Oenanthoside A | C16 H20 O8 | 340.1163 | 96.7 | 0 | 0 | 0 | 0 | 0 | 39764 |
| (10S)-Juvenile hormone III diol | C16 H28 O4 | 284.1994 | 100 | 121946 | 0 | 0 | 0 | 0 | 0 |
| 9,10,13-trihydroxy-11-octadecenoic acid | C18 H34 O5 | 330.2416 | 100 | 9926359 | 0 | 0 | 0 | 0 | 0 |
| 6-Hydroxypentadecanedioic acid | C15 H28 O5 | 288.1942 | 100 | 1784626 | 0 | 0 | 888813 | 795315 | 667973 |
| 10,16-dihydroxy-palmitic acid | C16 H32 O4 | 288.2308 | 100 | 204523 | 0 | 0 | 48527 | 0 | 0 |
| Hexadecanedioic acid | C16 H30 O4 | 286.2152 | 100 | 136298 | 0 | 0 | 75932 | 0 | 0 |
| 12-oxo-10E-dodecenoic acid | C12 H20 O3 | 212.1419 | 100 | 33427 | 0 | 0 | 55484 | 26637 | 76276 |
| Avocadyne 2-acetate | C19 H34 O4 | 326.2466 | 100 | 55559 | 0 | 0 | 0 | 0 | 0 |
| α-Linolenic Acid | C18 H30 O2 | 278.2253 | 100 | 265261 | 442508 | 1643882 | 63324 | 60624 | 26970 |
| Linoleic acid | C18 H32 O2 | 280.241 | 96.7 | 29631 | 74922 | 0 | 0 | 194735 | 0 |
| 10,20-Dihydroxyeicosanoic acid | C20 H40 O4 | 344.2938 | 100 | 25909 | 0 | 0 | 0 | 0 | 0 |
| 22-Oxo-docosanoate | C22 H42 O3 | 354.3143 | 100 | 52682 | 0 | 0 | 0 | 0 | 0 |
| 2,4-Heptadecanedione | C17 H32 O2 | 268.2409 | 100 | 66920 | 0 | 0 | 0 | 0 | 0 |
| Traumatic Acid | C12 H20 O4 | 228.1366 | 100 | 0 | 71047 | 0 | 360343 | 0 | 0 |
| Persenone B | C23 H42 O4 | 382.3071 | 100 | 0 | 57268 | 0 | 0 | 0 | 53804 |
| Persenone A | C23 H38 O4 | 378.2761 | 100 | 0 | 99289 | 26117 | 0 | 382962 | 0 |
| Octadecyl fumarate | C22 H40 O4 | 368.2914 | 100 | 0 | 352660 | 0 | 0 | 0 | 0 |
| MG(18:1(9Z)/0:0/0:0) | C21 H40 O4 | 356.2929 | 100 | 0 | 13424 | 0 | 0 | 0 | 0 |
| Methyl (9Z)-8'-oxo-6,8'-diapo-6-carotenoate | C23 H28 O3 | 352.2046 | 100 | 0 | 19318 | 0 | 0 | 0 | 0 |
| Methyl (3x,10R)-dihydroxy-11-dodecene-6,8-diynoate 10-glucoside | C19 H26 O9 | 398.1563 | 98 | 0 | 23489 | 0 | 0 | 0 | 0 |
| Glyceryl lactooleate | C24 H44 O6 | 428.3118 | 99.1 | 0 | 85885 | 289949 | 0 | 0 | 0 |
| Cibaric acid | C18 H28 O5 | 324.1922 | 100 | 66273 | 668148 | 0 | 137993 | 0 | 0 |
| Allyl hexenoate | C9 H14 O2 | 154.0998 | 100 | 0 | 181017 | 171139 | 47957 | 0 | 0 |
| TG(8:0/8:0/8:0) | C27 H50 O6 | 470.3584 | 100 | 0 | 0 | 453770 | 0 | 0 | 0 |
| Prostaglandin D2-1-glyceryl ester | C23 H38 O7 | 426.2599 | 100 | 0 | 0 | 1046535 | 0 | 0 | 0 |
| Pipericine | C22 H41 N O | 335.3186 | 100 | 0 | 0 | 29829 | 0 | 0 | 0 |
| Palmitic amide | C16 H33 N O | 255.2565 | 100 | 0 | 0 | 210793 | 0 | 0 | 0 |
| Momordol | C26 H48 O5 | 440.3481 | 100 | 0 | 0 | 96204 | 0 | 0 | 0 |
| Isobutyl 2-furanpropionate | C11 H16 O3 | 196.1113 | 100 | 0 | 0 | 56622 | 0 | 0 | 0 |
| dodecanamide | C12 H25 N O | 199.1936 | 100 | 0 | 0 | 113624 | 0 | 0 | 0 |
| 8S-HODE | C18 H32 O3 | 296.2342 | 100 | 0 | 0 | 175644 | 0 | 0 | 0 |
| (9S,10S)-10-hydroxy-9-(phosphonooxy)octadecanoic acid | C18 H37 O7 P | 396.2276 | 100 | 0 | 0 | 73521 | 0 | 0 | 0 |
| (±)5,6-DHET | C20 H34 O4 | 338.2442 | 100 | 0 | 0 | 52353 | 0 | 0 | 0 |
| (S)-Mevalonic acid | C6 H12 O4 | 148.0728 | 96.9 | 0 | 0 | 0 | 38393 | 24449 | 0 |
| (9R,10S,12Z)-9,10-Dihydroxy-8-oxo-12-octadecenoic acid | C18 H32 O5 | 328.2252 | 100 | 0 | 0 | 0 | 61950 | 711624 | 63096 |
| (R)-1-O-[b-D-Apiofuranosyl-(1->2)-b-D-glucopyranoside]-1,3-octanediol | C21 H40 O9 | 436.268 | 100 | 0 | 0 | 0 | 62911 | 0 | 0 |
| 16-Oxo-palmitate | C16 H30 O3 | 270.2192 | 100 | 0 | 0 | 0 | 34764 | 0 | 0 |
| Pentadecylic acid | C15 H30 O2 | 242.2244 | 100 | 0 | 0 | 0 | 31723 | 0 | 0 |
| (-)-11-Hydroxy-9,15,16-trioxooctadecanoic acid | C18 H30 O6 | 342.2047 | 100 | 0 | 0 | 0 | 178652 | 358619 | 0 |
| Phloionolic acid | C18 H36 O5 | 332.2568 | 100 | 0 | 0 | 0 | 137448 | 66132 | 0 |
| Tetrahydropersin | C23 H44 O4 | 384.3243 | 100 | 0 | 0 | 0 | 75303 | 0 | 0 |
| 13-Hydroxy-9-methoxy-10-oxo-11-octadecenoic acid | C19 H34 O5 | 342.2407 | 100 | 0 | 0 | 0 | 0 | 281065 | 0 |
| 9E-Heptadecenoic acid | C17 H32 O2 | 268.2404 | 100 | 0 | 0 | 0 | 0 | 56549 | 94060 |
| 1-Hydroxyhexane-1,2,6-tricarboxylate | C9 H14 O7 | 234.075 | 100 | 0 | 0 | 0 | 0 | 0 | 47571 |
| Diisopropyl adipate | C12 H22 O4 | 230.1515 | 100 | 0 | 0 | 0 | 0 | 0 | 42328 |
| 3-Hydroxytetradecanedioic acid | C14 H26 O5 | 274.1784 | 100 | 0 | 0 | 0 | 0 | 0 | 294357 |
| 9S,12S,13S-trihydroxy-10E-octadecenoic acid | C18 H34 O5 | 330.241 | 100 | 0 | 0 | 0 | 0 | 0 | 1754500 |
| Isoachifolidiene | C20 H24 O5 | 344.1607 | 99.7 | 0 | 0 | 0 | 0 | 0 | 30949 |
| Sorbitan palmitate | C22 H42 O6 | 402.2988 | 100 | 0 | 0 | 0 | 0 | 0 | 33205 |
| Piperenol C | C22 H24 O10 | 448.1387 | 100 | 34358 | 0 | 0 | 139978 | 0 | 0 |
| sec-o-Glucosylhamaudol | C21 H26 O10 | 438.1533 | 100 | 130240 | 0 | 0 | 0 | 0 | 0 |
| Curcumin monoglucoside | C27 H30 O11 | 530.18 | 100 | 27592 | 0 | 0 | 0 | 0 | 0 |
| Citronellyl butyrate | C14 H26 O2 | 226.1935 | 99.1 | 89567 | 0 | 0 | 0 | 0 | 0 |
| 9-Oxoasimicinone | C37 H64 O8 | 636.4606 | 100 | 671496 | 0 | 0 | 0 | 0 | 0 |
| Norvisnagin | C12 H8 O4 | 216.0415 | 100 | 67396 | 0 | 0 | 0 | 0 | 0 |
| Caribenolide I | C33 H52 O11 | 624.3521 | 100 | 43030 | 0 | 0 | 0 | 0 | 0 |
| Khellol glucoside | C19 H20 O10 | 408.1044 | 100 | 225824 | 38291 | 0 | 0 | 0 | 0 |
| Ginkgolide C | C20 H24 O11 | 440.1311 | 100 | 85681 | 0 | 0 | 0 | 0 | 0 |
| Vitamin D2 3-glucuronide | C34 H52 O7 | 572.3719 | 96.1 | 0 | 44258 | 0 | 0 | 0 | 0 |
| UWM6 | C19 H18 O6 | 342.1084 | 100 | 0 | 176685 | 0 | 0 | 0 | 0 |
| Stigmatellin Y | C29 H40 O6 | 484.2809 | 100 | 0 | 84139 | 0 | 0 | 0 | 0 |
| Phrymarolin I | C24 H24 O11 | 488.1303 | 98.5 | 0 | 29459 | 0 | 0 | 0 | 0 |
| Oleandolide | C20 H34 O7 | 386.2291 | 100 | 0 | 192175 | 0 | 0 | 0 | 0 |
| Megaphone | C22 H30 O6 | 390.2031 | 100 | 0 | 135544 | 0 | 0 | 0 | 0 |
| Heterophylol | C26 H32 O4 | 408.2315 | 99.5 | 0 | 132923 | 0 | 0 | 0 | 0 |
| Gibberellin A24 | C20 H26 O5 | 346.1768 | 96.6 | 0 | 39648 | 0 | 0 | 0 | 0 |
| Erioflorin methacrylate | C23 H28 O7 | 416.1818 | 100 | 0 | 45565 | 0 | 0 | 0 | 0 |
| Diacetoxyscirpenol | C19 H26 O7 | 366.167 | 96.7 | 0 | 34443 | 0 | 0 | 0 | 0 |
| Cyclocalopin B | C19 H26 O8 | 382.163 | 100 | 0 | 92416 | 83086 | 0 | 0 | 0 |
| Cubebin | C20 H20 O6 | 356.1258 | 100 | 0 | 25604 | 0 | 0 | 0 | 0 |
| Cinnamodial | C17 H24 O5 | 308.1608 | 100 | 0 | 48569 | 0 | 0 | 111491 | 0 |
| Cimifugin | C16 H18 O6 | 306.1089 | 100 | 0 | 485785 | 0 | 0 | 0 | 0 |
| Butyl levulinate | C9 H16 O3 | 172.1114 | 98 | 0 | 75767 | 0 | 0 | 0 | 0 |
| Bovolide | C11 H16 O2 | 180.1157 | 100 | 0 | 54843 | 55620 | 0 | 0 | 0 |
| Avermectin A2a aglycone | C35 H52 O9 | 616.3596 | 100 | 0 | 101153 | 0 | 0 | 0 | 0 |
| Avermectin A1b monosaccharide | C41 H60 O11 | 728.412 | 100 | 0 | 36356 | 0 | 0 | 0 | 0 |
| Anthragallol | C14 H8 O5 | 256.0363 | 100 | 0 | 102815 | 264949 | 0 | 0 | 0 |
| 6,8-Dihydroxy-1,7-diprenylxanthone-2-carboxylic acid | C24 H24 O6 | 408.1563 | 100 | 0 | 607351 | 1285162 | 0 | 0 | 0 |
| 4-Methylumbelliferyl β-D-glucuronide | C16 H16 O9 | 352.0779 | 100 | 0 | 43556 | 0 | 0 | 0 | 0 |
| 3'-Methoxy-[6]-Gingerdiol 3,5-diacetate | C22 H34 O6 | 394.2338 | 98.9 | 0 | 96904 | 0 | 0 | 0 | 0 |
| Valeracetate | C17 H28 O3 | 280.2045 | 100 | 0 | 0 | 23278 | 0 | 0 | 0 |
| Tetracenomycin C | C23 H20 O11 | 472.0981 | 100 | 0 | 0 | 19506 | 0 | 0 | 0 |
| Pinolidoxin | C18 H26 O6 | 338.1717 | 100 | 0 | 0 | 42397 | 0 | 0 | 0 |
| Pectenotoxin 7 | C47 H68 O16 | 888.4475 | 99.3 | 0 | 0 | 95562 | 0 | 0 | 0 |
| Palmidin A | C30 H22 O8 | 510.1351 | 97.3 | 0 | 0 | 11778 | 0 | 0 | 0 |
| Oblongolide | C14 H20 O2 | 220.1459 | 100 | 0 | 0 | 44508 | 0 | 0 | 0 |
| Mangiferin 6'-gallate | C26 H22 O15 | 574.0956 | 100 | 0 | 0 | 89349 | 0 | 0 | 0 |
| Lutein 5,6-epoxide | C40 H56 O3 | 584.4236 | 100 | 0 | 0 | 143898 | 0 | 0 | 0 |
| L-Olivosyl-oleandolide | C26 H44 O10 | 516.2914 | 100 | 0 | 0 | 213591 | 0 | 0 | 0 |
| Ketomyxol/ 2'-Hydroxyflexixanthin | C40 H54 O4 | 598.4033 | 96.6 | 0 | 0 | 82567 | 0 | 0 | 0 |
| Kanokoside C | C27 H42 O17 | 638.2445 | 100 | 0 | 0 | 17173 | 0 | 0 | 0 |
| Garcimangosone B | C24 H24 O6 | 408.1566 | 96.3 | 0 | 0 | 23721 | 0 | 0 | 0 |
| Dioctyl phthalate | C24 H38 O4 | 390.2773 | 100 | 0 | 0 | 243132 | 0 | 0 | 0 |
| Di-n-hexyl phthalate | C20 H30 O4 | 334.2128 | 100 | 0 | 0 | 537616 | 0 | 0 | 0 |
| Deinoxanthin | C40 H54 O3 | 582.4076 | 100 | 0 | 0 | 77390 | 0 | 0 | 0 |
| Cyanidin 3-arabinoside | C20 H19 O10 | 419.0963 | 96.9 | 0 | 0 | 47008 | 0 | 0 | 0 |
| Cornudentanone | C22 H34 O5 | 378.239 | 100 | 0 | 0 | 85978 | 0 | 0 | 0 |
| 3,6-Epoxy-5,5',6,6'-tetrahydro-b,b-carotene-3',5,5',6'-tetrol | C40 H58 O5 | 618.432 | 96.6 | 0 | 0 | 233717 | 0 | 0 | 0 |
| 1,6-Dihydroxy-3,7-dimethoxy-2-(3-methyl-2-butenyl)-8-(2-hydroxy-3-methyl-3-butenyl)-xanthone | C25 H28 O7 | 440.1812 | 100 | 0 | 0 | 168026 | 0 | 0 | 0 |
| 1,2,3,4-Tetrahydro-1-[1-hydroxy-3-(4-hydroxyphenyl)-2-propenyl]-7-methoxy-2,6-naphthalenediol | C20 H22 O5 | 342.1453 | 100 | 0 | 0 | 84701 | 0 | 0 | 0 |
| cis-Coutaric acid | C13 H12 O8 | 296.0543 | 100 | 0 | 0 | 0 | 84797 | 0 | 0 |
| [6]-Gingerdiol 3,5-diacetate | C21 H32 O6 | 380.2191 | 100 | 0 | 0 | 0 | 380887 | 0 | 0 |
| 1-Caffeoyl-5-feruloylquinic acid | C26 H26 O12 | 530.1407 | 100 | 0 | 0 | 0 | 42278 | 0 | 0 |
| clavulone I | C25 H34 O7 | 446.2315 | 100 | 0 | 0 | 0 | 20640 | 0 | 0 |
| Frangulin A | C21 H20 O9 | 416.111 | 100 | 0 | 0 | 0 | 0 | 79384 | 70796 |
| 2-Hydroxyphenylacetic acid | C8 H8 O3 | 152.047 | 100 | 0 | 0 | 0 | 0 | 52864 | 0 |
| 3-Isopropylcatechol | C9 H12 O2 | 152.0841 | 100 | 0 | 0 | 0 | 0 | 89295 | 0 |
| Medicarpin 3-O-glucoside-6'-malonate | C25 H26 O12 | 518.1423 | 100 | 0 | 0 | 0 | 0 | 48401 | 0 |
| Gibberellin A14 | C20 H28 O5 | 348.194 | 100 | 0 | 0 | 0 | 0 | 367587 | 0 |
| 3'-O-methylbatatasin III | C16 H18 O3 | 258.1264 | 100 | 0 | 0 | 0 | 0 | 49726 | 0 |
| Methylsyringin | C18 H26 O9 | 386.1581 | 100 | 0 | 0 | 0 | 0 | 0 | 26841 |
| L-isoleucyl-L-proline | C11 H20 N2 O3 | 228.148 | 96.1 | 772830 | 186073 | 0 | 524551 | 0 | 0 |
| N-(2,3-Dihydroxybenzoyl)-L-serine | C10 H11 N O6 | 241.0562 | 100 | 86427 | 0 | 0 | 0 | 0 | 0 |
| N-(1-Deoxy-1-fructosyl)proline | C11 H19 N O7 | 277.1145 | 96.3 | 86021 | 0 | 0 | 0 | 0 | 0 |
| N-(5-Methyl-3-oxohexyl)alanine | C10 H19 N O3 | 201.1368 | 100 | 806233 | 0 | 0 | 0 | 1550048 | 0 |
| N-Carbamoyl-L-aspartic acid | C5 H8 N2 O5 | 176.0442 | 100 | 111869 | 0 | 238174 | 0 | 265001 | 0 |
| N(alpha)-t-Butoxycarbonyl-L-leucine | C11 H21 N O4 | 231.1477 | 100 | 160362 | 0 | 0 | 0 | 0 | 0 |
| Histidinyl-Histidine | C12 H16 N6 O3 | 292.1291 | 100 | 140999 | 75538 | 0 | 0 | 0 | 0 |
| Deoxyguanosine | C10 H13 N5 O4 | 267.0989 | 97.7 | 276382 | 0 | 2094104 | 0 | 945250 | 0 |
| 8-Azaadenosine | C9 H12 N6 O4 | 268.093 | 100 | 58624 | 0 | 0 | 0 | 0 | 0 |
| N-(1-Deoxy-1-fructosyl)isoleucine | C12 H23 N O7 | 293.148 | 99.3 | 756398 | 91413 | 1636366 | 0 | 0 | 1429962 |
| Valyl-Isoleucine | C11 H22 N2 O3 | 230.1639 | 100 | 0 | 26464 | 0 | 0 | 0 | 0 |
| Tyrosyl-Tyrosine | C18 H20 N2 O5 | 344.1362 | 100 | 0 | 335721 | 0 | 0 | 0 | 0 |
| Thymine | C5 H6 N2 O2 | 126.043 | 100 | 0 | 74183 | 260602 | 0 | 0 | 0 |
| N-Methyl-L-alanine | C4 H9 N O2 | 103.0635 | 100 | 0 | 157437 | 117917 | 0 | 0 | 0 |
| N-(1-Deoxy-1-fructosyl)phenylalanine | C15 H21 N O7 | 327.1324 | 100 | 0 | 25646 | 537238 | 0 | 0 | 729829 |
| Histidinyl-Asparagine | C10 H15 N5 O4 | 269.1115 | 100 | 0 | 64819 | 0 | 0 | 0 | 0 |
| Histidinyl-Arginine | C12 H21 N7 O3 | 311.1711 | 100 | 0 | 31061 | 0 | 0 | 0 | 0 |
| D-Proline | C5 H9 N O2 | 115.0639 | 96.1 | 0 | 135351 | 0 | 0 | 0 | 0 |
| Arginyl-Gamma-glutamate | C11 H22 N6 O4 | 302.1712 | 100 | 0 | 50500 | 0 | 0 | 0 | 0 |
| Uracil | C4 H4 N2 O2 | 112.0274 | 100 | 0 | 0 | 128237 | 0 | 0 | 0 |
| Thalassemine | C8 H19 N4 O6 P | 298.1032 | 100 | 0 | 0 | 65173 | 0 | 0 | 0 |
| Streptobiosamine | C13 H23 N O9 | 337.1364 | 100 | 0 | 0 | 96098 | 0 | 0 | 0 |
| Slaframine | C10 H18 N2 O2 | 198.1375 | 100 | 0 | 0 | 721136 | 0 | 0 | 0 |
| Serinyl-Phenylalanine | C12 H16 N2 O4 | 252.1111 | 100 | 0 | 0 | 52989 | 0 | 0 | 0 |
| Pseudomonine | C16 H18 N4 O4 | 330.1329 | 100 | 0 | 0 | 40803 | 0 | 0 | 0 |
| N-Carboxyethyl-γ-aminobutyric acid | C7 H13 N O4 | 175.0845 | 99.8 | 0 | 0 | 513012 | 0 | 0 | 0 |
| Na-L-Glutamyl-L-aspartic acid | C9 H14 N2 O7 | 262.0798 | 100 | 0 | 0 | 96501 | 0 | 0 | 0 |
| N-[[3-(b-D-Glucopyranosyloxy)-2,3-dihydro-2-oxo-1H-indol-3-yl]acetyl]aspartic acid | C20 H24 N2 O12 | 484.1352 | 97.6 | 0 | 0 | 116991 | 0 | 0 | 0 |
| N-[(Ethoxycarbonyl)methyl)-p-menthane-3-carboxamide | C15 H27 N O3 | 269.1995 | 100 | 0 | 0 | 51737 | 0 | 0 | 0 |
| N-(1-Deoxy-1-fructosyl)tryptophan | C17 H22 N2 O7 | 366.1438 | 100 | 0 | 0 | 130490 | 0 | 0 | 0 |
| Miraxanthin-III | C17 H18 N2 O5 | 330.1223 | 100 | 0 | 0 | 271216 | 0 | 0 | 0 |
| Luciduline | C13 H21 N O | 207.1617 | 100 | 0 | 0 | 32493 | 0 | 0 | 0 |
| Lupinate | C13 H18 N6 O3 | 306.145 | 100 | 0 | 0 | 52593 | 0 | 0 | 0 |
| Linoleoyl Ethanolamide | C20 H37 N O2 | 323.2826 | 99.9 | 0 | 0 | 188810 | 0 | 0 | 0 |
| Isopentenyladenine | C10 H13 N5 | 203.1168 | 100 | 0 | 0 | 86822 | 0 | 0 | 0 |
| Isoleucyl-Glutamate | C11 H20 N2 O5 | 260.135 | 100 | 0 | 0 | 140243 | 0 | 0 | 0 |
| Guanidinosuccinic Acid | C5 H9 N3 O4 | 175.059 | 100 | 0 | 0 | 72627 | 0 | 0 | 0 |
| 4-(3-Methylbut-2-enyl)-L-tryptophan | C16 H20 N2 O2 | 272.1525 | 100 | 0 | 0 | 66021 | 0 | 0 | 0 |
| Philanthotoxin 343 | C23 H41 N5 O3 | 435.3161 | 98.8 | 0 | 0 | 0 | 165291 | 0 | 0 |
| Prolyl-Histidine | C11 H16 N4 O3 | 252.1218 | 100 | 0 | 0 | 0 | 886846 | 0 | 0 |
| L-gamma-Glutamyl-S-allylthio-L-cysteine | C11 H18 N2 O5 S2 | 322.0669 | 100 | 0 | 0 | 0 | 101045 | 0 | 0 |
| Arginyl-Arginine | C12 H26 N8 O3 | 330.2145 | 100 | 0 | 0 | 0 | 31884 | 0 | 0 |
| 2-Methylaminoadenosine | C11 H16 N6 O4 | 296.124 | 100 | 0 | 0 | 0 | 109761 | 0 | 0 |
| Thymidine | C10 H14 N2 O5 | 242.0907 | 100 | 0 | 0 | 0 | 121560 | 0 | 0 |
| Adenine | C5 H5 N5 | 135.055 | 100 | 0 | 0 | 0 | 0 | 819983 | 0 |
| Deoxyadenosine | C10 H13 N5 O3 | 251.1033 | 100 | 0 | 0 | 0 | 0 | 420060 | 0 |
| Tryptophyl-Tyrosine | C20 H21 N3 O4 | 367.1524 | 100 | 0 | 0 | 0 | 0 | 218069 | 0 |
| Isopentenyl adenosine | C15 H21 N5 O4 | 335.158 | 100 | 0 | 0 | 0 | 0 | 69641 | 0 |
| Tryptophyl-Arginine | C17 H24 N6 O3 | 360.1918 | 100 | 0 | 0 | 0 | 0 | 0 | 72707 |
| N-(1-Deoxy-1-fructosyl)valine | C11 H21 N O7 | 279.1323 | 100 | 0 | 0 | 0 | 0 | 0 | 737250 |
| N(alpha)-Benzyloxycarbonyl-L-leucine | C14 H19 N O4 | 265.1309 | 96.6 | 0 | 0 | 0 | 0 | 0 | 29676 |
| N-Feruloylglycyl-L-phenylalanine | C21 H22 N2 O6 | 398.1485 | 98.1 | 0 | 0 | 0 | 0 | 0 | 25640 |
